# A randomised, placebo-controlled study to assess whether antibiotic-mediated disruption of the gut microbiota impairs BCG-induced specific and non-specific immunity in young, healthy adults: protocol for the Antibiotics and Vaccine Immune Responses Study (AVIRS)

**DOI:** 10.1136/bmjopen-2026-126924

**Published:** 2026-10-14

**Authors:** Natalie E Stevens, Rochelle Botten, James B Geake, Georgina L Eden, Wing Ki Lo, Joseph Inauen, Jelena Solujic, Alice Sawka, Lisa N Yelland, Elisabeth WR Reid, Michelle Shaw, Xiaodan Zhu, Tsz Kin Tam, Hyejin Jeon, Nelly Amenyogbe, Miriam A Lynn, Feargal J Ryan, Carl G Feng, Warwick J Britton, Tobias R Kollmann, Simone E Barry, David J Lynn

**Affiliations:** 1 Systems Immunology Laboratory, South Australian Health and Medical Research Institute, Adelaide, South Australia, Australia; 2 Flinders Health and Medical Research Institute (FHMRI), Flinders University College of Medicine and Public Health, Bedford Park, South Australia, Australia; 3 Clinical Trials Platform, South Australian Health and Medical Research Institute, Adelaide, South Australia, Australia; 4 Department of Thoracic Medicine, Royal Adelaide Hospital, Adelaide, South Australia, Australia; 5 South Australian Tuberculosis Service, Central Adelaide Local Health Network, Adelaide, South Australia, Australia; 6 Faculty of Health and Medical Sciences, Adelaide University, Adelaide, South Australia, Australia; 7 SAHMRI Women and Kids, South Australian Health and Medical Research Institute, North Adelaide, South Australia, Australia; 8 School of Public Health, Adelaide University, Adelaide, South Australia, Australia; 9 Department of Microbiology and Immunology, Dalhousie University, Halifax, Nova Scotia, Canada; 10 Immunology and Host Defence Group, The University of Sydney, Sydney, New South Wales, Australia; 11 Centre for Infection and Immunity, Centenary Institute, Newtown, New South Wales, Australia; 12 The University of Sydney Faculty of Medicine and Health, Sydney, New South Wales, Australia

**Keywords:** IMMUNOLOGY, Vaccination, Gastrointestinal Microbiome, Randomized Controlled Trial, Antibiotics

## Abstract

**Introduction:**

The Bacille Calmette-Guérin (BCG) vaccine protects against severe tuberculosis in children but can also induce non-specific effects including protection against unrelated infections. For reasons that remain unclear, BCG vaccine responses vary widely between individuals, age groups and geographic regions. Emerging evidence suggests the gut microbiota influences vaccine responses but its role in modulating BCG-induced specific and non-specific immunity in humans is unknown.

**Methods and analysis:**

This multiarm, randomised, placebo-controlled experimental medicine study will determine whether depletion of the gut microbiota before vaccination impairs BCG-induced specific and non-specific immunity. We plan to randomise up to 348 healthy, BCG-naïve adults (18–35 years) at a 1:1:1:1 ratio to receive 3 days of oral vancomycin and neomycin (antibiotics group) or no antibiotics (comparator group) prior to BCG (Danish strain 1331) or placebo vaccination. Participants will then undergo a challenge with either intradermal BCG vaccine 6 months postrandomisation, or live-attenuated Yellow Fever Vaccine (YFV) 3 months postrandomisation to assess BCG-induced specific and non-specific protection, respectively. Stool and blood samples will be collected longitudinally to characterise microbiota composition and immune responses. The primary endpoint in the BCG-challenge arm is BCG colony-forming units (CFU) at the dermal challenge site 14 days postchallenge in BCG-vaccinated participants in the antibiotics-treated and comparator groups. The primary outcome in the YFV-challenge arm is viraemia (viral copies/mL plasma) at 3–7 days post YFV challenge in BCG-vaccinated participants in the antibiotic-treated and comparator groups. Secondary endpoints include comparison of BCG CFU and YFV viraemia in BCG- and placebo-vaccinated participants; antimycobacterial T cell and antibody responses in all four randomisation groups in each study arm and innate immune responses to YFV challenge in the YFV-challenge arm. Exploratory analyses will assess the impact of antibiotics on stool microbiota composition, assess the transcriptome, epigenome and immune cell frequency and activation in peripheral blood and measure immune cells and spatially resolved gene expression in selected skin biopsies.

**Ethics and dissemination:**

Ethical approval has been granted by the Central Adelaide Local Health Network (Adelaide, South Australia) and governance approval obtained from Royal Adelaide Hospital and South Australian Health and Medical Research Institute. Results will be published in peer-reviewed open-access journals. Deidentified data will be stored on secure institutional repositories for at least 15 years.

**Trial registration number:**

NCT06148025.

**Strengths and limitations of this study**

- This study combines a randomised, placebo-controlled design with controlled human challenge models enabling causal evaluation of the role of gut microbiota in Bacille Calmette-Guérin (BCG)-induced protection against mycobacterial or viral challenge; the large sample size relative to similar studies supports robust estimation of these effects.
- The randomised design minimises potential confounding between the groups by factors such as age, sex and comorbidities, while recruitment of healthy BCG-naïve adults in a non-tuberculosis (TB)-endemic setting reduces the likelihood of prior BCG vaccination, *Mycobacterium tuberculosis* exposure or TB infection influencing study outcomes.
- The study integrates longitudinal sampling with systems immunology approaches (including metagenomics, transcriptomics, epigenomics and cellular immunology) to enable detailed mechanistic insight into specific and non-specific immune responses to the BCG vaccine.
- A limitation of the study is that blinding to antibiotic allocation is not feasible due to recognisable side effects and the strong impact of antibiotics on the microbiota, and BCG-related skin reactions may limit our ability to blind participants to their assigned vaccination group; however, objective primary outcomes and blinded analyses will minimise bias.
- Another limitation is that the study population is restricted to young, healthy adults in a low TB-endemic setting, which may limit generalisability to populations routinely receiving BCG vaccination (eg, infants or individuals in TB-endemic regions).

## Introduction

 Vaccines provide specific protection against their target diseases by inducing antigen-specific adaptive immunity. However, for reasons that are poorly understood, vaccine-induced immune responses are highly variable across different populations and are frequently suboptimal in vulnerable populations at highest risk of infectious disease.^1–3^ The gut microbiota plays a critical role in regulating antigen-specific immune responses to vaccination.^4–7^ Evidence from animal models, including our own,^8^ has convincingly demonstrated a causal relationship between gut microbial composition and vaccine immunogenicity.^9 10^ Observational clinical studies suggest that this is also the case in human infants. For example, we have recently demonstrated that antibiotic-mediated depletion of bifidobacteria from the infant gut microbiota plays a critical role in mediating the suboptimal antibody responses observed in those infants following routine vaccination.^11^ In adults, two small (9–11 participants per arm) randomised controlled studies have demonstrated that adults randomised to antibiotics have impaired antibody responses to the seasonal influenza^12^ and rabies vaccines,^13^ but only in participants with low pre-existing immunity.

Tuberculosis (TB), caused by *Mycobacterium tuberculosis*, remains one of the world’s leading infectious causes of death. In 2024, an estimated 10.8 million people developed TB and approximately 1.25 million died from the disease worldwide, despite the availability of effective treatment.^14^ The Bacillus Calmette-Guérin (BCG) vaccine—a vaccine containing live-attenuated *Mycobacterium bovis*—remains the only licensed vaccine against TB and is routinely administered to more than 100 million infants annually. Although BCG provides consistent protection against severe forms of childhood TB, its effectiveness against pulmonary TB in adolescents and adults is highly variable.^15 16^ Consistent with this, immune responses to BCG vaccination vary substantially between individuals, age groups and geographic regions,^2 17 18^ and no accepted immunological correlate of protection has been established for BCG to date.^2^ Environmental and microbial exposures, including the gut microbiota, have been increasingly proposed as important factors contributing to this variation.^8 19 20^

In addition to its role in TB prevention, a substantive body of evidence indicates that BCG can also provide non-specific protection against other non-mycobacterial infections, potentially via effects on the innate immune system.^3 21^ Evidence for these non-specific effects is strongest in infants, where observational studies and randomised trials have reported reductions in non-TB infectious disease burden^22 23^ and all-cause mortality^24–26^ following BCG vaccination. However, evidence in adults has been mixed. While some studies have reported reduced susceptibility to unrelated infections following BCG vaccination in adults,^27 28^ multiple large randomised trials have failed to demonstrate consistent clinical protection.^29–32^ Nonetheless, experimental human studies have demonstrated that BCG vaccination induces functional reprogramming of innate immune responses in adults.^33–35^ In a double-blinded study, adults randomised to receive BCG vaccination exhibited reduced viraemia following challenge with the live-attenuated Yellow Fever Vaccine (YFV), and enhanced cytokine responses consistent with innate immune reprogramming.^21^ However, whether the gut microbiome influences these non-specific effects of BCG is almost completely unexplored.

Our study will use two experimental controlled human (attenuated) infection models (CHIMs) to assess whether depletion of the gut microbiota with antibiotics prior to BCG vaccination of young, healthy, BCG-naïve adults leads to (a) impaired mycobacterial-specific immunity (study arm 1) or (b) impaired non-specific immunity (study arm 2). To do this, a cohort of young, healthy, adult volunteers will be randomised to receive a course of oral antibiotics (vancomycin and neomycin), or no antibiotics, prior to vaccination with BCG or placebo (saline). The impact of depleting the gut microbiota on the efficacy of BCG against a secondary intradermal challenge with *M. bovis* BCG (a challenge model previously developed to assess mycobacterial-specific immunity)^36^ or against a live-attenuated virus (a clinical model of BCG-induced non-specific immunity)^21^ will be assessed. Together, these data will provide strong evidence in humans as to whether the gut microbiota regulates pathogen-specific and/or non-specific immunity induced by BCG vaccination.

### Study aims

#### Primary objective

The primary objective of the study is to determine whether antibiotic-mediated depletion of the gut microbiota prior to BCG vaccination leads to impaired BCG-induced specific (antimycobacterial) or non-specific (antiviral) protection against subsequent live-attenuated microbial challenge. This will be assessed in two parallel study arms utilising either an intradermal BCG challenge (mycobacterial) or subcutaneous YFV challenge (viral), with different endpoints:

Primary endpoints (primary outcome measures):

1. BCG-challenge arm:
  Mycobacterial load (CFU/mg tissue) in the skin biopsy from the challenge site at 14 days post-BCG challenge in BCG-vaccinated participants exposed to antibiotics (BCG-ABX) compared with BCG-vaccinated participants who were not exposed to antibiotics (BCG-No ABX).
2. YFV-challenge arm:
  Viraemia (viral copies/mL plasma) at days 3–7 post-YFV challenge in BCG-vaccinated participants exposed to antibiotics (BCG-ABX) compared with BCG-vaccinated participants who were not exposed to antibiotics (BCG-No ABX).

Secondary endpoints (secondary outcome measures):

1. BCG-challenge arm:
  - Bacterial load (16S rRNA gene copies/g stool) in ABX participants compared with No-ABX participants at baseline, day of BCG/placebo vaccination and day of BCG challenge.
  - Microbiota diversity (Shannon diversity index) in ABX participants compared with No-ABX participants at baseline, day of BCG/placebo vaccination and day of BCG challenge.
  - Mycobacterial load (CFU/mg tissue) in the skin biopsy site in BCG-vaccinated participants versus placebo vaccinated participants.
  - Mycobacterial-specific IFNγ responses (number of spot-forming units) in BCG-ABX participants compared with BCG-No ABX participants, assessed at baseline, 2 weeks post-BCG/placebo vaccination, day of BCG challenge and 2 weeks post-BCG challenge.
  - Mycobacterial-specific T-cell activation-induced marker expression (frequency of OX40+ CD137+ CD4+ T cells) in BCG-ABX participants compared with BCG-No ABX participants, assessed at baseline, 2 weeks post-BCG/placebo vaccination, day of BCG challenge and 2 weeks post-BCG challenge.
  - Mycobacterial-specific T-cell activation-induced marker expression (frequency of CD69+ CD137+ cells of CD8+ T cells) in BCG-ABX participants compared with BCG-No ABX participants, assessed at baseline, 2 weeks post-BCG/placebo vaccination, day of BCG challenge and 2 weeks post-BCG challenge.
2. YFV-challenge arm:
  - Bacterial load (16S rRNA gene copies/g stool) in ABX participants compared with No-ABX participants at baseline, day of BCG/placebo vaccination and day of YFV challenge.
  - Microbiota diversity (Shannon diversity index) in ABX participants compared with No-ABX participants at baseline, day of BCG/placebo vaccination and day of YFV challenge.
  - Peak viraemia (viral copies/mL plasma) at days 3–7 post YFV challenge in BCG-vaccinated participants compared with placebo-vaccinated participants, assessed 3, 5 and 7 days following YFV challenge.
  - TNFα cytokine production (pg/mL) following stimulation of peripheral blood mononuclear cell (PBMC) with resiquimod (R848) in BCG-ABX participants compared BCG-No ABX participants, assessed at baseline and day of YFV challenge.
  - TNFα cytokine production (pg/mL) following stimulation of PBMC with lipopolysaccharide (LPS) in BCG-ABX participants compared BCG-No ABX participants, assessed at baseline and day of YFV challenge.
  - TNFα cytokine production (pg/mL) following stimulation of PBMC with heat-killed Candida albicans in BCG-ABX participants compared BCG-No ABX participants, assessed at baseline and day of YFV challenge.

### Exploratory objectives

As exploratory objectives, we will compare all primary and secondary outcomes across all four randomisation groups (ie, BCG-No ABX, BCG-ABX, Placebo-No ABX and Placebo-ABX). We will then use a systems immunology approach to identify components of the gut microbiota, immunological markers and transcriptomic signatures associated with BCG-specific and non-specific immunity.

This will be assessed by longitudinal analysis of the stool microbiota (bacterial load and composition), immune cells in blood (frequency and activation), molecular analysis of proteins, lipids and bacterial metabolites and whole blood transcriptomics across the study. In the BCG-challenge arm, we will also assess the immune response to BCG challenge via histology and immunohistochemistry of the challenge site (skin punch biopsy) in BCG-vaccinated versus placebo-vaccinated participants and will also perform spatial transcriptomics of the BCG rechallenge site in high versus low responders (as assessed by BCG CFU in the biopsy site). In the YFV-challenge arm, we will also assess genome-wide chromatin accessibility in peripheral blood monocytes, neutrophils and NK cells before and after BCG vaccination across all groups and correlate these data with other immunological and virological data.

## Methods and analysis

### Trial design and setting

In this randomised controlled study, BCG-naïve, young, healthy adults at low risk of TB infection (see eligibility criteria) will be recruited in South Australia between 2023 and 2026. The study sites are the South Australian Health and Medical Research Institute and the Royal Adelaide Hospital in Adelaide, South Australia. Participants will be enrolled into two parallel arms: a BCG challenge arm (n=168) and a YFV-challenge arm (n=180). Within each arm, participants will be randomised in a 1:1:1:1 ratio to one of four groups defined by randomisation to no antibiotics/antibiotics and to BCG/placebo vaccination:

Group 1: not antibiotics-treated (comparator), placebo-vaccinated;

Group 2: not antibiotics-treated (comparator), BCG-vaccinated;

Group 3: antibiotics-treated, placebo-vaccinated; and

Group 4: antibiotics-treated, BCG-vaccinated.

Participants randomised to no antibiotic treatment will receive no intervention, while those randomised to antibiotic treatment will receive a 3 day course of oral antibiotics (vancomycin and neomycin) up to the day before vaccination to deplete the gut microbiota. All participants will then receive either a standard adult dose of BCG (0.1 mL, 2–8 x 10^5^ CFU live-attenuated *M. bovis* Danish strain 1331) or placebo (0.1 mL normal saline) vaccination intradermally to the left arm.

Participants will then undergo an attenuated challenge according to the arm of the study into which they are enrolled:

1. In the BCG-challenge arm (n=168), 6 months following randomisation, all participants will receive a standard adult dose (0.1 mL, 2–8 x 10^5^ CFU live-attenuated *M. bovis*) of BCG vaccine intradermally in the right arm (ie, the opposite arm to the initial BCG/placebo vaccination). A punch biopsy of the skin at the BCG-challenge site will be collected 2 weeks following the BCG challenge.
2. In the YFV-challenge arm (n=180), 3 months following randomisation, all participants will receive a standard dose of YFV (≥1000 IU of live-attenuated yellow fever virus 17D) in the right arm.

Blood and stool samples will be collected at baseline and at predefined timepoints throughout the study (figure 1).

**Figure 1:**
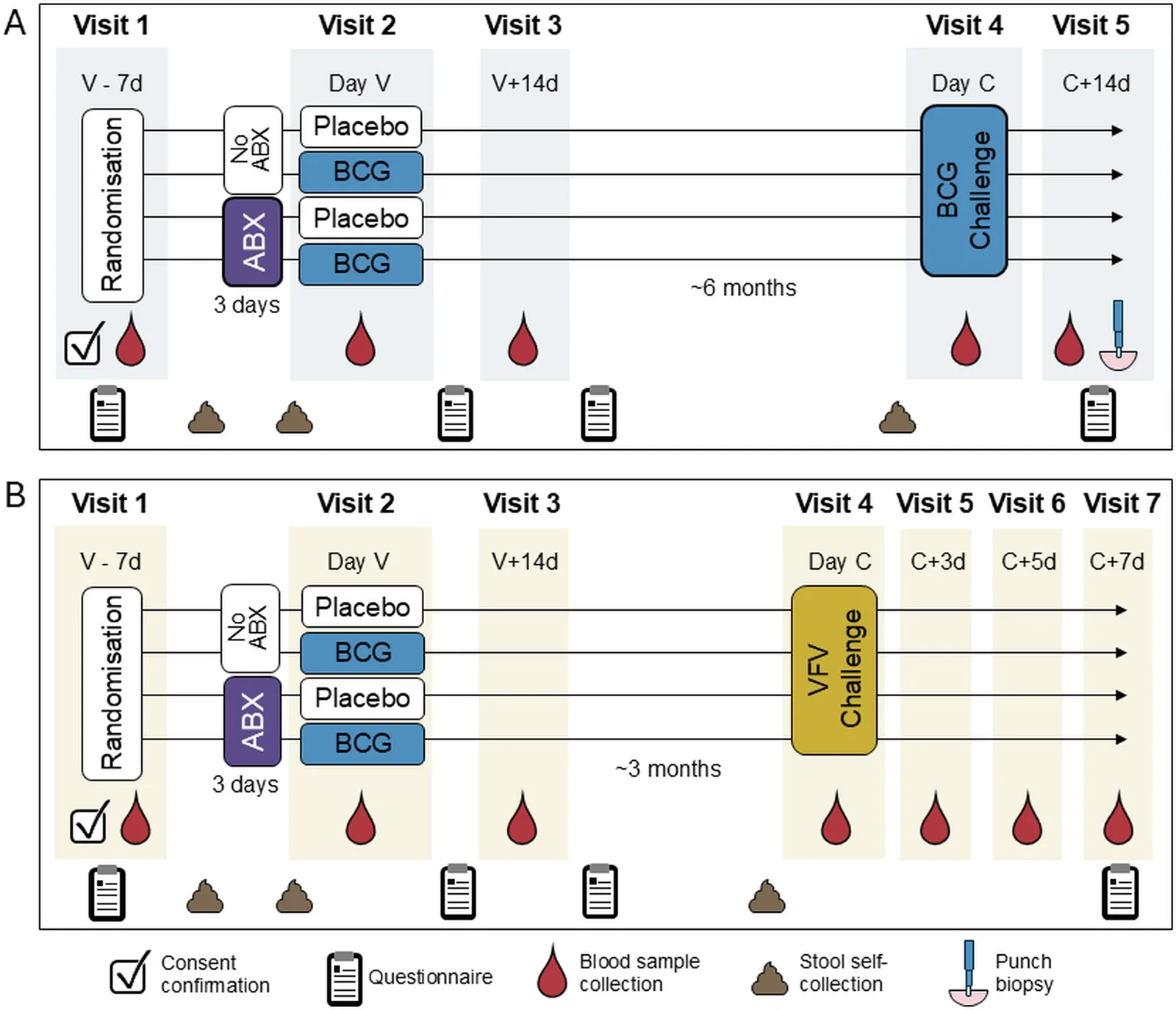
Study visits and sample and data collection in the (**A**) BCG-challenge and (**B**) YFV-challenge study arms. ABX, antibiotics; BCG, Bacille Calmette-Guérin; day C, day of challenge; day V, day of vaccination; d, day; YFV, Yellow Fever Vaccine.

### Trial status

Recruitment commenced in November 2023, with the first participant enrolled on 23 November 2023. Active recruitment will continue until the end of 2026. Recruitment is ongoing at the time of manuscript submission.

### Participants and eligibility criteria

Up to 348 healthy adults will be recruited across both study arms. Eligible participants will be 18–35 years old, BCG-naïve, YFV-naïve (if recruited into the YFV-challenge arm), willing to undergo study procedures, not pregnant or intending to get pregnant for the duration of the study, and able to provide informed consent. Exclusion criteria include:

- Previous BCG vaccination.
- Previous YF vaccination or YF virus infection (only applies to participants included in the YFV challenge substudy).
- Evidence of TB infection (interferon-gamma release assay (IGRA) to confirm if needed).
- Contraindications for BCG vaccination: bone marrow or lymphoid malignancy, primary or secondary immunodeficiency, HIV infection, moderate/severe skin disease (eg, eczema, dermatitis or psoriasis), requires immunosuppressive or immune-modifying drugs.
- Contraindications to YF vaccination: history of thymus disease (eg, myasthenia gravis, thymoma, thymectomy, DiGeorge syndrome, thymic damage from chemoradiotherapy or graft-versus-host disease), immunocompromised, primary immunodeficiencies, require corticosteroids or immunosuppressive drugs, haematopoietic stem cell transplant recipients.
- People who have had a haematopoietic stem cell transplant.
- Individuals with a history of severe allergic reactions to egg or chicken proteins.
- Pregnant or breastfeeding or planning to become pregnant.
- History of renal disease/insufficiency.
- Tattoo obscuring BCG vaccination site(s).
- Any history of severe allergic reaction or anaphylaxis to vaccination or antibiotics.
- People with chronic serious underlying illness.
- Have received any prescribed oral or intravenous antibiotic in the 28 days prior to study visits 1 and 4.
- Have received any vaccinations in the 28 days prior to study visits 1 and 4.

BCG-naïve status will be determined by self-reported vaccination history and country of birth—respondents born in countries and in years where BCG vaccination was recommended for the general population will be considered ineligible—and confirmed by the absence of a BCG vaccination scar at the first study visit. Risk of TB infection will be assessed using a structured epidemiological questionnaire that includes country of birth, residence for more than 3 months in a TB-endemic country, known contact with a person with TB and other relevant epidemiological risk factors. Participants with an uncertain history or identified risk factors will be offered IGRA testing to exclude TB infection, and only participants with a negative IGRA result will be eligible for inclusion. YFV vaccination status will be determined by self-reported vaccination history; in cases where the participant is unsure, self-reported travel history and/or country of birth will be considered to identify possible YFV vaccination. Participants may also be excluded if, in the opinion of the clinical principal investigator, medical history or concomitant medication use poses a clinical risk, including potential interference with the absorption or efficacy of essential medications due to anticipated antibiotic-associated gastrointestinal (GI) side effects.

Participants will be screened for eligibility via an online eligibility questionnaire, and responses reviewed by a study doctor prior to online consent (figure 2, see online supplemental files 1 and 5). A study doctor will confirm eligibility and informed consent at the first study visit prior to randomisation.

**Figure 2:**
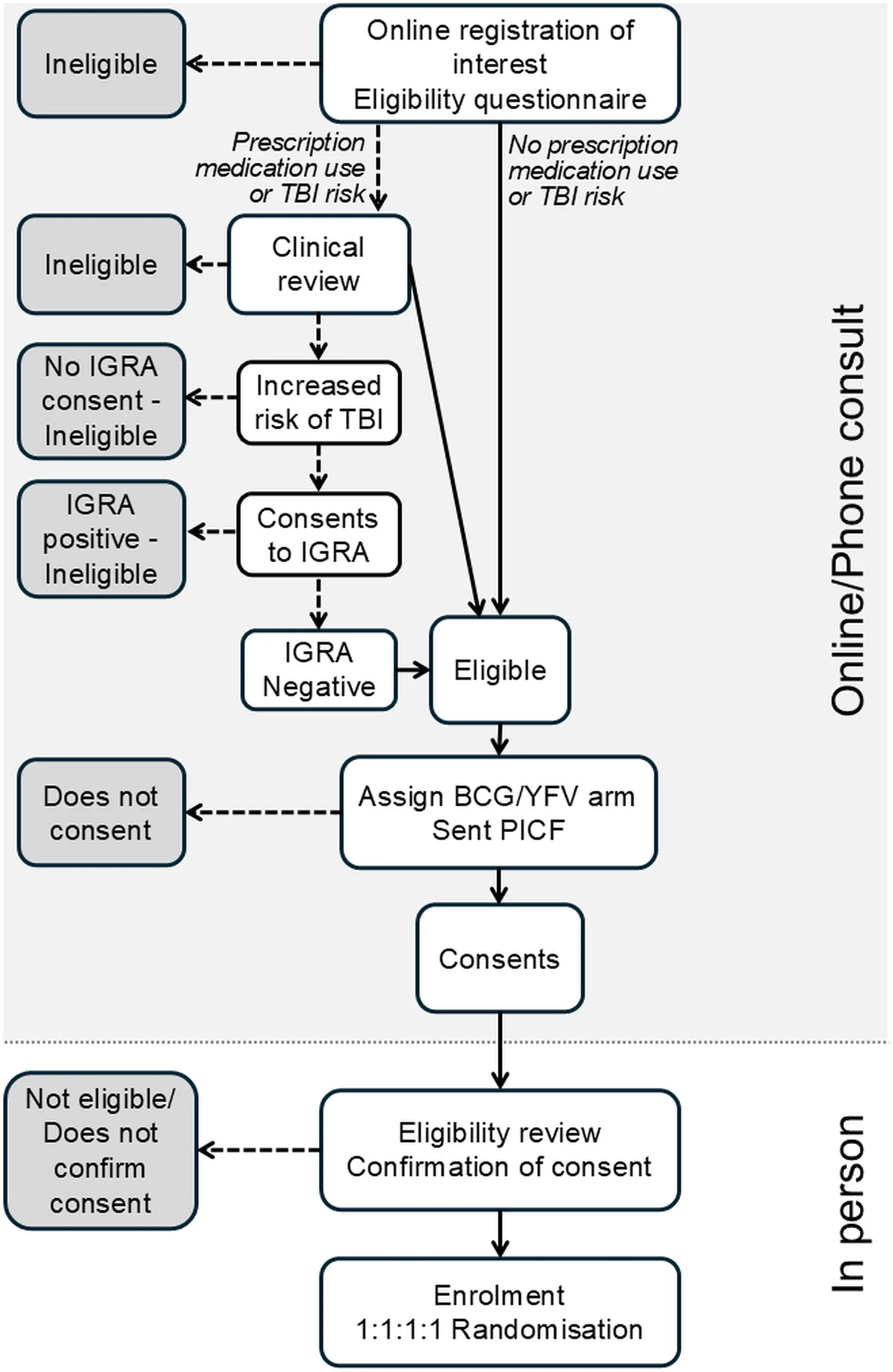
Participant recruitment and consent process. Participants will be screened online, with clinical follow-up as necessary prior to consent, enrolment and randomisation. BCG, Bacille Calmette-Guérin; IGRA, interferon gamma release assay; PICF, participant information and consent form; TBI, tuberculosis infection; YFV, Yellow Fever Vaccine.

### Recruitment

Healthy adults (18–35 years) in South Australia will be recruited from the community, via online channels (institutional emails and newsletters, news articles and social media advertising), print (flyers) or broadcast media (radio and video advertising). Prospective participants will be provided study information (ie, participant communication and the study flyer) directing them to the study website and eligibility questionnaire (see online supplemental material).

### Interventions

#### Antibiotics

Participants randomised to the antibiotic group will receive 500 mg of vancomycin and 1000 mg of neomycin orally every 6 hours for 72 hours. Vancomycin and neomycin are antibiotics with poor systemic absorption which, when delivered orally, reduce gut microbial load while limiting systemic toxicity associated with intravenous administration.^37 38^ Vancomycin is a glycopeptide antibiotic used in the treatment of Gram-positive bacteria. Neomycin is an aminoglycoside antibiotic active against Gram-negative aerobic bacilli and some anaerobic bacilli. Antibiotics will be taken at home. Adherence to the antibiotic regimen will be assessed using a participant-completed medication diary and will be validated by assessing the impact on bacterial load in stool samples collected by the participant before and after antibiotic treatment. GI symptoms in antibiotic-treated participants will be captured in a web-based questionnaire. Participants may also report symptoms or concerns directly to the study team during the antibiotic regimen. Participants reporting that GI symptoms associated with antibiotics are not tolerable may elect to discontinue antibiotic treatment—these participants will be retained in the study cohort and analysed on an intention-to-treat basis.

A no-antibiotic comparator was selected rather than a placebo control because the primary outcomes are objective laboratory measures (bacterial load and immunological parameters), rather than subjective participant-reported outcomes. These objective outcomes are therefore less likely to be influenced by lack of blinding. Blinding participants would also be challenging, as the antibiotic regimen is expected to cause recognisable GI side effects, and the marked reduction in bacterial load in stool samples is likely to reveal antibiotic allocation to the study team. In this context, the use of placebo tablets would increase study complexity and cost, without meaningfully reducing bias in the primary outcomes; therefore, a placebo for the comparator to the antibiotic treatment group was not considered necessary.

To support participants randomised to the antibiotic group in managing anticipated GI symptoms (nausea/diarrhoea), they will be provided with prescriptions for an antiemetic (Metoclopramide) and an antidiarrhoeal agent (Loperamide). Participants randomised to the antibiotic arm will be instructed to take Metoclopramide 10 mg tablets as needed (*pro re nata*; PRN) for nausea/vomiting (up to three times daily for up to 72 hours), and to take Loperamide, PRN, to manage diarrhoea (2 mg every 4 hours or after each loose bowel motion, maximum 16 mg/day, for up to 72 hours).

#### BCG vaccination and BCG challenge

Participants randomised to BCG vaccination will receive a single dose of BCG-Denmark (AJ Vaccines, Copenhagen), 0.1 mL (2–8×10^5^ colony-forming units of *M. bovis*, Danish strain 1331) as an intradermal injection in the upper left arm over the deltoid region, using a 1 mL syringe fitted with a short (10 mm) bevel needle (25–30G). Participants will remain blinded to their intervention group for the duration of their participation in the study.

For the BCG-challenge study arm, a BCG ‘challenge’ vaccination will be given into the upper right arm, using the same vaccine source, dose and administration route as described above.

#### Placebo vaccination

Participants randomised to placebo vaccination will be given a saline placebo intradermal injection in the upper left arm using the same procedure described for BCG and remain blinded to their intervention group for the duration of their participation in the study.

#### YFV vaccination

Participants in the YFV-challenge arm will be given a 0.5 mL dose of reconstituted YFV (containing injectable suspension in stabiliser of >1000 IU of the attenuated yellow fever virus 17D strain) subcutaneously in the right upper arm.

### Randomisation process

A computer-generated randomisation schedule using balanced variable blocks within strata defined by sex will be prepared separately for each arm (BCG- or YFV-challenge arm) by an independent biostatistician (SAHMRI Biostatistics Unit). It will be implemented via the Research Electronic Data Capture platform (REDCap) to allocate participants in the ratio of 1:1:1:1. Participants will be assigned to one of the following four groups:

Group 1: not antibiotics-treated (comparator), placebo-vaccinated.

Group 2: not antibiotics-treated (comparator), BCG-vaccinated.

Group 3: antibiotics-treated, placebo-vaccinated.

Group 4: antibiotics-treated, BCG-vaccinated.

### Blinding

Participants will not be blinded to their antibiotic treatment group but will be blinded to their initial allocated vaccine (BCG/placebo). Staff preparing and administering the intervention (BCG or placebo), and the study coordinator managing clinical visits will be unblinded. Participants, investigators, statisticians and other trial staff involved in follow-up and data collection will be blinded to the vaccine randomisation group.

Unblinding of a participant’s vaccine randomisation group prior to study completion may be permissible in order to manage an AE associated with BCG vaccination, or other compelling medical reason, as determined by the medical monitor or clinical PI. If this occurs, all other investigators will remain blinded.

### Outcomes

#### Primary outcomes

BCG-challenge arm: we will determine whether participants with a depleted gut microbiota, at the time of BCG immunisation, have significantly reduced mycobacterial load following BCG challenge.

YFV-challenge arm: we will determine whether participants with a depleted gut microbiota, at the time of BCG immunisation, have significantly reduced viraemia following challenge with a live attenuated virus (YFV).

These data will provide robust clinical evidence as to whether the gut microbiota regulates the pathogen-specific and non-specific immunity induced by the BCG vaccine.

#### Secondary and exploratory outcomes

Analyses of stool samples using metagenomics will enable us to determine which bacterial species are associated with protective immune responses following BCG vaccination. Exploratory immunological analyses will also provide mechanistic insight into how the microbiota influence immune responses, and how local immune responses in the skin correlate with peripheral immune responses in the blood following BCG vaccination. Together, these data may provide evidence for the future development of microbiota-targeted therapies to improve vaccination efficacy or other immune outcomes.

### Data and sample collection

Participants will attend five study visits if allocated to the BCG-challenge arm (A), or seven study visits if allocated to the YFV-challenge arm (B), as illustrated in figure 1. Blood samples will be collected at each visit, and three stool samples will be collected throughout the study period (self-collected at home).

Samples and associated data will be retained by the study team for laboratory analysis of primary, secondary and exploratory outcomes of Antibiotics and Vaccine Immune Responses Study (AVIRS). Deidentified data will be stored on secure institutional repositories for at least 15 years. Participants may also provide optional consent at the beginning of the study to the use of samples and deidentified data for future ethically approved research.

#### Web-based questionnaires

Web-based questionnaires will be used to assess participant eligibility. Eligibility will be determined based on predefined inclusion and exclusion criteria, alongside clinical assessment and judgement. Following consent, participants will complete a baseline questionnaire capturing background demographic and medical information. Additional web-based questionnaires will be sent to participants following antibiotics or vaccination to record symptoms and adverse events (AEs)—with clinical follow-up by a dedicated study medical monitor for severe or ongoing symptoms as appropriate.

#### Blood sampling

Blood samples (30–50 mL) will be collected at each visit into sodium heparin tubes for RNA preservation, fixed blood preparation and PBMC isolation; sodium citrate tubes for plasma collection and clotting activator tubes for serum. Blood samples will be used for assessment of viraemia (YFV-challenge arm) and assessing vaccine responses (antibody and T-cell responses) in addition to exploratory systems immunology analyses.

#### Stool sampling

Stool samples will be self-collected by all participants at three timepoints:

1. 3–5 days prior to vaccination (before commencement of antibiotic treatment in participants randomised to the antibiotic group, or at the corresponding timepoint in participants not receiving antibiotics).
2. 0–1 day prior to vaccination (on the final day or day after antibiotic treatment in participants randomised to the antibiotic group, or at the corresponding timepoint in participants not receiving antibiotics).
3. 3- or 6 months post vaccination (ie, 0–1 days prior to the challenge vaccination).

#### Skin biopsy

For participants in the BCG challenge arm, a punch biopsy (4 mm) will be collected from the BCG challenge site at 2 weeks postchallenge by trained medical staff and stored for analysis.

### Sample size

#### BCG-challenge arm

In a previous study, prior BCG vaccination led to a sixfold reduction in bacterial load at the challenge site (intradermal) following secondary BCG challenge compared with placebo.^36^ Using these data (mean=3213, SD=2534), our power calculation (G*Power v3.1.9.7) estimates that n=38/group is sufficient to detect a twofold increase in bacterial load in antibiotic-treated individuals with 95% power (two-tailed Wilcoxon-Mann-Whitney, logistic distribution). Therefore, we will recruit n=42 per arm (total n=168 participants) to allow up to 10% loss to follow-up.

#### YFV-challenge arm

Arts *et al* previously demonstrated that YFV viraemia at 5 days following administration was significantly reduced in BCG vaccinated individuals compared with placebo vaccinated individuals.^21^ Given these data (combined mean=150, SD=248), our power calculation (G*Power v3.1.9.7) estimates that n=41/group is sufficient to detect a twofold difference in viraemia between antibiotic-treated and untreated individuals with 80% power (two-tailed Wilcoxon-Mann-Whitney, logistic distribution). We will recruit n=45/arm to allow 10% loss to follow-up.

### Statistical analysis

Primary and secondary endpoints will be analysed using linear regression models with adjustment for pre-specified baseline covariates, including the stratification variable (sex). Linear regression was chosen over non-parametric alternatives due to the flexibility to incorporate covariate adjustment and sufficient sample size for the central limit theorem to apply. Robust variance estimation will be used to allow for possible non-constant variance. For the primary outcomes, analyses will be performed on the subgroup randomised to BCG vaccination and results will be presented as differences in means (antibiotics vs no antibiotics) with 95% CIs. Within each challenge arm, analyses will follow the intention-to-treat principle, with participants analysed according to their allocated intervention irrespective of protocol adherence. Missing data will not be imputed. Analyses will therefore be restricted to available observed data, with the amount of missing data reported for each endpoint. All analyses of primary and secondary endpoints will follow a pre-specified statistical analysis plan, which will be finalised prior to unblinding.

Shotgun metagenomic sequencing data will be analysed using contemporary best-practice approaches, finalised in consultation with the study team and bioinformatics/statistical analysts prior to unblinding. Analyses will include sequence quality control, removal of host-derived reads, assessment of sequencing depth and sample quality, taxonomic profiling and characterisation of microbial functional potential. Additional exploratory analyses may include strain-level profiling, microbial diversity analyses, pathway or gene-family profiling, inference of microbial metabolic capacity and integration with other study data sets, including skin bacterial load or viraemia, immune response measures and RNA-seq data. Associations between microbiome-derived features and clinical or immunological outcomes will be assessed using appropriate statistical methods selected according to the distribution, sparsity and compositional nature of the data. These may include diversity-based comparisons, differential abundance or enrichment analyses, correlation analyses and multivariable models adjusted for relevant covariates. As these analyses are exploratory outcomes, findings will be interpreted as hypothesis-generating. Peripheral blood RNA-seq data will be analysed using established transcriptomic workflows, including sequence quality control, read alignment or quantification, normalisation, differential expression analysis and pathway- or gene set-level analyses. Integration of RNA-seq data with metagenomic and immunological data sets will be performed using appropriate exploratory statistical approaches. Spatial transcriptomics for punch biopsy samples will be performed using a 10X Xenium system (or an alternative spatial transcriptomics platform), a hybridisation-based system that assesses expression of hundreds of defined targets in a spatially-resolved manner across a tissue sample. Analysis techniques for spatial transcriptomics are still evolving, however, data analysis will be conducted by a dedicated specialist in spatial transcriptomics together with the South Australian Genomics Centre. Given the expense of this technique, analysis will be performed on tissue from participants defined as high and low responders (six participants per group) based on skin bacterial load and peripheral blood BCG-specific T-cell responses.

### Pilot study

To optimise laboratory techniques associated with the skin punch biopsies, a pilot study will be conducted in up to 10 additional participants. These participants will provide informed consent and undergo a single skin punch biopsy, with no other interventions. No additional personal information will be collected or retained beyond that required for this procedure. Participants will be recruited via institutional noticeboards and email communications. Following consent, participants will attend a single study visit for the punch biopsy procedure, which will be performed by medical staff. A follow-up visit ~7 days later will be arranged if required.

## Ethics and dissemination

### Ethics

The trial will be run in accordance with the ethical principles of the Declaration of Helsinki. The primary Human Research Ethics Committee (HREC) is the Central Adelaide Local Health Network HREC (Adelaide, South Australia) who approved this protocol (2023/HREC00066). Protocol amendments, where required, will be sought from the CALHN HREC and all approved documents made available to investigators on a secure online repository. The study sponsor is the South Australian Health and Medical Research Institute (SAHMRI). The principal investigators are responsible for study design, conduct, analysis and reporting of trial results. An independent data safety monitoring board (DSMB) of three clinical experts, independent of the study, will oversee trial conduct and safety. The DSMB will meet quarterly to review the progression of the study to maintain the integrity of the study and ensure the safety of the participants by reviewing the AEs. The DSMB charter is available in the online supplemental material.

### Risks

Participants will record potential antibiotic or vaccine-related AEs using an electronic questionnaire; with clinical follow-up by a dedicated study medical monitor for severe or ongoing symptoms as appropriate. Serious AEs will be reported to the DSMB, Human Research Ethics Committee and relevant regulatory authorities in accordance with Good Clinical Practice and local reporting requirements (see attached protocol in the online supplemental material for detailed Safety Monitoring and Reporting processes).

This study involves the use of oral vancomycin and neomycin. The AEs associated with these antibiotics are typically restricted to self-resolving GI symptoms, unless significant serum concentrations occur (eg, in renal impairment). Thus, participants with known chronic kidney disease or renal insufficiency are excluded from this study. The short 3 day antibiotic treatment regimen used in this protocol has been previously shown in other published studies to be well-tolerated and effective even in vulnerable populations (eg, cancer patients).^38^ To support participants who develop GI symptoms following antibiotic use, participants will be provided with a prescription for antinausea and antidiarrhoeal medications, and advice on their use. There is also a risk (as with any antibiotic treatment) that antibiotic use may increase the carriage of antibiotic resistance genes. This risk has been minimised by using a short 3 day antibiotic course, as antimicrobial resistance is more frequently associated with long term antibiotic use,^39 40^ and thus we do not anticipate that this treatment will significantly increase the risk of antibiotic-resistant infection. We will perform shotgun metagenomics sequencing on samples collected before and after antibiotics treatment to assess antibiotic resistance genes associated with antibiotic treatment within the study cohort.

This study involves administration of BCG and YFV, which both have a well-established safety profile in healthy individuals. The BCG vaccine is a licensed vaccine that is routinely administered to ~100 million individuals per year,^2^ and is associated with the formation of an injection site lesion that heals into a small flat scar. Individuals who receive two BCG vaccinations in this study may be at risk of a more immediate, accelerated local reaction following the second BCG vaccination.^41^ Previous data from the BRACE trial (trial registration: NCT04327206, ClinicalTrials.gov), found increased risk of local site reactions in BCG re-vaccinated participants compared with naïve participants vaccinated with BCG, including local pain (44% increase), erythema (9% increase), swelling (25% increase) and itch (8% increase).^41^ The YFV (attenuated yellow fever virus 17D strain) is associated with minor symptoms including mild, local reactions at the site of vaccination and myalgia. More severe symptoms, including YFV associated neurotropic or viscerotropic disease are rare (<1 in 100 000) and generally associated with immunocompromised individuals or the elderly, which are not eligible for this study. Live vaccines are contraindicated in pregnancy, due to potential risks to foetal development. Therefore, pregnant or breastfeeding individuals, or those planning to become pregnant, are not eligible for this study, and participants are offered a pregnancy self-test.

### Limitations

The study population in AVIRS consists of young, healthy, BCG-naïve adults in a low TB-endemic setting (metropolitan South Australia), which may limit generalisability of our findings to populations in which BCG vaccination is routinely administered, including infants and individuals in high TB-burden regions with prior mycobacterial exposure. In addition, the CHIM uses a dermal BCG challenge with attenuated *M. bovis*, which will not fully recapitulate the immunological mechanisms involved in protection against pulmonary *M. tuberculosis* infection in the lung, due to differences in route of exposure, tissue-specific immune responses, and pathogen biology.

Given that the antibiotic regimen used in this study is likely to cause notable GI side effects which may need to be discussed with clinical staff, and that microbiota depletion will be apparent to laboratory staff, participants and the clinical and laboratory team will not be blinded to antibiotic treatment group allocation. However, investigators responsible for data cleaning and analysis will be blinded to antibiotic allocation until data cleaning is complete before assessment of the primary outcome.

As the antibiotics used in this study are associated with notable GI side effects, variable adherence to the antibiotic regimen may occur and will be recorded via self-reported participant medication diaries and counting of remaining antibiotic tables. Variability in antibiotic adherence between participants may increase interindividual heterogeneity in the microbiota at the time of vaccination; however, this variability may strengthen exploratory correlative analyses between the extent of microbiota disruption and immune outcomes.

As a papule typically develops at the injection site approximately 2 weeks after BCG vaccination, it will be challenging to blind participants to their vaccine group allocation, even with a placebo. We have chosen objective primary outcomes to decrease the risk that awareness of allocation biases the trial results. Members of the research team conducting the analyses will be blinded to group allocation by the removal of all variables related to vaccination from the data set, until data cleaning is complete before assessment of the primary outcome.

### Perspective

This study has been designed to address key gaps in our understanding of how the gut microbiota influences vaccine-induced immunity in humans. Notably, both the immunogenicity and protective efficacy of the BCG vaccine are highly variable across geographic settings, a phenomenon that remains poorly explained. Differences in environmental microbial exposures, including the gut microbiota, are increasingly proposed as contributing factors. By experimentally depleting the gut microbiota prior to vaccination, this study will directly test whether microbiota composition contributes to this variability.

A major strength of this study is the use of CHIMs using live-attenuated organisms as a surrogate challenge. While these models use avirulent strains, they provide a controlled and ethical approach to assessing vaccine-induced protection in humans and allow direct quantification of microbial burden as an objective measure of vaccine efficacy. This represents a novel approach to dissecting the mechanisms underlying BCG-induced protection.

The study will be conducted in young, healthy, BCG-naïve adults, which minimises confounding effects of age, comorbidities and pre-existing mycobacterial immunity, and allows assessment of primary vaccine responses without the influence of prior exposure. Antibiotic-induced perturbation of the gut microbiota is expected to result in heterogeneous patterns of microbiota disruption and recovery. This study design is therefore expected to capture a biologically diverse cohort, reflecting real-world variability in microbiota composition. Recolonisation following antibiotic exposure is also known to be variable between individuals, which may facilitate exploration of associations between specific microbial taxa and immune outcomes. Similarly, variation in adherence to the antibiotic regimen may lead to differing degrees of microbiota depletion, which, while a potential limitation, may also increase the ability to detect relationships between microbiota disruption and immune responses.

If depletion of the gut microbiota is shown to alter vaccine-induced immunity, these findings would have important implications for understanding variability in vaccine responses and for the development of microbiota-targeted strategies to enhance vaccine efficacy, with particular relevance for populations with impaired vaccine responses. Additionally, the use of a challenge model together with longitudinal sampling and systems immunology analyses will enable detailed characterisation of immune responses to BCG and may help to identify correlates of BCG-induced protection against mycobacterial or other infections.

Ultimately, this study has the potential to fundamentally advance our understanding of the interaction between the gut microbiota and BCG-induced immunity and to inform the development of next-generation strategies to enhance vaccine effectiveness against TB and other infectious diseases.

### Dissemination

Results will be published in a peer-reviewed journal, which will be made available to all study participants. Deidentified data accompanying the publication (including metagenomics, transcriptomics, epigenomics and other related immunological data sets) will be deposited in appropriate international repositories, in line with the editorial policies of the publisher.

## Supplementary material

10.1136/bmjopen-2026-126924 online supplemental file 1

10.1136/bmjopen-2026-126924 online supplemental file 2

10.1136/bmjopen-2026-126924 online supplemental file 3

10.1136/bmjopen-2026-126924 online supplemental file 4

10.1136/bmjopen-2026-126924 online supplemental file 5

## References

[1] Hofer SJ, Rapp S, Klenerman P (2025). Understanding and improving vaccine efficacy in older adults. Nat Aging.

[2] Dockrell HM, Butkeviciute E (2022). Can what have we learnt about BCG vaccination in the last 20 years help us to design a better tuberculosis vaccine?. Vaccine.

[3] Aaby P, Benn CS, Flanagan KL (2020). The non-specific and sex-differential effects of vaccines. Nat Rev Immunol.

[4] Lynn DJ, Benson SC, Lynn MA (2022). Modulation of immune responses to vaccination by the microbiota: implications and potential mechanisms. Nat Rev Immunol.

[5] Rossouw C, Ryan FJ, Lynn DJ (2025). The role of the gut microbiota in regulating responses to vaccination: current knowledge and future directions. FEBS J.

[6] Ardura-Garcia C, Curtis N, Zimmermann P (2024). Systematic review of the impact of intestinal microbiota on vaccine responses. NPJ Vaccines.

[7] Lynn DJ, Pulendran B (2018). The potential of the microbiota to influence vaccine responses. J Leukoc Biol.

[8] Lynn MA, Tumes DJ, Choo JM (2018). Early-Life Antibiotic-Driven Dysbiosis Leads to Dysregulated Vaccine Immune Responses in Mice. Cell Host Microbe.

[9] Woo PC, Tsoi HW, Wong LP (1999). Antibiotics modulate vaccine-induced humoral immune response. Clin Diagn Lab Immunol.

[10] Fiege JK, Block KE, Pierson MJ (2021). Mice with diverse microbial exposure histories as a model for preclinical vaccine testing. Cell Host Microbe.

[11] Ryan FJ, Clarke M, Lynn MA (2025). Bifidobacteria support optimal infant vaccine responses. Nature.

[12] Hagan T, Cortese M, Rouphael N (2019). Antibiotics-Driven Gut Microbiome Perturbation Alters Immunity to Vaccines in Humans. Cell.

[13] Feng Y, de Jong SE, Oliveira A (2025). Antibiotic-induced gut microbiome perturbation alters the immune responses to the rabies vaccine. Cell Host Microbe.

[14] World Health Organization (2025). Global tuberculosis report 2025.

[15] Pelzer PT, Stuck L, Martinez L (2025). Effectiveness of the primary Bacillus Calmette-Guérin vaccine against the risk of Mycobacterium tuberculosis infection and tuberculosis disease: a meta-analysis of individual participant data. Lancet Microbe.

[16] Mangtani P, Abubakar I, Ariti C (2014). Protection by BCG Vaccine Against Tuberculosis: A Systematic Review of Randomized Controlled Trials. Clin Infect Dis.

[17] van Dorst MMAR, Pyuza JJ, Nkurunungi G (2024). Immunological factors linked to geographical variation in vaccine responses. Nat Rev Immunol.

[18] Dockrell HM, Smith SG, Lalor MK (2012). Variability between countries in cytokine responses to BCG vaccination: what impact might this have on protection?. Expert Rev Vaccines.

[19] Nadeem S, Maurya SK, Das DK (2020). Gut Dysbiosis Thwarts the Efficacy of Vaccine Against *Mycobacterium tuberculosis.*. Front Immunol.

[20] Bukula L, Chengalroyen MD, Omollo C (2025). Factors influencing the efficacy of Bacille Calmette-Guérin (BCG) vaccine. *The Microbe*.

[21] Arts RJW, Moorlag SJCFM, Novakovic B (2018). BCG Vaccination Protects against Experimental Viral Infection in Humans through the Induction of Cytokines Associated with Trained Immunity. Cell Host Microbe.

[22] de Castro MJ, Pardo-Seco J, Martinón-Torres F (2015). Nonspecific (Heterologous) Protection of Neonatal BCG Vaccination Against Hospitalization Due to Respiratory Infection and Sepsis. Clin Infect Dis.

[23] Pittet LF, Casalaz D, Donath S (2026). Effect of neonatal BCG vaccination on oral herpes in early childhood: A nested study within a randomised controlled trial. Vaccine.

[24] Higgins JPT, Soares-Weiser K, López-López JA (2016). Association of BCG, DTP, and measles containing vaccines with childhood mortality: systematic review. BMJ.

[25] Aaby P, Roth A, Ravn H (2011). Randomized Trial of BCG Vaccination at Birth to Low-Birth-Weight Children: Beneficial Nonspecific Effects in the Neonatal Period?. J Infect Dis.

[26] Biering-Sørensen S, Aaby P, Lund N (2017). Early BCG-Denmark and Neonatal Mortality Among Infants Weighing <2500 g: A Randomized Controlled Trial. Clin Infect Dis.

[27] Datau EA, Sultana A, Mandang VVV (2011). The efficacy of Bacillus Calmette-Guerin vaccinations for the prevention of acute upper respiratory tract infection in the elderly. Acta Med Indones.

[28] Pittet LF, Moore CL, McDonald E (2023). Bacillus Calmette-Guérin vaccination for protection against recurrent herpes labialis: a nested randomised controlled trial. EClinicalMedicine.

[29] Pittet LF, Messina NL, Orsini F (2023). Randomized Trial of BCG Vaccine to Protect against Covid-19 in Health Care Workers. N Engl J Med.

[30] Inauen J, LaBroome S, Maldari A (2025). Efficacy of Bacille Calmette-Guérin Against COVID-19 Hospitalisation: A Meta-Analysis and Systematic Review of Randomised Control Trials. Vaccines (Basel).

[31] Moorlag SJCFM, Taks E, Ten Doesschate T (2022). Efficacy of BCG Vaccination Against Respiratory Tract Infections in Older Adults During the Coronavirus Disease 2019 Pandemic. Clin Infect Dis.

[32] Pittet LF, Messina NL, McDonald E (2024). Bacille Calmette-Guérin vaccination to prevent febrile and respiratory illness in adults (BRACE): secondary outcomes of a randomised controlled phase 3 trial. EClinicalMedicine.

[33] Cirovic B, de Bree LCJ, Groh L (2020). BCG Vaccination in Humans Elicits Trained Immunity via the Hematopoietic Progenitor Compartment. Cell Host Microbe.

[34] Moorlag SJCFM, Rodriguez-Rosales YA, Gillard J (2020). BCG Vaccination Induces Long-Term Functional Reprogramming of Human Neutrophils. Cell Rep.

[35] Kaufmann E, Sanz J, Dunn JL (2018). BCG Educates Hematopoietic Stem Cells to Generate Protective Innate Immunity against Tuberculosis. Cell.

[36] Minhinnick A, Harris S, Wilkie M (2016). Optimization of a Human Bacille Calmette-Guérin Challenge Model: A Tool to Evaluate Antimycobacterial Immunity. J Infect Dis.

[37] Tanes C, Bittinger K, Gao Y (2021). Role of dietary fiber in the recovery of the human gut microbiome and its metabolome. Cell Host Microbe.

[38] Baruch EN, Youngster I, Ben-Betzalel G (2021). Fecal microbiota transplant promotes response in immunotherapy-refractory melanoma patients. Science.

[39] Spellberg B, Rice LB (2019). Duration of Antibiotic Therapy: Shorter Is Better. Ann Intern Med.

[40] Costelloe C, Metcalfe C, Lovering A (2010). Effect of antibiotic prescribing in primary care on antimicrobial resistance in individual patients: systematic review and meta-analysis. BMJ.

[41] Villanueva P, Crawford NW, Garcia Croda M (2023). Safety of BCG vaccination and revaccination in healthcare workers. Hum Vaccin Immunother.

