# Supplementary material for: A randomised, placebo-controlled study to assess whether antibiotic-mediated disruption of the gut microbiota impairs BCG-induced specific and non-specific immunity in young, healthy adults: protocol for the Antibiotics and Vaccine Immune Responses Study (AVIRS)
Source: BMJ Open. 2026 Oct 14;16(10):e126924. doi: 10.1136/bmjopen-2026-126924 (PMC13691821; doi:10.1136/bmjopen-2026-126924)
Supplement: Supplementary file 1 [file bmjopen-16-10-s003.pdf]

TITLE: A human experimental medicine study to assess whether the gut microbiota regulates specific and non-specific immune responses to vaccination

SHORT TITLE: Antibiotics and Vaccine Immune Responses Study (AVIRS)

PROTOCOL NUMBER: 2023/HREC00066

COORDINATING PRINCIPAL INVESTIGATOR: Prof David Lynn

ROYAL ADELAIDE HOSPITAL SITE PRINCIPAL INVESTIGATOR: Dr Simone Barry

VERSION: 8.0

SUPPORTED BY NHMRC

DATE OF PROTOCOL: 13 August 2025

Clinicaltrials.gov NUMBER: NCT06148025

## PRINCIPAL INVESTIGATOR SIGNATURE PAGE

I have reviewed and approve this protocol. My signature verifies that this clinical study will be carried out in accordance with the protocol and all relevant local laws and regulations. This comprises, but is not limited to, the International Conference on Harmonisation Guideline for Good Clinical Practice (ICH GCP), the ethical principles founded from the Declaration of Helsinki, the NHMRC National Statement on Ethical Conduct in Research involving Humans and relevant privacy laws.

| | |
|---|---|
| ______________________________ | _________________________ |
| COORDINATING PRINCIPAL INVESTIGATOR SIGNATURE | DATE (DD/MMM/YYYY) |

______________________________
COORDINATING PRINCIPAL INVESTIGATOR NAME *(print)*

| | |
|---|---|
| ______________________________ | _________________________ |
| SITE PRINCIPAL INVESTIGATOR SIGNATURE | DATE (DD/MMM/YYYY) |

Dr Simone Barry
______________________________
SITE PRINCIPAL INVESTIGATOR NAME *(print)*

# CONTACTS

| | | |
|---|---|---|
| Coordinating Principal Investigator | **Prof. David Lynn**<br>Director, Computational & Systems Biology Program, Precision Medicine Theme,<br>South Australian Health and Medical Research Institute | **Responsibilities**<br>CPI Lynn will have overall responsibility for the study. This includes securing funding and implementing the scientific design. The CPI will also be responsible for overseeing the processing, analysis and storage of the samples |
| Site Principal Investigator | **Dr. Simone Barry**<br>Respiratory and Sleep Medicine Physician<br>Department of Respiratory Medicine | Site PI Barry is responsible for the clinical oversight of the study, including the administration of the BCG vaccination and the monitoring and reporting of safety events to the relevant authorities |
| Associate Investigators | **Dr. James Geake**<br>Department of Respiratory Medicine<br>Royal Adelaide Hospital | AI Geake is responsible for the clinical duties including administering the punch biopsy |
| | **Dr. Alice Sawka**<br>Department of Respiratory Medicine<br>Royal Adelaide Hospital | AI Sawka is responsible for the clinical duties including administering the punch biopsy |
| | **Dr. Joseph Inauen**<br>Department of Respiratory and Sleep Services<br>Flinders Medical Centre | AI Inauen is responsible for the clinical duties including administering the punch biopsy |
| | **Dr Jelena Solujic**<br>Department of Respiratory Medicine<br>Royal Adelaide Hospital | AI Solujic is the medical monitor for the study and will be responsible for reporting safety events to the PIs and relevant authorities |

| | | |
|---|---|---|
| | **Dr. Natalie Stevens**<br>Postdoctoral Researcher<br>Precision Medicine<br>South Australian Health and Medical Research Institute | AI Stevens is responsible for conducting the processing, analysis and storage of the samples |
| | **Prof, Warwick Britton AO**<br>Head of Tuberculosis Research Program<br>Centenary Institute & University of Sydney | AI Britton is an expert in TB research and will provide insight on study design and interpretation of results |
| | **Prof. Carl Feng**<br>Professor of Medicine (Infectious Diseases and Immunology) | AI Feng is an expert in TB research and will provide advice on the immunological assays to be used in the analysis of samples collected in the study |
| | **Prof. Tobi Kollmann**<br>Head, Systems Vaccinology<br>Telethon Kids Institute, Perth Children's Hospital | AI Kollmann has expertise in conducting similar BCG challenge studies and is providing advice on study data design and data interpretation |
| Study Coordinator (SC) | **Mrs Rochelle Botten**<br>Project Manager<br>Clinical Trials Platform<br>South Australian Health and Medical Research Institute | The SC is responsible for the management of the participants. The SC is also responsible for collecting and deidentifying the data |
| Study Statistician | **Dr. Lisa Yelland**<br>SAHMRI Biostatistics Unit<br>South Australian Health and Medical Research Institute | The Statistician is responsible for the randomisation of participants and analysis of the primary outcome data |
| Safety and Data Management Committee | *Names and affiliations have been anonymised for publication* | This committee will be responsible for overseeing the safety of participants and study conduct and will provide advice to the PIs. See AVIRS DSMB charter |

| | | |
|---|---|---|
| Manager of Laboratory Studies | **Dr. Miriam Lynn**<br>Postdoctoral Research Fellow<br>Precision Medicine<br>South Australian Health and Medical Research Institute | Dr Lynn will be responsible for managing samples collected in the study and will assist Al Stevens in the processing, analysis and storage of these samples |

# PROTOCOL DEVELOPMENT HISTORY

| Version | Date | Author | Reason(s) |
|---|---|---|---|
| 1.0 | 20/JAN/2021 | Rochelle Botten | Development |
| 1.1 | 14/MAR/2023 | Rochelle Botten | Submission to CALHN HREC |
| 1.2 | 24/MAR/2023 | Rochelle Botten | Updated to respond to pre-review from the CALHN HREC Office |
| 2.0 | 10/MAY/2023 | Rochelle Botten | Revised after initial review from the CALHN HREC Office |
| 3.0 | 17/AUG/2023 | Rochelle Botten | Amendment submitted to HREC to include Yellow Fever vaccine arm of study |
| 4.0 | 14/FEB/2024 | Rochelle Botten | Amendment submitted to HREC with administrative changes and changes to participant payment |
| 4.1 | 10/APR/2024 | Rochelle Botten | Amendment submitted to HREC with administrative changes and Pilot Participant communication |
| 5.0 | 09/MAY/2024 | Rochelle Botten | Amendment submitted to HREC with administrative changes and addition of Metoclopramide and Loperamide and Medicare Number |
| 6.0 | 10/FEB/2024 | Rochelle Botten | Amendment submitted to HREC with update to Protocol and the vaccination SOPs as well as the addition of a participant survey after having the Punch Biopsy |
| 7.0 | 18/JUN/2025 | Rochelle Botten | Amendment submitted to HREC with update to Protocol and phlebotomy SOPs as well as expanded advertising and follow up antibiotic survey |
| 8.0 | 13/AUG/2025 | Rochelle Botten | Amendment submitted to HREC with administrative changes for advertising |

# Contents


PRINCIPAL INVESTIGATOR SIGNATURE PAGE ........ 2

CONTACTS ........ 3

PROTOCOL DEVELOPMENT HISTORY ........ 6

Contents ........ 7

1 PROTOCOL SYNOPSIS ........ 9

2 LIST OF ABBREVIATIONS ........ 14

3 INTRODUCTION: BACKGROUND AND SCIENTIFIC RATIONALE ........ 15

3.1 Scientific rationale and aim ........ 15

3.2 Background ........ 15

3.3 Risk assessment ........ 17

3.3.1 Known potential risks ........ 17

3.3.2 Assessment of potential risks ........ 21

4 STUDY OBJECTIVES AND OUTCOMES ........ 22

4.1 Primary objective ........ 22

4.2 Secondary objective ........ 22

4.3 Outcomes ........ 23

5 STUDY DESIGN ........ 23

5.1 Overall design ........ 23

5.2 Justification for the doses ........ 25

5.3 Study population ........ 25

5.3.1 Eligibility criteria ........ 25

5.3.2 Exclusion criteria ........ 25

5.4 Screen failures ........ 26

5.5 Recruitment and consent ........ 26

5.6 Enrolment visit ........ 29

6 BCG VACCINATION AND CHALLENGE VACCINATION ........ 29

6.1 Treatment arms ........ 29

6.2 Study interventions ........ 29

6.2.1 Antibiotics ........ 29

6.2.2 BCG vaccine ........ 30

6.2.3 Placebo to match BCG vaccine ........ 30

6.2.4 Yellow Fever vaccine ........ 30

6.3 Dosage ........ 30

6.4 Dose modification ........ 30

6.5 Storage and dispensing of vaccines ..... 30
6.6 Preparation ..... 32
6.7 Administration of study drug ..... 33
6.7.1 Antibiotics ..... 33
6.7.2 BCG and placebo injection ..... 34
6.7.3 Yellow Fever vaccine ..... 36
6.8 Product accountability ..... 37
6.9 Excluded medications and treatments ..... 37
7 RANDOMISATION AND BLINDING ..... 37
8 STUDY VISITS AND PROCEDURES ..... 38
8.1 Study timeline ..... 38
8.2 Schedule of assessments ..... 40
8.3 Description of procedures ..... 40
9 SAFETY MONITORING AND REPORTING ..... 42
9.1 Definitions ..... 42
9.2 Capturing adverse event information ..... 43
9.3 Documentation of AEs ..... 44
9.4 Reporting of safety events ..... 47
10 DATA AND INFORMATION MANAGEMENT ..... 47
10.1 Overview ..... 47
10.2 Data management ..... 47
10.3 Sample management ..... 48
11 STATISTICAL METHODS ..... 48
11.1 Sample size estimation ..... 48
11.2 Methods of analysis ..... 48
12 ETHICS AND DISSEMINATION ..... 49
12.1 Research ethics approval and local governance authorisation ..... 49
12.2 Benefits and risks ..... 50
12.3 Amendments to the protocol ..... 50
12.4 Protocol deviations ..... 50
12.5 Declaration of interests ..... 50
12.6 Dissemination plan ..... 50
13 PARTICIPANT REIMBURSEMENT ..... 50
14 REFERENCES ..... 52
15 ATTACHMENTS ..... 54

# 1 PROTOCOL SYNOPSIS

| | |
|---|---|
| Title | A human experimental medicine study to assess whether the gut microbiota regulates specific and non-specific immune responses to vaccination |
| Short Title | Antibiotics and Vaccine Immune Responses Study (AVIRS) |
| Study description | The study is divided into two arms.<br><br>The first arm (BCG re-challenge arm) is an experimental medicine study in 168 healthy participants to determine if depletion of the gut microbiota leads to impaired BCG-induced protection against a subsequent *Mycobacterium bovis* BCG intradermal challenge (This arm was approved by HREC 16th May 2023; 2023/HRE00066). An additional 10 participants will be recruited to this arm and will receive a punch biopsy only (no vaccinations, antibiotics or other interventions) to optimise the methodology for spatial transcriptomics, a very new technique that allows the measurement of which genes are turned on and off in different parts of the skin (see section 8.3).<br><br>The second arm (Yellow Fever vaccine arm) has a very similar experimental design to the first arm but will determine if depletion of the gut microbiota leads to impaired BCG-induced protection against other infections. To assess this, participants in this arm (n=180) will be re-challenged after 3 months with a live attenuated viral vaccine, the Yellow Fever (YF) vaccine, which induces a mild viremia.<br><br>In both arms, participants will initially be randomised to receive a 3 day course of antibiotics or none (comparator group). The two groups in each arm will be randomised again to receive either BCG vaccine or 0.9% NaCl placebo injection in the left arm.<br><br>**BCG re-challenge arm (Arm 1):** Six months following randomisation, all participants in Arm 1 will receive a BCG vaccine challenge in the right arm. A punch skin biopsy will be taken of this challenge site 2 weeks after the challenge to assess *M. bovis* BCG bacterial load in the skin.<br><br>**YF vaccine arm (Arm 2):** Three months following randomisation, all participants in Arm 2 will receive a YF vaccine challenge in the right arm. Blood samples will be collected from YF vaccinated participants at day 3, 5 and 7 following YF vaccine challenge to quantify YF viral load in blood.<br><br>All participants will complete a survey after antibiotics and after each vaccination to assess any side effects. Blood samples will be collected at randomisation, before each vaccination, 2 weeks after each BCG |

| | |
|---|---|
| | vaccination and in the YF vaccine arm at day 3, 5 and 7 following YF vaccination. Stool samples will be collected prior to randomisation, and prior to each vaccination. |
| Rationale | For reasons that are poorly understood, the immunity induced by many vaccines, including the Bacillus Calmette–Guérin (BCG) vaccine, a live-attenuated vaccine against tuberculosis that is administered to ~100 million infants a year, are highly variable between different populations and immunity is frequently suboptimal in the very populations who are at most risk from infection. Studies in animal models, including our own, have convincingly demonstrated that the gut microbiota plays a critical role in regulating antigen-specific immune responses to vaccination. The evidence in human studies, however, is far less clear. A number of small clinical cohort studies have identified associations between the relative abundance of specific bacteria in the gut microbiota and vaccine immunogenicity, however, a causal relationship has not been established. Conclusive evidence in human studies is needed to support the clinical translation of microbiota-targeted interventions to boost vaccine efficacy.<br><br>Furthermore, a substantive body of evidence now suggests that some vaccines, such as the BCG vaccine, can also provide non-specific protection against other unrelated infections. For example, BCG was shown to provide non-specific protection against a Yellow Fever (YF) vaccine challenge in healthy human participants. While strong preclinical evidence indicates that the gut microbiota plays a critical role in regulating antigen-specific immune responses to vaccination, whether the gut microbiota can influence the non-specific effects of vaccines is almost completely unexplored.<br><br>Here, we will use a highly innovative experimental medicine study to conclusively assess whether depletion of the human gut microbiota prior to BCG immunisation leads to impaired mycobacterial-specific immunity (Arm 1) or altered non-specific immune responses (Arm 2). To do this, a cohort of young, healthy, adult volunteers will be untreated (control) or will receive a course of oral antibiotics prior to immunisation with BCG. The impact of depleting the gut microbiota on the efficacy of BCG against a secondary challenge with *Mycobacterium bovis* BCG (a human model of mycobacterial-specific immunity) will be assessed in Arm 1, while in Arm 2 participants will be challenged with a YF vaccine (a live viral vaccine). |
| Objectives and endpoints | **Primary Objectives:**<br>• Arm 1: Determine if depletion of the gut microbiota leads to impaired BCG-induced protection against a subsequent *M. bovis* BCG intradermal challenge in a controlled human experimental BCG rechallenge model.<br>• Arm 2: Determine if depletion of the gut microbiota, at the time of BCG immunisation, significantly reduces viremia following challenge with a live attenuated viral vaccine (YF vaccine). |

| | |
|---|---|
| | **Secondary Objectives:**<br>• Employ a systems immunology approach to determine how BCG mediates anti-mycobacterial immunity in the skin and alters immune responses following YF vaccination.<br>• To determine whether these responses are influenced by the gut microbiota.<br><br>**Primary Endpoints:**<br>• **Arm 1:** Mycobacterial load (Colony Forming Units (CFU)) in the skin biopsy site in BCG-vaccinated participants not exposed to antibiotics (BCG-No ABX) compared to BCG-vaccinated participants that were exposed to antibiotics (BCG-ABX).<br>• **Arm 2:** Yellow Fever viremia (viral copies/ml blood) at D3-7 post YF vaccination in BCG-vaccinated participants not exposed to antibiotics (BCG-No ABX) compared to BCG-vaccinated participants that were exposed to antibiotics (BCG-ABX).<br><br>**Secondary Endpoints:**<br>Arm 1<br>• Mycobacterial load (CFU) in the skin biopsy site in BCG-vaccinated participants compared to placebo-vaccinated participants.<br>• Anti-mycobacterial T cell responses (IFNγ production and activation induced marker expression), antibody responses, and other immune parameters in peripheral blood collected from BCG-vaccinated participants not exposed to antibiotics (BCG-No ABX) compared to BCG-vaccinated participants that were exposed to antibiotics (BCG-ABX).<br><br>Arm 2<br>• Viraemia (viral copies/ml blood) at D3-7 post YF vaccination in BCG-vaccinated participants compared to placebo-vaccinated participants.<br>• Comparison of innate immune responses (e.g. PBMC cytokine responses following *in vitro* stimulation, expression of activation markers on immune cells, frequency of immune cell populations in peripheral blood) at baseline (prior to BCG vaccination), at 3 months (prior to YF vaccination) and following YF vaccination in all groups (i.e. BCG/Placebo with and without prior antibiotic exposure).<br><br>**Exploratory Endpoints:**<br>• Longitudinal analysis of blood samples through the study: immune cell frequencies and activation marker expression, molecular analysis of proteins, lipids and bacterial metabolites.<br>• Blood transcriptomics to measure gene expression (i.e. which genes are turned on or off in blood). |

| | |
|---|---|
| | • Bacterial load and microbial composition of the stool microbiota at D0 and 3 or 6 months compared to D-3 in all participants.<br>• Correlative analysis of metagenomic, transcriptomic, immunological and epigenomic datasets.<br>• Arm 1 only: Standard histology and immunohistochemistry of the BCG rechallenge site in BCG-vaccinated vs non vaccinated participants.<br>• Arm 1 only: Spatial transcriptomics of the BCG rechallenge site in high vs. low responders (as assessed based on *M. bovis* BCG bacterial load in the biopsy site).<br>• Arm 2 only: Comparison of genome-wide chromatin accessibility (i.e. which parts of the genome are “open” or “closed”) in peripheral blood monocytes, neutrophils and NK cells across all groups. |
| Number of Participants | 178 participants in the BCG cohort<br>180 participants in the YF vaccine cohort |
| Recruiting Period | 18 months |
| Duration of Participant involvement | 7 months for the BCG rechallenge arm<br>4 months for the YF vaccine arm |
| **Key** Inclusion Criteria | 1. BCG naive<br>2. Aged 18-35 years old<br>3. No other vaccinations one month prior to beginning the study<br>4. YF and YF vaccine naïve (for the YF vaccine arm)<br>**Refer to section 5.3.1 for a comprehensive list of inclusion criteria** |
| **Key** Exclusion Criteria | 1. Received or likely to have received BCG or YF vaccination previously<br>2. Has latent tuberculosis<br>3. Unwilling to take the course of antibiotics or undergo a punch biopsy<br>4. Pregnant or immunocompromised<br>5. Any medical history that would contra-indicate a live vaccine or antibiotic treatment (e.g. chronic renal disease, HIV infection, cancer, autoimmune disease, taking steroids, other immune-suppressants, chemotherapy, thymus disease)<br>6. Tattoo obscuring BCG vaccination site(s)<br>**Refer to section 5.3.2 for comprehensive list of exclusion criteria** |
| Interventions | Each participant will be randomised prior to the first BCG/placebo vaccination to receive either:<br>• Antibiotics (500mg vancomycin and 1000mg neomycin tablets every 6 hrs for 3 days) or<br>• No antibiotics (comparator group) |

| | |
|---|---|
| | Each participant will then be randomised to receive one of two vaccinations:<br>• BCG vaccination: BCG Denmark, 0.1 mL injected intradermal over the distal insertion of the deltoid muscle onto the humerus (left arm) or<br>• Placebo vaccination: 0.1 ml of 0.9% NaCl injected intradermal over the distal insertion of the deltoid muscle onto the humerus (saline)<br><br>Every participant will receive a 2nd vaccination in the right arm (either BCG or YF vaccine), 6 (BCG re-challenge arm) or 3 (YF vaccine arm) months later. |
| Statistical analyses | Primary Endpoint:<br>**Arm 1:** Bacterial load (CFU) at the biopsy challenge site will be compared between groups (vaccinated ABX vs vaccinated no ABX) via a linear regression model.<br>**Arm 2:** Viremia (viral copies/mL blood) will be compared between groups (vaccinated ABX vs vaccinated no ABX) via a linear regression model.<br>Analysis of other endpoints and exploratory analyses are detailed in the 'Statistical Methods' section. |
| Safety Assessments | Safety will be assessed through the analysis of the reported incidence of treatment-emergent adverse events (AEs) and serious adverse events (SAEs) |

# 2 LIST OF ABBREVIATIONS

| | |
|---|---|
| ABX | Antibiotics |
| AE | Adverse Event |
| BCG | Bacillus Calmette- Guérin vaccine |
| CALHN | Central Adelaide Local Health Network |
| CFU | Colony Forming Units |
| CHIM | Controlled human infection model |
| CRF | Case Report Form |
| DSMB | Data Safety Monitoring Board |
| GCP | Good Clinical Practice |
| GI | Gastrointestinal |
| HIC | High-Income Country |
| HREC | Human Research Ethics Committee |
| ICH | International Conference on Harmonisation |
| IGRA | Interferon Gamma Release Assay |
| IP | Investigational Product |
| LMIC | Low- and Middle-Income Country |
| LTBI | Latent Tuberculosis Infection |
| M. bovis | Mycobacterium bovis |
| Mtb | M. tuberculosis |
| NHMRC | National Health and Medical Research Council |
| NSEs | Non-specific effects |
| PI | Principal Investigator |
| PICF | Participant Information and Consent Form |
| RAH | Royal Adelaide Hospital |
| RCT | Randomised controlled trial |
| SAE | Serious Adverse Event |
| SAHMRI | South Australian Health and Medical Research Institute |
| SOP | Standard Operating Procedure |
| SSI | Significant Safety Issues |
| SUSAR | Suspected Unexpected Serious Adverse Reaction |
| TB | Tuberculosis |
| TNF | Tumour Necrosis Factor |
| USM | Urgent Safety Measure |
| WOCBP | Women Of Child Bearing Potential |
| YF | Yellow Fever |
| YFV | Yellow Fever Vaccine |

# 3 INTRODUCTION: BACKGROUND AND SCIENTIFIC RATIONALE

## 3.1 Scientific rationale and aim

Vaccines provide specific protection against the diseases they target by inducing antigen-specific immunity. For reasons that are poorly understood, immune responses induced by many vaccines, are highly variable between different populations and are frequently suboptimal in the very populations who are at most risk from infectious disease[1]. Immune responses to the widely-used Bacillus Calmette–Guérin (BCG) vaccine (a live-attenuated vaccine against tuberculosis containing *Mycobacterium bovis*, the causative agent of tuberculosis in cattle) are particularly enigmatic, and there are no established immunological correlates of protection for BCG.[2]

Studies in animal models, including our own[3], have convincingly demonstrated that the gut microbiota plays a critical role in regulating antigen-specific immune responses to vaccination. The evidence in human studies, however, is far less clear. A number of small clinical cohort studies have identified associations between the relative abundance of specific bacteria in the gut microbiota and vaccine immunogenicity[1], however, a causal relationship has not been established and most associations have not been replicated in other cohorts. Conclusive evidence in human studies is needed to support the clinical translation of microbiota-targeted interventions to boost vaccine efficacy.

A substantiative body of evidence indicated that some vaccines, including BCG, can provide non-specific protection against unrelated infections[4]. However, whether or not the gut microbiota has a role in these non-specific effects is almost completely unexplored.

This NHMRC-funded study will use two highly innovative experimental controlled human infection models (CHIM) to conclusively assess whether depletion of the human gut microbiota prior to BCG immunisation leads to a) impaired mycobacterial-specific immunity or b) impaired non-specific immunity. To do this, a cohort of young, healthy, adult volunteers will be untreated (control) or will receive a course of oral antibiotics prior to immunisation with BCG. The impact of depleting the gut microbiota on the efficacy of BCG against a secondary challenge with *Mycobacterium bovis* BCG (a human model of mycobacterial-specific immunity) or against a live-attenuated virus (a human model of non-specific immunity) will be assessed.

**Aim 1:** Determine if depletion of the gut microbiota leads to impaired BCG-induced protection against mycobacterial infection in a controlled human experimental BCG rechallenge model.

**Aim 2:** Determine if depletion of the gut microbiota leads to impaired BCG-induced protection against other infections in a controlled human experimental attenuated virus infection model.

**Aim 3:** Employ a systems immunology approach to determine how BCG mediates antigen-specific and non-specific immunity and determine whether these responses are influenced by the gut microbiota.

## 3.2 Background

Global immunisation programmes have led to the eradication or near eradication of diseases such as smallpox and polio, prevent million deaths/year, markedly reduce the severity of breakthrough infections[5], and significantly reduce antibiotic usage. For example, the pneumococcal and oral rotavirus vaccines prevent ~23.8M and 13.6M episodes of antibiotic-treated illness, respectively, among children <5yrs in low- and middle-income countries (LMICs) each year[6]. For reasons that are poorly understood, however, the immunogenicity of many vaccines is highly variable between individuals (up to 100-fold for some vaccines) and is frequently impaired in the very populations at most risk from infectious disease including infants, the elderly, and those living in LMICs. Rotavirus vaccine efficacy, for example, is 94% in high-income countries (HICs), compared with 44% in LMICs[7].

In a recent Nature Reviews Immunology article[1], similarly blunted immunogenicity has been reported in LMICs for many other vaccines, including BCG. These data suggest that specific host factors that vary between LMICs and HICs, and at the extremes of life, strongly influence the quality and durability of the immune response to vaccination. Increasing evidence points to the gut microbiota — which is highly variable between individuals, over the course of life[8,9] and between different populations around the world[10] — as a crucial and targetable factor that regulates immune responses to vaccination[1].

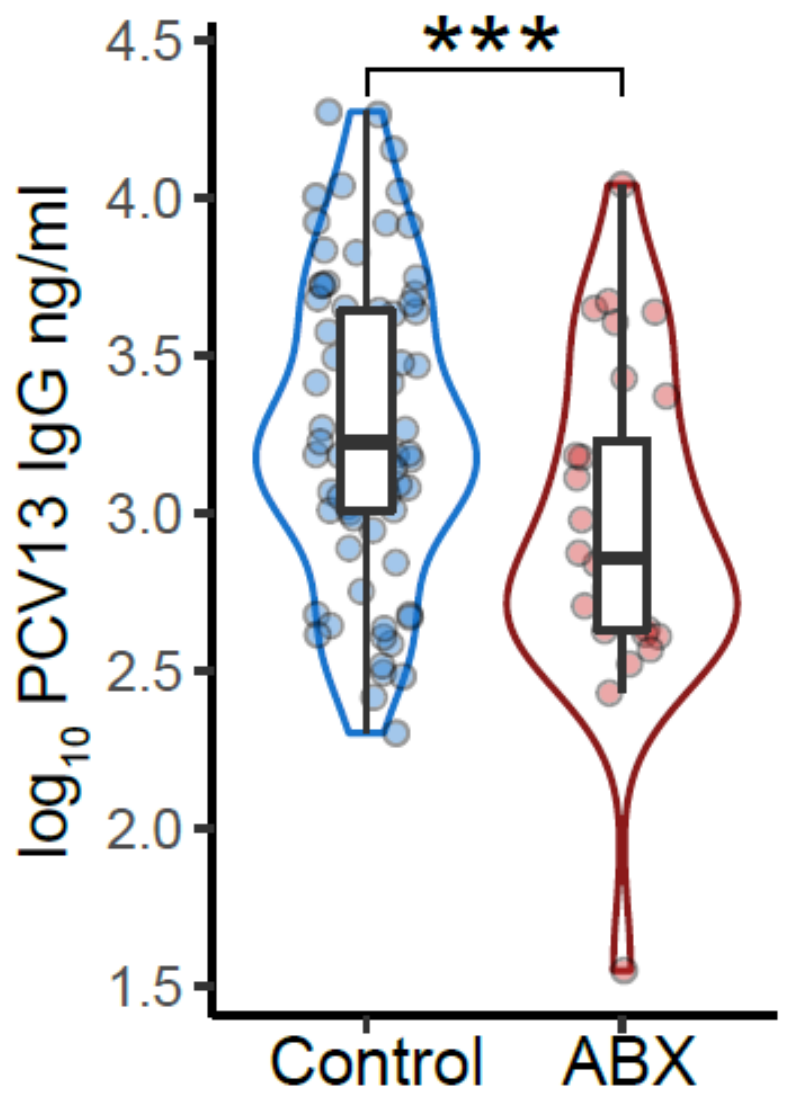


**Figure 1.** Infants exposed to antibiotics in the neonatal period have significantly reduced PCV13-specific antibody titres at 7 months of age (after 3 doses of PCV13). Wilcoxon test; ***=P<0.001. D. Lynn unpublished data.

In mice, Prof. Lynn's team has reported that antibiotic-driven dysregulation of the gut microbiota leads to impaired antibody responses to 5 different adjuvanted and live-attenuated vaccines (including BCG) that are administered to >100 million infants worldwide annually[3]. Furthermore, unpublished data from the Antibiotics and Immune Responses (AIR) infant observational cohort study, has revealed that antibiotic exposed infants have significantly reduced vaccine-specific (e.g. PCV13, diphtheria, Hib) antibody titres in serum (Fig. 1; PCV13 data shown) and altered blood gene expression responses following immunisation. To date, only two small (n=11-21) interventional clinical studies have attempted to address the role of the gut microbiota in regulating responses to vaccination. Rotavirus vaccine IgA titres were not significantly altered in participants randomised to antibiotics prior to immunisation[11], but neutralization and binding antibody titres were significantly impaired in individuals randomised to antibiotics who were immunised with trivalent influenza vaccine [12]. These data, together with a growing number of other animal model and observational clinical studies[1,13], strongly suggest that the gut microbiota is a crucial factor regulating antigen-specific immune responses to vaccination. Despite this progress, the mechanisms through which the gut microbiota regulates responses to vaccination in humans remain poorly understood.

In addition to inducing protective antigen-specific immune responses, vaccines can elicit non-specific effects (NSEs) on morbidity and mortality unrelated to the targeted disease[1]. The vaccine with the strongest evidence of NSEs is the BCG vaccine. Multiple epidemiological studies and randomised controlled trials (RCTs) have shown that BCG vaccination is associated with reduced risk of neonatal mortality by up to 50%[14,15], reducing deaths from infections other than tuberculosis (e.g. neonatal sepsis). In adults, BCG vaccination has been shown to reduce the risk of respiratory viral infections in the elderly[16] and a RCT in the Netherlands found that BCG-immunised adults had significantly reduced viral titres following challenge with an attenuated virus[17]. While the mechanisms underlying vaccine NSEs are incompletely understood, there is now substantial evidence that BCG NSEs are mediated, at least in part, via trained immunity – a microbial ligand-induced epigenetic and metabolic reprograming of innate immune cells, including progenitor haematopoietic stem cells in bone marrow[18,19]. This reprogramming leads to heightened innate immune responses to subsequent unrelated infections for up to 12 months post-vaccination[20]. Whether the gut microbiota regulates non-specific vaccine immunity is largely unknown. Only one study to date has reported associations between specific bacterial species and trained cytokine responses following BCG vaccination[21], however, a causal relationship and the mechanisms involved remain to be demonstrated.

To investigate whether the gut microbiota regulates vaccine mediated pathogen-specific or non-specific protection, we will use an acute antibiotics pre-treatment to significantly deplete the gut microbiota prior to BCG immunisation and will then assess whether this perturbation of the gut microbiota alters the efficacy of BCG against a secondary challenge 3 or 6 months later. We selected BCG for this study because **a)** BCG is a live vaccine; **b)** is one of the world's most widely used vaccines and the only licenced vaccine against TB, a disease that resulted in 10M cases and 1.5M deaths in 2020[22]; **c)** for reasons that are poorly understood, BCG is associated with highly geographically variable disease-specific protection (nil to 80%)[2]; **d)** we have shown in preclinical models that responses to BCG are regulated by the gut microbiota[3] and **e)** feasibility – PIs Lynn and Barry have experience running a major BCG vaccine trial (BRACE; NCT04327206)[23].

Currently, there are no established immunological correlates of protection for BCG (though T cells and IFNγ are required)[2] and obviously challenging humans with virulent *M. tuberculosis* (*Mtb*) is not ethical. Therefore, to assess whether depletion of the gut microbiota prior to BCG immunisation leads to impaired mycobacterial-specific immunity, we will use an innovative experimental medicine model in which participants are rechallenged with a second dose of BCG (i.e. live *M. bovis* BCG) into the opposite arm 6 months following immunisation/placebo. This model has been pioneered by Prof. Helen MacShane at Oxford University, who has demonstrated that BCG immunisation protects against subsequent BCG challenge in mice, cattle, non-human primates, and humans and that efficacy against BCG re-challenge predicts protection against virulent *Mtb* in animal models[24]. Briefly, 2 weeks after BCG challenge, a 4mm skin punch biopsy of the challenge site will be collected, and mycobacterial load will be quantified by solid culture and BCG-specific qPCR (protocols already established in PI Lynn's lab) as a measure of the anti-mycobacterial efficacy of BCG immunisation in antibiotic treated and untreated participants.

Whether the gut microbiota plays a role in regulating BCG-induced non-specific protection against other (non-mycobacterial) infections is currently unknown. As with BCG-induced mycobacterial-specific immunity, there are no known immune correlates of BCG-induced non-specific immunity; though enhanced emergency granulopoiesis[25] and trained immunity[17,18] play important roles. The most robust way to assess BCG-induced non-specific immunity is therefore to assess the extent to which BCG vaccination protects against an unrelated infection/challenge. Obviously, human challenge with a virulent organism is likely to be unethical/infeasible. We will therefore assess BCG non-specific immunity against an attenuated viral vaccine strain, the Yellow Fever Vaccine (YFV) virus. YFV is a licensed live vaccine, safely administered to millions of infants and adults worldwide each year, but which induces a mild viremia following immunisation. Previous work has shown that BCG immunisation reduces the level of YFV viremia after YFV vaccination, which correlates with induction of cytokine responses indicative of trained immunity. BCG did not alter YFV-induced B or T cell responses[17] indicating these effects are innate mediated. This human challenge model therefore allows us to robustly but safely quantify the effect of depleting the gut microbiota prior to immunisation on BCG-induced non-specific immunity.

This innovative study that will conclusively demonstrate in humans whether disruption of the gut microbiota prior to immunisation leads to impaired pathogen-specific and non-specific responses induced by the BCG vaccine.

## 3.3 Risk assessment

### 3.3.1 Known potential risks

This study involves limited risk to participants.

**Risks related to antibiotics**

AEs associated with the use of oral vancomycin and/or neomycin are usually restricted to self-resolving GI symptoms unless significant serum concentrations occur (e.g. in renal impairment). Participants with known chronic kidney disease/renal insufficiency will therefore be excluded from the study.

Possible reactions to oral vancomycin:

- abdominal pain
- nausea
- diarrhoea
- vomiting
- flatulence

Possible reactions to oral neomycin:

- sore mouth
- anorectal pain
- diarrhoea
- mouth irritation
- nausea
- rectal irritation
- vomiting

These possible AEs are reversible and any symptoms are expected to subside upon cessation or completion of the regime. The short 3 day antibiotic treatment regime to be used in this protocol has been previously shown in other published studies to be well-tolerated and effective even in vulnerable populations (melanoma patients)[26].

However, to help participants randomised to antibiotics with the expected GI symptoms (nausea/diarrhoea), they will be given a prescription for antiemetic and anti-diarrhoeal medication (metoclopramide and loperamide) that will be dispensed by the RAH pharmacy along with the trial antibiotics, at no cost to the participant. Participants will be screened for allergies to metoclopramide and loperamide and will be advised to alert the medical monitor if symptoms persist, in order to initiate a formal medical review.

In this study, neither participants nor study investigators are blinded to the antibiotic treatment group allocation so prescribing these medications to only those participants randomised to antibiotics does not compromise the scientific integrity of the study.

A study doctor will be on call to advise participants if they experience any adverse events from the antibiotics. (S)AEs related to the antibiotics will also be assessed via a participant survey as detailed below.

**Risks related to BCG vaccination**

The BCG vaccine is a licensed vaccine that has been in use for ~100 years and is administered to ~100 million individuals per year[2]. Having an injection can sometimes cause very minor pain from the needle or be uncomfortable. The vaccination will be administered by a trained immunisation nurse[27].

Expected (common) reactions to BCG vaccination:

- A small swelling, redness and tenderness (measuring 0.5-1.5 cm in diameter) at the injection site appears within 1-2 weeks at the injection site. The local lesion evolves into a small ulcer. The ulcer heals over several weeks to months, usually healing into a small flat scar.
- Slightly swollen lymph nodes in the axilla in up to 10% of recipients, and usually resolve spontaneously.
- Skin lesions following vaccination are more common in people over 15 years or with revaccination[28,29].

Uncommon side effects of BCG vaccination (up to 1 in 100) [30, 31-33]:

- Large ulcer, abscess at the injection site (~2cm in diameter)[34]
- Keloid scar at injection site
- Swelling of lymph nodes in the armpit larger than 1 cm across

Rare side effects (up to 1 in 1000):

- Significant inflammation of lymph nodes in the axilla, sometimes with oozing ulcers, possibly abscess
- Fainting, seizures and convulsions (rare among patients receiving injections)

Very rare side effects (1–4 cases per million vaccinated people)[30]:

- Disseminated BCG infection has been reported rarely after BCG vaccination, mainly in immunocompromised individuals (who are excluded from the trial). The infection can spread throughout the body, including the bones (osteomyelitis)
- Allergic reaction or anaphylaxis (e.g. redness of the face and neck, swelling of the face, throat or neck, skin rash, breathing difficulties and collapse)

*Revaccination with BCG (BCG rechallenge arm only)*

Within our study design, 50% of participants in the BCG re-challenge arm will receive two doses of BCG vaccination: one at randomisation at Day 0, and one six months later at challenge. Revaccination with BCG is associated with an earlier, accelerated reaction which begins within 24–48 hours of vaccination with induration followed by pustule formation in 5–7 days and healing within 10–15 days[30].

BCG-associated AEs are also more common following revaccination. Previous data from the BRACE trial[34] (Trial registration: NCT04327206, ClinicalTrials.gov), found 4.3% of participants with a history of BCG vaccination experienced immunisation-site abscesses upon revaccination, compared with 1.5% of participants that received BCG vaccination for the first time (OR 3.6, 95% CI 1.7–7.5). Abscess onset was also faster in revaccinated participants (onset 16 days vs 27 days), however the median diameter of abscesses (2cm) was not different between revaccinated and first-time vaccinated participants.

*Current contraindications of BCG vaccination*

- BCG is contraindicated in immunocompromised individuals due to the risk of disseminated BCG infection[28,30]. This includes individuals immunocompromised by HIV infection, primary immunodeficiencies, corticosteroids or other immunosuppressive agents, and malignancies involving bone marrow of lymphoid systems.

- BCG is also contraindicated in individuals with any serious illness and those with generalised septic skin diseases and active skin conditions such as eczema, dermatitis and psoriasis near the site of vaccination[30].
- While BCG has not been shown to cause foetal damage, the use of live vaccines is contraindicated in pregnancy[27].
- Individuals who have previously had tuberculosis or a large tuberculin skin test reaction.

*Pre-vaccination screening*

Interferon gamma release assay (IGRA) screening aims to identify individuals with latent tuberculosis infection (LTBI)[33]. A positive IGRA indicates either current or past infection with TB[33]. Participants who are deemed to be at an elevated risk of having LTBI (see eligibility survey details below) will be screened (after giving written informed consent) via an IGRA before being enrolled in the study. A negative IGRA result will enable the participant to enrol in the study. Participants who test positive to the IGRA test will be referred to the TB Clinic at the RAH.

**Risks related to Yellow Fever vaccination**

Having an injection can sometimes cause very minor pain from the needle or be uncomfortable. The vaccination will be administered by a trained immunisation nurse[27].

Expected (common) reaction to YF vaccination[35]:

- Headaches
- Myalgia
- Low-grade fever
- Mild or moderate tiredness
- Local reactions (minor injection site pain, erythema and swelling)

Very rare side effects (Less than 1 in 100,000)[36,37]

- Yellow Fever vaccine-associated neurotropic disease (YEL-AND; systemic symptoms accompanied by encephalitis or other neurological symptoms) has been reported, mostly in those <9 months or >60 years of age, or in immunocompromised individuals.
- Yellow Fever vaccine-associated viscerotropic disease (YEL-AVD; systemic symptoms accompanied by hepatitis) has been reported, mostly in individuals >60 or immunocompromised individuals.
- Guillain-Barré syndrome, an uncommon sickness of the nervous system in which a person's own immune system damages the nerve cells, causing muscle weakness, and sometimes, paralysis

*Current contraindications of YF vaccination*

- YF vaccination is contraindicated in individuals with history of severe allergic reactions to egg or chicken proteins
- YF vaccination is contraindicated in immunocompromised individuals, including individuals who have HIV infection, primary immunodeficiencies (including inherited IFNAR1 deficiency), or are taking corticosteroids or other immunosuppressive agents
- YF vaccination in contraindicated in haematopoietic stem cell transplant recipients

- YF vaccination is also contraindicated in individuals with thymus disorders, including myasthenia gravis, thymoma, thymectomy, DiGeorge syndrome or thymic damage due to chemoradiotherapy or graft-versus-host disease.
- YF vaccination is also contraindicated in individuals who are pregnant or breast-feeding.

**Risks related to Placebo injection**

Having an injection can sometimes cause very minor pain from the needle or be uncomfortable. The 0.9% NaCl is an inert salt solution that will not cause any degree of local reaction. The placebo injection will be administered by a trained immunisation nurse.

**Risks related to blood sample collection**

Having a blood test can sometimes cause some pain from the needle or be uncomfortable. Occasionally a small amount of bruising can occur on the skin where the blood was taken. On rare occasions, a participant can have a vaso vagal reaction (SOP-006, see Attachment 25). Trained members of the study team will collect the blood samples from participants.

**Risks related to punch biopsy at BCG site**

Having a punch biopsy involves local anaesthetic to numb the biopsy site, this can cause discomfort. The sample of skin is taken from the numbed area and occasionally the site requires a stitch, which will require the participant to return to have it removed 7-10 days after. The skin heals within 1-2 weeks. The risk of bleeding or infection is low. This procedure is performed by a trained clinician.

### 3.3.2 Assessment of potential risks

The administration of the antibiotics, vancomycin and neomycin, may cause some gastrointestinal symptoms. However, any symptoms are expected to subside upon cessation or completion of the regimen. While there’s no known impact of these antibiotics on the effectiveness of oral contraception, it will be recommended participants using oral contraceptive pills to prevent pregnancy take extra (barrier) precautions while taking the antibiotics and for up to until the time of their next menstrual cycle.

BCG and YF vaccination have a well-established safety profile in healthy individuals. While there are known adverse reactions to BCG, serious adverse reactions are rare. BCG vaccination does also cause a scar in over 80% of participants. Participants will be screened prior to BCG vaccination to ensure they have no known contraindications for BCG vaccination. Vaccination will be done by staff trained in intradermal injection under the direction of PI Barry (Head SA TB Services clinic and RAH site PI for the BRACE trial) to reduce the potential of subcutaneous injection which can increase scarring.

The Respiratory Clinical Trials Unit at the RAH was responsible for administering the BCG vaccine for the BRACE trial. During this trial, participants were successfully given the BCG vaccine as an intervention outside of standard regulations. This experienced team will be administering the vaccines for this study.

Live vaccines such as the BCG and YF vaccines are not recommended in pregnancy; as such, pregnant women are excluded from the study. Participants will be offered an optional pregnancy test at screening.

Since we will be using BCG and YF vaccines outside of their standard/recommended use, as per use of any intervention outside of standard regulations, we will be assessing the reactogenicity and

safety of both vaccinations in participants. We have previously monitored safety of BCG vaccination in the South Australian arm of the BRACE trial, with no SAEs.

# 4 STUDY OBJECTIVES AND OUTCOMES

## 4.1 Primary objective

Determine if depletion of the gut microbiota leads to a) impaired BCG-induced protection against mycobacterial infection in an experimental medicine BCG rechallenge model (BCG re-challenge arm) and b) impaired BCG-induced protection against other infections in an experimental medicine attenuated virus infection model (YF vaccine arm). This will be assessed by primary and secondary endpoints:

**Primary Endpoint:**

- Arm 1: Mycobacterial load (Colony Forming Units (CFU)) in the skin biopsy site in BCG-vaccinated participants not exposed to antibiotics (BCG-No ABX) compared to BCG-vaccinated participants that were exposed to antibiotics (BCG-ABX).
- Arm 2: Viraemia (viral copies/ml blood) at day 3-7 post YF vaccine challenge in BCG-vaccinated participants not exposed to antibiotics (BCG-No ABX) compared to BCG-vaccinated participants that were exposed to antibiotics (BCG-ABX).

**Secondary Endpoints:**

Arm 1:

- Mycobacterial load (CFU) in the skin biopsy site in BCG-vaccinated participants compared to placebo-vaccinated participants.
- Anti-mycobacterial T cell responses (IFNγ production and activation induced marker expression), antibody responses, and other immune parameters in peripheral blood collected from BCG-vaccinated participants not exposed to antibiotics (BCG-No ABX) compared to BCG-vaccinated participants that were exposed to antibiotics (BCG-ABX).

Arm 2:

- Viraemia (viral copies/ml blood) at day 3-7 post YF vaccination in BCG-vaccinated participants compared to placebo-vaccinated participants.
- Comparison of innate immune responses (e.g. PBMC cytokine responses following in vitro stimulation, expression of activation markers on immune cells, frequency of immune cell populations in peripheral blood) at baseline (prior to BCG vaccination), at 3 months (prior to YF vaccination) and following YF vaccination in all groups (i.e. BCG/Placebo with and without prior antibiotic exposure).

## 4.2 Secondary objective

Employ a systems immunology approach to determine how BCG mediates anti-mycobacterial immunity in the skin and alters immune responses following YF vaccination. We will also employ a host of systems immunology techniques to identify components of the gut microbiota and immunological signatures associated with BCG-specific and non-specific immunity:

- Longitudinal analysis of blood samples through the study: immune cell frequencies and activation marker expression, molecular analysis of proteins, lipids and bacterial metabolites.
- Blood transcriptomics to measure gene expression (i.e. which genes turned on or off).
- Bacterial load and microbial composition of the stool microbiota at D0 and 3 or 6 months compared to D-3 in all participants.
- Correlative analysis of metagenomic, transcriptomic, immunological and epigenomic datasets.
- Arm 1 only: Standard histology and immunohistochemistry of the BCG rechallenge site in BCG-vaccinated vs non vaccinated participants.
- Arm 1 only: Spatial transcriptomics (measuring where genes are expressed in tissue) of the BCG rechallenge site in high vs. low responders (as assessed based on *M. bovis* BCG bacterial load in the biopsy site).
- Arm 2 only: Comparison of genome-wide chromatin accessibility (i.e. which parts of the genome are "open" or "closed") in peripheral blood monocytes, neutrophils and NK cells across all groups.

## 4.3 Outcomes

**Arm 1:** We will determine whether participants with a depleted gut microbiota, at the time of BCG immunisation, have significantly reduced mycobacterial load following BCG rechallenge. These data will provide conclusive evidence in humans as to whether the gut microbiota regulates the pathogen-specific immunity induced by the BCG vaccine.

**Arm 2:** We will determine whether participants with a depleted gut microbiota, at the time of BCG immunisation, have significantly reduced viremia following challenge with a live attenuated virus (YF vaccine). These data will provide conclusive evidence in humans as to whether the gut microbiota regulates non-specific immunity induced by the BCG vaccine against other (non-mycobacterial) infections.

Our microbiota analyses will enable us to determine which bacterial species are associated with protective immune responses following BCG vaccination. Our exploratory immunological analyses in this study will also provide mechanistic insight into how the microbiota influence immune responses, and how local immune responses in the skin correlate with peripheral immune responses in the blood following BCG vaccination.

Together, these data may provide evidence for the future development of microbiota-targeted therapies to improve vaccination efficacy or other immune outcomes.

# 5 STUDY DESIGN

## 5.1 Overall design

This is an experimental medicine study divided into two arms. In the first arm we will determine if depletion of the gut microbiota leads to impaired BCG-induced protection against mycobacterial infection in a controlled human experimental BCG rechallenge model in 178 participants (BCG rechallenge arm) (including the 10 participants in the pilot study, see section 8.3). In the second arm, we will determine if depletion of the gut microbiota leads to altered protection against a live attenuated virus (YF vaccine) in 180 participants (YF vaccine arm).

Participants in both arms (358 total) will be randomised to receive a 3 day course of antibiotics or none (ABX vs comparator groups) at Day -3. The antibiotics will be orally administered (500mg vancomycin and 1000mg every 6 hrs for 3 days) and will significantly deplete the gut microbiota.

At Day 0, following ABX (or no ABX) administration, the 2 groups are further randomised to receive BCG vaccine or placebo injection into the left arm. Following this, the two arms will receive different challenge vaccines (**Figure 2a-b**).

**Arm 1:**

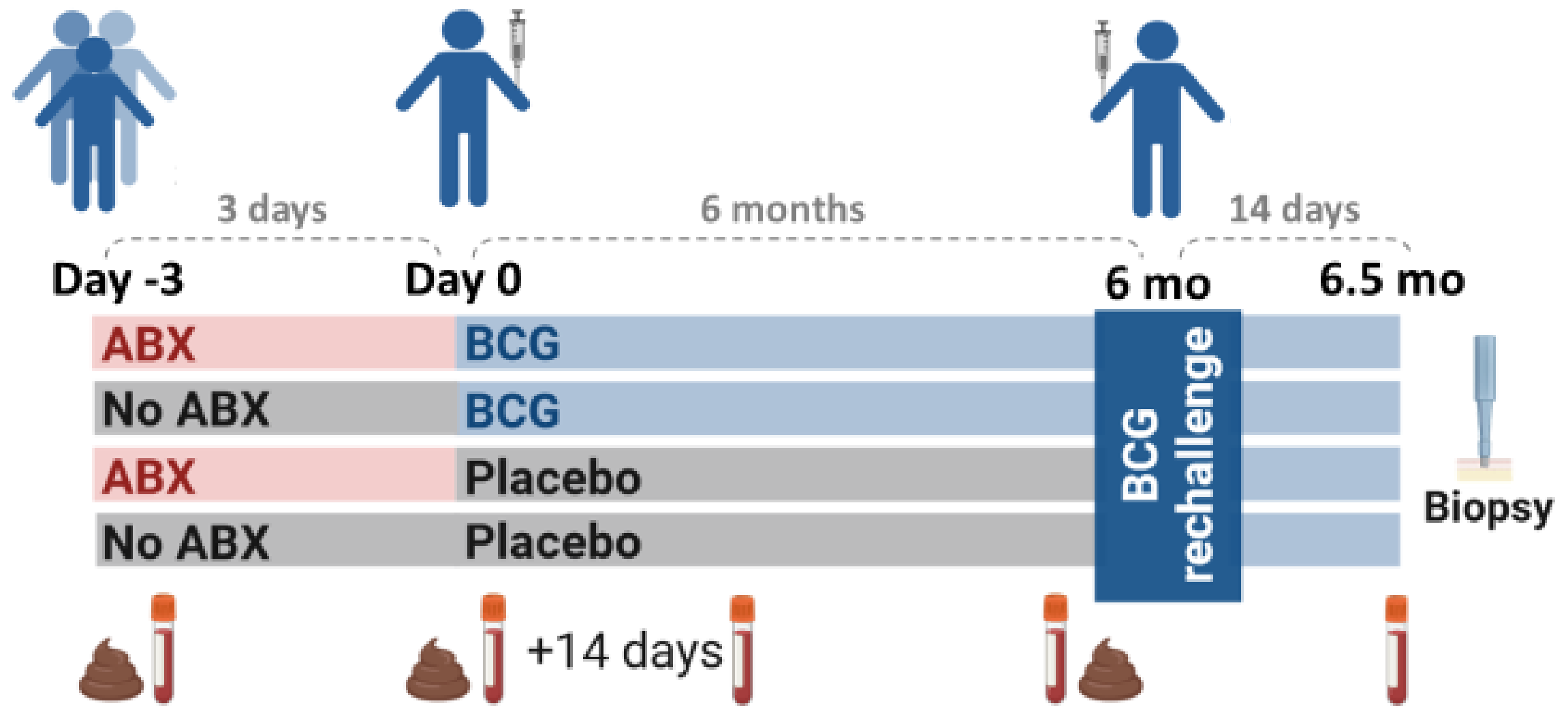


**Figure 2a. Study design for Arm 1 (BCG rechallenge arm).** 6 months following randomisation, all participants in the BCG re-challenge arm, will receive a BCG vaccine in the right arm. A punch biopsy will be performed two weeks following the BCG re-challenge.

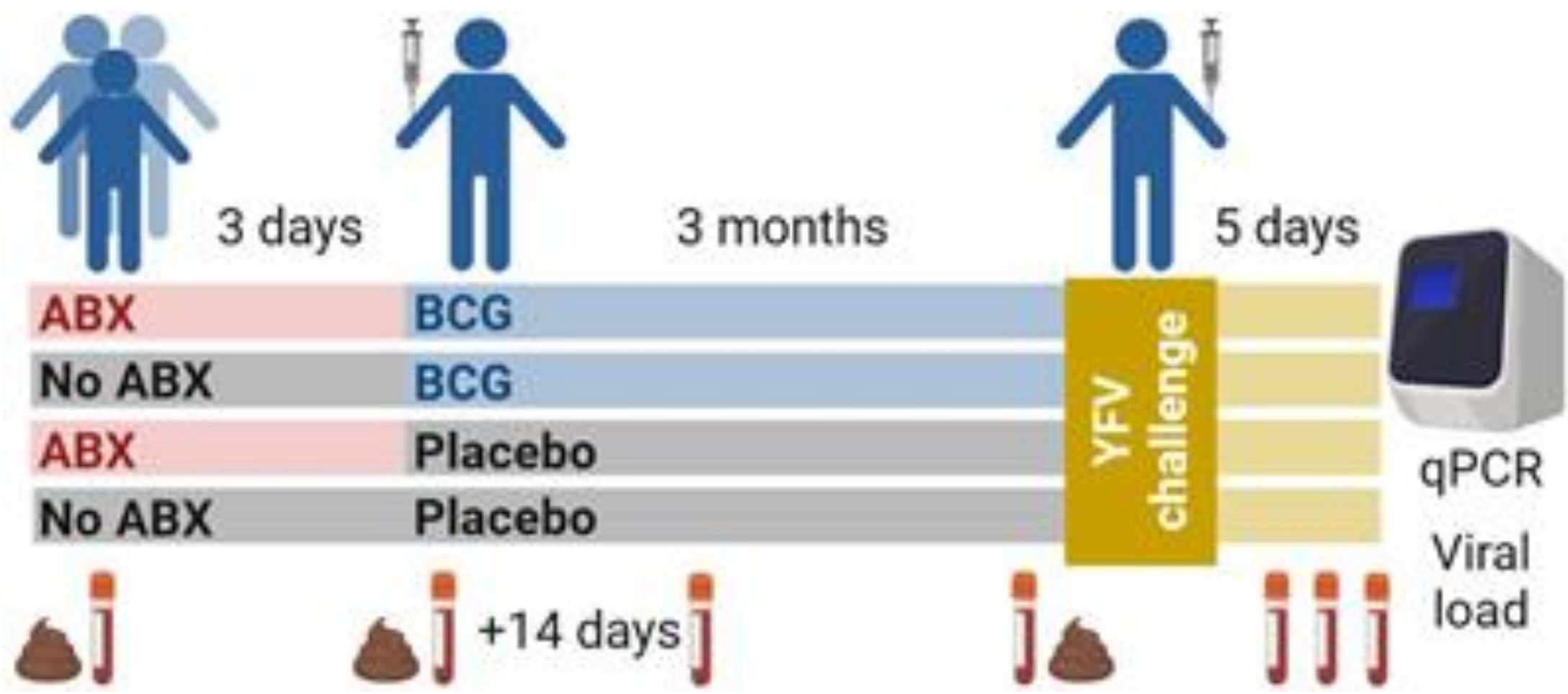


**Figure 2b. Study design for Arm 2 (YF vaccine arm)**. 3 months following randomisation, all participants in the YF vaccine arm, will receive a YF vaccine in the right arm.

Blood and stool samples will be collected at regular intervals.

The study duration time for participants is 4 months for those in YF vaccine arm and 7 months (5 months with no visits) for the BCG re-challenge arm.

## 5.2 Justification for the doses

The dose and duration of vancomycin and neomycin are based on two previous published clinical studies that have used the same dose and duration of antibiotics to investigate the role of the microbiota in responses to immunotherapy in melanoma patients[26] and in the FARMM (Food and Resulting Microbial Metabolites) study[38]. The protocol was well-tolerated and there was a marked reduction in both culturable bacteria and 16S rRNA gene copy number of about 4-5 logs after 72 hours indicating significant depletion of the gut microbiota.

The dose and route of administration of the BCG and YF vaccinations are the standard accepted dosage when used to prevent TB and YF, respectively. There is no justification to vary from this.

## 5.3 Study population

### 5.3.1 Eligibility criteria

- 18-35 years old
- Provided a signed and dated informed consent form
- BCG naïve (Arm 1) and BCG and YF vaccine naïve (Arm 2)
- Willing to take short antibiotic course
- Willing to undergo a punch biopsy (Arm 1)
- Willing to have up to 7 blood samples and 3 stool samples collected over 5-7 months
- Not pregnant or intending to get pregnant for the duration of the study (a pregnancy test will be offered to WOCBP)

### 5.3.2 Exclusion criteria

- Previous BCG
- YF vaccination or previous YF infection (excluded from YF arm, eligible for BCG arm)
- Evidence of latent TB infection (LTBI) (assessed through a questionnaire)
    - IGRA to confirm if needed
- People with contraindications for BCG vaccination:
    - malignancies involving bone marrow or lymphoid systems, primary or secondary immunodeficiencies, HIV infection
    - moderate/severe skin disease including eczema, dermatitis or psoriasis
    - requiring immunosuppressive drugs or other immune modifying drugs e.g. corticosteroids, non-biological immunosuppressants, biological agents (such as monoclonal antibodies against tumour necrosis factor (TNF)-alpha)
- People with contraindications to YF vaccination:
    - History of thymus disease, including myasthenia gravis, thymoma, thymectomy, DiGeorge syndrome, thymic damage from chemoradiotherapy or graft-versus-host disease
    - YF vaccination is contraindicated in immunocompromised individuals, including individuals who have HIV infection, primary immunodeficiencies (including inherited IFNAR1 deficiency), or are taking corticosteroids or other immunosuppressive agents
    - People who have had a haematopoietic stem cell transplant
    - Individuals with history of severe allergic reactions to egg or chicken proteins
- Pregnant or breastfeeding or planning to become pregnant

- History of renal disease/insufficiency
- Tattoo obscuring BCG vaccination site(s)
- Any history of severe allergic reaction or anaphylaxis to vaccination or antibiotics
- People with chronic serious underlying illness
- Have received any prescribed oral or intravenous antibiotic in the 28 days prior to study visits 1 and 4 (including isoniazid, rifampicin, streptomycin and ethambutol as these particular antibiotics have activity against *M. bovis*)

## 5.4 Screen failures

Screen failures are defined as participants who have consented but become ineligible during the screening process and are withdrawn from the study. They are not randomised and do not receive the intervention.

## 5.5 Recruitment and consent

Healthy adults (18-35 years) will be provided information (i.e. the AVIRS Flyer, AVIRS Participant Communication, AVIRS PICFs (separate PICFs for each arm); see attachments 1-4) about the study via email distribution lists (SAHMRI and University of Adelaide, University of South Australia and Flinders University, TAFE, CALHN staff and student email distribution lists), mainstream media, social media and institutional notice boards and/or newsletters. Permission will be obtained from SAHMRI, CAHLN Communications and Engagement team at the Royal Adelaide Hospital, University of Adelaide, University of South Australia, Flinders University and TAFE before advertising for recruitment of participants. The study will also be advertised to CALHN staff via public notice boards, staff bulletins and social media pages, etc. using advertising material/wording approved by the CALHN Communications and Engagement team.

Any healthy adult, meeting the eligibility criteria, who are willing to provide consent and attend each visit, will be eligible to enrol. Communications (see attachment 2) will provide instructions for potential participants to approach the Study Coordinator (via email or phone) for further information about participating in the study or to sign up for the study via a REDCap survey.

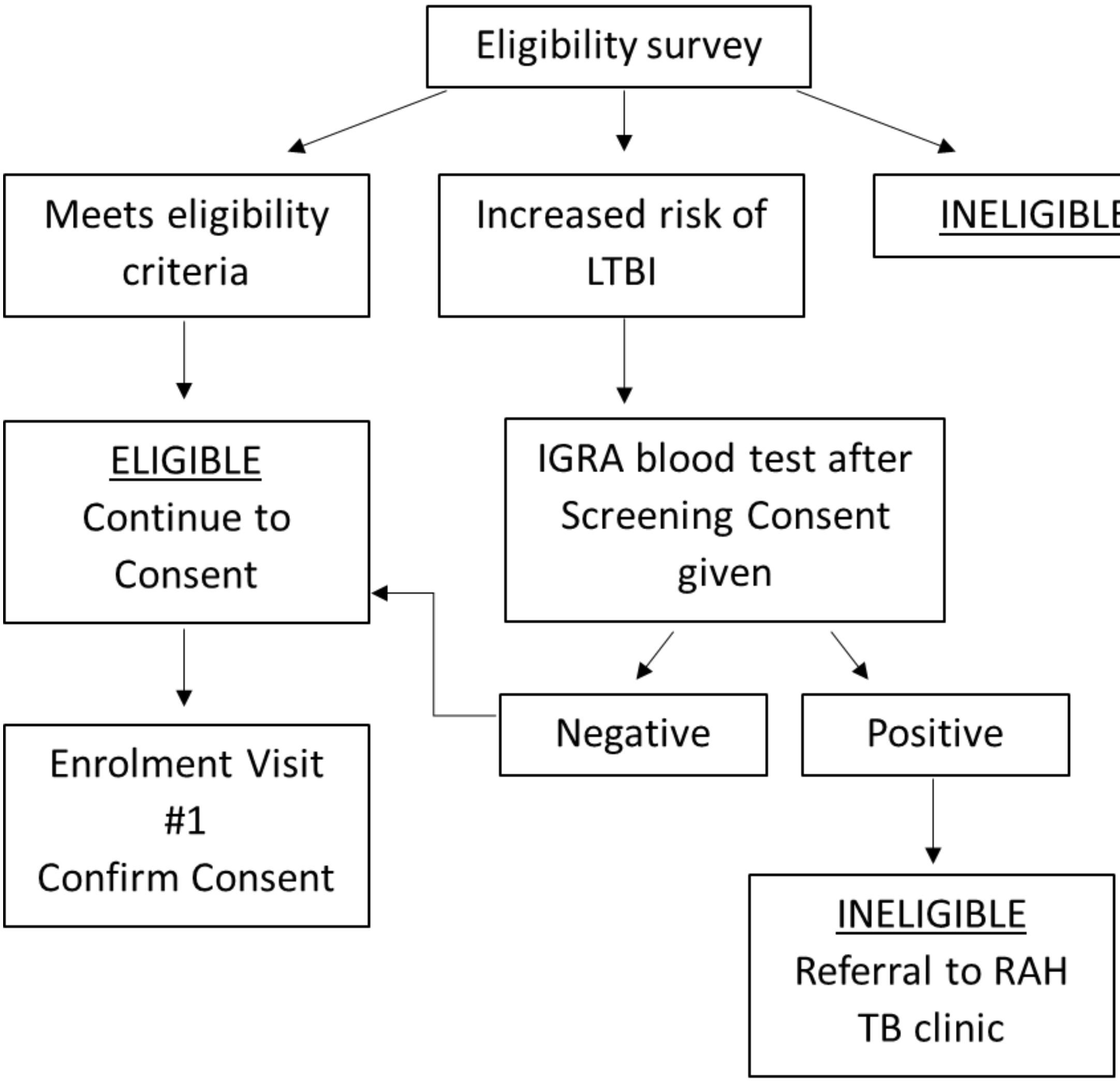


**Figure 3. Eligibility Pathway**

Potential participants will be able to evaluate their eligibility online via a REDCap public link. Participants will be asked to consent to answering screening questions before any data is collected. This screening consent will be obtained electronically (see attachment 5: AVIRS Screening and Consent CRF V4.0) by asking the participant:

"To determine whether you are eligible to participate in this study we would like to ask you a few personal questions including questions about your age, where you were born, previous vaccinations and medical history. The answers you give will be kept confidential.

Do you agree to the collection of your personal information for screening purposes?".

The participant will be given a choice to answer yes or no. If they answer no, no information will be collected, and they will not be able to proceed to the eligibility survey. If they answer yes, they will proceed to complete the eligibility survey. If the participant is unsure, they will be able to contact the study team.

Once the participant has consented to answer the screening questions, a modified questionnaire used routinely by the SA TB Services will assess whether a participant is low risk of TB infection and for the purpose of the study will be considered TB naïve or has potentially been exposed to TB requiring further assessment. This questionnaire is included in the eligibility survey. Participants who

answer ‘no’ to the relevant questions in the questionnaire are considered TB naïve and are able to continue to consent to participate in the study.

If a participant answers ‘yes’ to any high risk TB questions, they will be asked to sign a Screening Blood Test Consent Form (see attachment 6) and offered an interferon gamma release assay (blood test) to determine if they have TB infection. A positive interferon gamma release assay deems the participant ineligible to participate in the study. The participant will be contacted by a clinical member of the study team, all of which are TB physicians, counselled and offered a referral to SA TB Services (see attachment 7). A negative result deems the participant to be TB naïve and eligible for the study. They will be able to continue the Consent process.

Once the participant has met the eligibility criteria, they will be assigned to the BCG (Arm 1) or YF vaccine (Arm 2) arms of the study. Participants who have indicated prior YF vaccination will be assigned to Arm 1, the remaining participants will be non-randomly assigned to Arm 1 or Arm 2 depending on recruitment needs. Eligible participants will then be contacted by the study coordinator to discuss the study, answer any questions and offered online or in person consent. Participants will be provided with a copy of the relevant participant information and consent form (PICF) based on their allocation to study arm. Two separate PICFs will be used for each arm of the study (to avoid confusing the participants, given different procedures in the two arms). Participants will receive the PICF relevant to the arm they were enrolled in only.

Electronic consent will be given by a two-stage process (see attachments 5 and 8: AVIRS Screening and Consent CRF v4.0 and AVIRS Eligibility and Consent Confirmation CRF v4.0). Participants will read the relevant PICF via REDCap and after answering questions to ensure informed consent is given, sign the PICF electronically. Participants will be emailed a copy of the signed PICF. Participants can contact the study team at any time to discuss any questions. Alternatively, the participant can opt to consent in person on the first visit.

The study coordinator will contact the participant to arrange for them to attend the clinical trials clinic at SAHMRI (or alternatively, the TB clinic at the RAH), for an enrolment visit to confirm informed consent. Both sites have purpose-built clinical consulting rooms that contain equipment and supplies needed for clinic trial visits.

At the enrolment visit, the Site PI or delegate will explain the study and confirm the participant is still eligible. The Site PI or delegate can also deem a participant ineligible for other medical reasons according to their professional opinion. Once the Site PI or delegate is satisfied that the participant understands the study and has given informed consent, the Site PI or delegate will sign the declaration in REDCap. Only then will the participant be considered fully consented to the study.

Participants will be given time to consider their participation and ask questions. Consent is voluntary and free from coercion. Participants are free to withdraw from the study at any time.

A pro-rated reimbursement of $500 for the YF vaccine arm and $750 for the BCG rechallenge arm is given to the participants on completion of the study (a lesser amount is given if the participant voluntarily withdraws from the study early). The participant reimbursement cost is intended to be consistent with NHMRC recommendations on participant reimbursements to provide fair recompense to participants without undue inducement to participate in the research[39].

The YF vaccine arm will have a 4 month study timeframe with 7 clinical visits including consent, 7 blood draws, 3 stool samples, antibiotics administration (in the ABX group), 1 BCG/Placebo vaccination, and 1 YF vaccination justifying $500/participant for time and inconvenience. The BCG

rechallenge arm will have a 7 month study timeframe with 5 clinical visits including consent, 5 blood draws, 3 stool samples, antibiotics administration (in the ABX group), 1-2 BCG vaccinations, and 1 skin biopsy justifying $750/participant for time and inconvenience. Given the longer study duration and the more invasive skin biopsy in the BCG rechallenge arm, we believe a higher amount for this cohort is justified. The participant reimbursement amounts were peer-reviewed as part of the successful NHMRC Ideas Grant proposal awarded to PIs Lynn and Barry.

## 5.6 Enrolment visit

At the enrolment visit the following will be occur:

- Informed consent from the participant, if they choose to consent in person rather than electronically
- Participant eligibility and informed consent confirmed
    - Site PI or delegate will sign the declaration in REDCap (see Section 5.5)
- TB naïve status confirmed based on their response to questions in the modified questionnaire routinely used by the SA TB Services to determine risk of TB exposure, (including checking results if participant was sent for an IGRA blood test)
- YF vaccination/infection naïve status will be confirmed, for YF vaccine arm only
- Pregnancy test (urine) discretely offered, if required
- Baseline blood sample of 60ml taken (see attachment 9 – SOP-001)
- Randomisation to ABX or No ABX groups (unblinded) and BCG or placebo groups (blinded)
    - If randomised to ABX, participant is given a prescription by the Site PI or delegate to be taken to the RAH Clinical Trials Pharmacy to receive antibiotics
- 2 stool sample kits given to participants with instructions explaining how to collect at home (see attachment 10)

# 6 BCG VACCINATION AND CHALLENGE VACCINATION

## 6.1 Treatment arms

- Antibiotics group: Vancomycin and Neomycin tablets
- Comparator group: no treatment given

And

- Intervention group: BCG vaccine
- Placebo group: 0.9% Saline

Challenge vaccine: BCG (Arm 1) or YF vaccine (Arm 2)

## 6.2 Study interventions

### 6.2.1 Antibiotics

Participants randomised to the antibiotic group will receive an oral antibiotic regimen that includes 500mg of vancomycin and 1000mg of neomycin every 6 hours for a duration of 72 hours. Vancomycin is a glycopeptide used for the treatment of Gram-positive bacteria unresponsive to other antibiotics. Neomycin is an aminoglycoside antibiotic that displays bactericidal activity against Gram-negative aerobic bacilli and some anaerobic bacilli. They are thus effective at significantly depleting the gut microbiota as demonstrated in two previous clinical studies[26,38].

To help participants randomised to antibiotics with the expected GI symptoms (nausea/diarrhoea) they will be given a prescription for antiemetic and anti-diarrhoeal medication (metoclopramide 10 mg TDS PRN and loperamide 2mg) that can be filled, if needed, at the RAH pharmacy at no cost to the participant. Participants randomised to the antibiotic arm will be instructed to take metoclopramide 10mg tablets up to three times daily for up to three days for nausea or vomiting, and to take loperamide 2mg every 4 hours or after each loose bowel motion up to a maximum daily dose of 16mg for a maximum of three days.

A comparator group was chosen rather than a Placebo group as the outcomes are objective measurements (bacterial load and immunological parameters) rather than subjective measurements (participant reported measurements). In addition, the randomisation to groups would be immediately apparent to the study team from the stool sample microbiome analysis (where a 4-5 log fold reduction in bacterial load is expected). Therefore, blinding of the randomised group as well as the additional cost of providing placebo tablets is unnecessary.

### 6.2.2 BCG vaccine

Each 0.1ml dose of reconstituted vaccine from the freeze-dried product contains between 2 to 8 x $10^5$ colony forming units of live attenuated bacteria of the type *M. bovis* BCG (Bacillus Calmette-Guerin), Danish strain 1331.

### 6.2.3 Placebo to match BCG vaccine

Sodium Chloride 0.9% from a 10ml ampoule.

### 6.2.4 Yellow Fever vaccine

Each 0.5ml dose of reconstituted Yellow Fever vaccine from freeze-dried product contains an injectable suspension in stabiliser of >1000 IU of the attenuated 17D strain of the Yellow Fever virus.

## 6.3 Dosage

A single dose of BCG vaccine or matched placebo will be given to all participants who are randomised. The adult dose is 0.1 mL (of BCG vaccine or 0.9% NaCl) injected intradermally over the distal insertion of the deltoid muscle onto the humerus (approximately one third down the upper left arm).

A single dose of BCG vaccine will be given to all participants in the BCG rechallenge arm of the study (this will be the second BCG vaccine for 50% of participants in this arm) 6 months following randomisation. This dose of BCG will be administered to the right arm.

For the YF vaccine arm of the study, a single dose of YF vaccine will be given subcutaneously in the right arm to all participants 3 months following randomisation.

## 6.4 Dose modification

There are no allowable dose modifications.

## 6.5 Storage and dispensing of vaccines

BCG vaccine

- The BCG vaccine will be stored at the Royal Adelaide Hospital (RAH) in accordance with the national vaccine storage guidelines, in a specifically designated, alarmed, and remotely-monitored vaccine fridge between 2°C – 8°C.
- **Temporary Storage at SAHMRI Clinics:** On the day of administration, the BCG vaccine will be transported from RAH vaccine storage fridge to SAHMRI clinic on ice in a temperature-

monitored insulated cooler box (Esky) between 2°C and 8°C during transport to the SAHMRI clinics. The temperature in the Esky must be recorded before the vaccine is administered to participants, and these logs must be entered into the REDCap database.

- Alternatively, the BCG vaccine can be stored in a scientific vaccine-grade fridge at SAHMRI, which is also alarmed and maintained within the recommended temperature range of 2°C – 8°C. This option can be used for the day of administration only, ensuring the vaccine remains within the correct storage conditions until it is administered.
- Store in the original package in order to protect from light
- Do not freeze
- Do not use the vaccine after the expiry date which is stated on the carton as "EXP" and refers to the last day of the month listed
- Any unused vaccine at the end of the study, meaning vaccines unused after the last dosing of the last participant will be disposed of according to local regulations unless it is forwarded to the AVIRS scientific team for assessment of CFU count at Visit 4 in the BCG Arm.
- The BCG vaccine log must be completed at each point the vaccine is removed from refrigeration, administered, discarded or forwarded to the AVIRS scientific team.

**Registration as an Approved Yellow Fever Vaccination Site (South Australia)**

To administer Yellow Fever vaccines as part of this study, the AVIR study through the RAH clinic has been registered as an approved Yellow Fever Vaccination site with the appropriate authorities in South Australia (SA Health). The site has been issued an official Yellow Fever Vaccination Site Certificate. This certification is valid for a set period and may require periodic renewal or inspection. The process for registration was as follows:

1. **Application to the Department of Health and Wellbeing (SA Health):**
    - The AVIR study clinical team at the RAH has been registered as an approved Yellow Fever Vaccination site with **SA Health**, the governing health authority in South Australia, to enable this vaccine to be administered as part of the study.
2. **Meeting the Storage Requirements:**
    - The RAH pharmacy, which stores the vaccine until the day of administration, has a monitored and temperature-logged vaccine-grade refrigerator for the storage of Yellow Fever vaccines, ensuring that the vaccines are stored between 2°C – 8°C at all times. The vaccine is transferred to SAHMRI for administration to participants on ice in a temperature monitored Esky.
    - Temperature records are maintained for monitoring compliance with the vaccine storage conditions.
3. **Staff Training Requirements:**
    - All personnel involved in the administration of Yellow Fever vaccines have been trained and certified in the handling, storage, and administration of Yellow Fever vaccines, including the recognition and management of adverse events following vaccination.
    - Staff are familiar with the specific vaccine storage and administration guidelines for Yellow Fever vaccines as provided by the vaccine manufacturer and relevant public health authorities.
4. **Submission of Required Documentation:**
    - The clinic has submitted documentation to SA Health confirming that staff members have been trained in Yellow Fever vaccination and that the premises meet the required standards for vaccine administration.

  - The clinic must also provide proof of proper storage facilities, including records of temperature monitoring for the vaccine fridge and any temperature excursions.
5. **Post-Approval Reporting and Compliance:**
   - The AVIR study must comply with ongoing reporting requirements, including the documentation of each Yellow Fever vaccination administered.
   - The AVIR study clinical team has been provided with an official yellow fever stamp (ID K1) by SA Health so that participants can be given an official certificate of yellow fever vaccination for their records/use when travelling in future.
   - The clinic must maintain records of vaccine usage, storage temperatures, and any adverse events.
   - Any changes to the clinic's vaccine storage, staff qualifications, or other relevant conditions must be reported to SA Health.

Yellow fever vaccine

- The Yellow Fever vaccine will be stored under the same conditions as the BCG vaccine, between 2°C – 8°C, at the RAH pharmacy, with all required monitoring systems in place.
- **Temporary Storage at SAHMRI Clinics**: The same Esky storage protocol applies for the Yellow Fever vaccine on the day of administration, with temperature logging before administration to participants. These temperatures must also be recorded in the REDCap database.
- Alternatively, the Yellow Fever vaccine may be stored in a scientific vaccine-grade fridge at SAHMRI for the day of use, maintained between 2°C – 8°C, to ensure proper storage conditions until the vaccine is administered. Store in the original package in order to protect from light
- Do not freeze
- Do not use the vaccine after the expiry date which is stated on the carton as "EXP" and refers to the last day of the month listed
- Any unused vaccine at the end of the study, meaning vaccines unused after the last dosing of the last participant will be disposed of according to local regulations.
- The Yellow Fever vaccine log must be completed at each point the vaccine is removed from refrigeration, administered, discarded or forwarded to the AVIRS scientific team.

Placebo – sodium chloride 0.9%

- Store less than 25°C
- Do not use after the expiry date which is stated on the carton and ampoule as "EXP" and refers to the last day of the month listed
- Any unused sodium chloride 0.9% at the end of the study, unused ampoules after the last dosing of the last participant will be disposed of according to local regulations

## 6.6 Preparation

BCG Vaccine

BCG Vaccine consists of a powder and solvent for suspension for injection (2-8 x $10^5$ CFU/0.1 mL dose).

Prior to reconstitution, the storage temperature of the BCG will be checked to ensure it the appropriate temperature has been maintained during storage, and transport (if applicable) unless

the storage and transport has occurred in validated containers or conditions where the temperature is stable and the data readily available.

The rubber stopper must not be wiped with any antiseptic or detergent. In the eventuality of alcohol being used to swab the rubber stopper, it must be allowed to evaporate before the stopper is penetrated with the syringe needle. The BCG is re-suspended using the solvent provided according to the product directions then carefully inverted a few times to produce uniform resuspension of the lyophilised BCG. Study staff must not shake the vial. The study staff member who re-suspends the BCG will label the vial with the date, time of reconstitution and their initials.

To ensure a uniform suspension, and therefore dose, the vial will be gently swirled before drawing up each dose. When drawn up into the syringe the reconstituted vaccine should appear homogeneous, slightly opaque and colourless.

Each vial of BCG contains up to 10 adult doses. Study staff must NEVER administer the whole vial. Each vial can be kept for up to 4 hours after resuspension. During this time the vial is kept between 2-8°C. Each vial is discarded after 4 hours, or when the vial is empty, whichever occurs first.

Yellow fever vaccine

Yellow Fever Vaccine (STAMARIL) is supplied as a single-dose lyophilised vial and a prefilled syringe of saline diluent (0.5mL).

For syringes without attached needle only: after removing the syringe tip cap, a needle should be firmly placed on the tip of the syringe and secured by rotating a quarter of a turn (90°).

The vaccine will be reconstituted by adding the solvent provided in the pre-filled syringe to the vial. The vial will be vigorously shaken, and after complete dissolution, the suspension obtained will be withdrawn into the same syringe for injection.

Sodium Chloride 0.9% placebo

Sodium chloride 0.9% will be decanted using aseptic technique into an empty sterile amber glass vial or prepared in 0.1 mL dosing syringes as per local vaccination practices. The study staff member who prepares the sodium chloride for injection will record the date, time of the preparation and their initials.

The prepared sodium chloride for placebo can be kept for up to 24 hours. During this time the placebo is kept between 2-25°C. All prepared syringes or vials unused at the end of a vaccination session will be discarded.

## 6.7 Administration of study drug

### 6.7.1 Antibiotics

Participants will meet with a study doctor for the enrolment visit and be randomised to the antibiotic group or not. Participants randomised to the antibiotics group will receive a prescription for the antibiotics which can be filled at the RAH Clinical Trials pharmacy. The RAH Clinical Trials Pharmacy will order and dispense the antibiotics to participants upon receipt of the study specific prescription.

The antibiotic regimen consists of 500mg vancomycin and 1000mg neomycin to be taken every 6 hours for a duration of 72 hours. These will be in tablet form for oral administration. Participants will be given a diary to record the times that the tablets were taken to measure compliance.

### 6.7.2 BCG and placebo injection

The BCG vaccine and placebo injection will only be administered by clinician members of the study team trained in the intradermal vaccination technique.

The vaccinator will follow the BCG/Placebo vaccination SOP (SOP-002 V2.0) and BCG rechallenge SOP (SOP-004 V2.0) as well as relevant safety requirements (see attachments 11 and 12). The vaccinator will explain the expected usual reaction to BCG vaccination. Participants will be given a "What to Expect" information sheet with contact details for the medical monitor if needed (see attachment 14).

Administration of the BCG vaccine placebo injection will take place in locations set-up by the study team prioritising participant safety, including ensuring appropriate facilities for management of any potential adverse event are available (e.g. anaphylactic reaction, extremely rare). There will be space to allow for privacy for the participant if required (e.g. upper left/right arm not accessible due to clothing).

As per standard practice, participants will be required to remain at the site for 20 minutes after vaccination, in case an allergic reaction should occur.

The time and date of resuspension of the vaccine vial, or placebo preparation, batch identifier, immunisation date/time, any issues with immunisation will be entered in the participants' study record in REDCap database (see attachment 16).

<u>Route/method of administration</u>

The injection site should be clean and dry using non-alcohol based antiseptic. Alcohol antiseptics should not be used prior to administration. If alcohol is used to swab the skin, it must be allowed to evaporate before the vaccine or placebo is injected. The vaccine or placebo must be given strictly intradermally, approximately one third down the upper arm corresponding to the area of the distal insertion of the deltoid muscle, as follows:

- The skin is stretched between thumb and forefinger
- The needle should be almost parallel with the skin surface and slowly inserted (bevel upwards), approximately 2 mm into the superficial layers of the dermis. The needle should be visible through the epidermis during insertion
- The vaccine or placebo should be given slowly

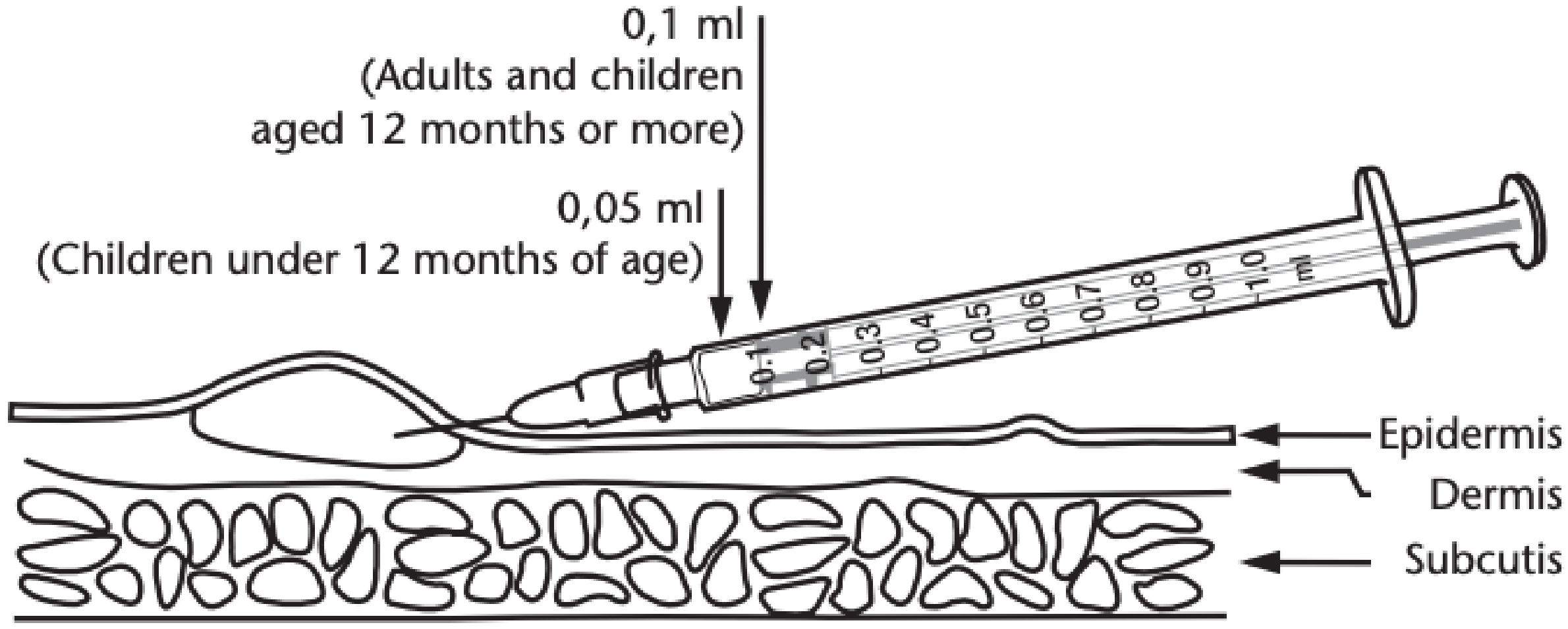


**Figure 4. Intradermal administration of BCG vaccine**

- The mixed vaccine should be administered with a syringe of 1 ml graduated into hundredths of millilitre (1/100) fitted with a short bevel syringe needle (Preference to use 25G or 26G, accepted up to 30G).
- You should feel considerable resistance as you give the injection. If there is no resistance, the needle may be in the subcutaneous tissues.
- If the injection is not intradermal, withdraw the needle and repeat at a new site.
- A raised, blanched papule/bleb of about 7 mm diameter (looks like orange peel) at the needle point is a sign of correct injection
- The injection site is best left uncovered to facilitate healing
- Jet injectors or multiple puncture devices should not be used to administer the vaccine.
- Photos of the vaccination site 14 days post vaccination (Visit 3) and challenge (Visit 5)

Over/under dosage or incorrect administration

Overdose increases the risk of suppurative lymphadenitis and may lead to excessive scar formation. Gross over dosage increases the risk of undesirable BCG complications. Deep injections increase the risk of lymphadenitis and abscess formation.

The immunisation nurse who administers BCG or placebo as part of this study will be required to document whether the vaccination was given ‘perfectly’ with appropriate bleb. Any variations will be documented, and standard procedures followed regarding the need for re-administration, notification to PI (or delegate).

Complications

All BCG-related complications will be referred to the medical monitor and PI Barry for advice regarding management. In the very unlikely event a participant has a systemic infection of *M. bovis* or persistent local infection following vaccination the PI will provide advice to the local treating team regarding management, including antibiotic treatment choice. Any serious adverse event or adverse event of interest occurring during the administration of the IP or the 20 minutes post administration will be documented appropriately according to the safety monitoring and reporting section of this protocol.

### 6.7.3 Yellow Fever vaccine

The YF vaccine will only be administered by clinician members of the study team trained in the subcutaneous vaccination technique. The vaccinator will follow the YF vaccination SOP (see attachment 13 - SOP-005 V2.0) and relevant safety requirements.

Administration of the YF vaccine will take place in locations set-up by the study team prioritising participant safety, including ensuring appropriate facilities for management of any potential adverse event are available (e.g. anaphylactic reaction, extremely rare). There will be space to allow for privacy for the participant if required (e.g. upper arm not accessible due to clothing).

As per standard practice, participants will be required to remain at the site for 20 minutes after vaccination, in case an allergic reaction should occur.

The time and date of resuspension of the vaccine vial, batch identifier, immunisation date/time and any issues with immunisation will be entered in the participants' study record in REDCap database.

<u>Route/method of administration</u>

The injection site should be cleaned and dried using non-alcohol based antiseptic. Alcohol antiseptics should not be used prior to administration. If alcohol is used to swab the skin, it must be allowed to evaporate before the vaccine is injected. The yellow fever vaccine will be given subcutaneously into the fatty tissue overlying the triceps muscle (about halfway down the upper arm), as follows:

- The skin is gently pinched between thumb and forefinger to avoid injecting into muscle
- The needle should be inserted at a 45° angle to the skin
- Steadily inject the full dose (0.5mL) into the tissue

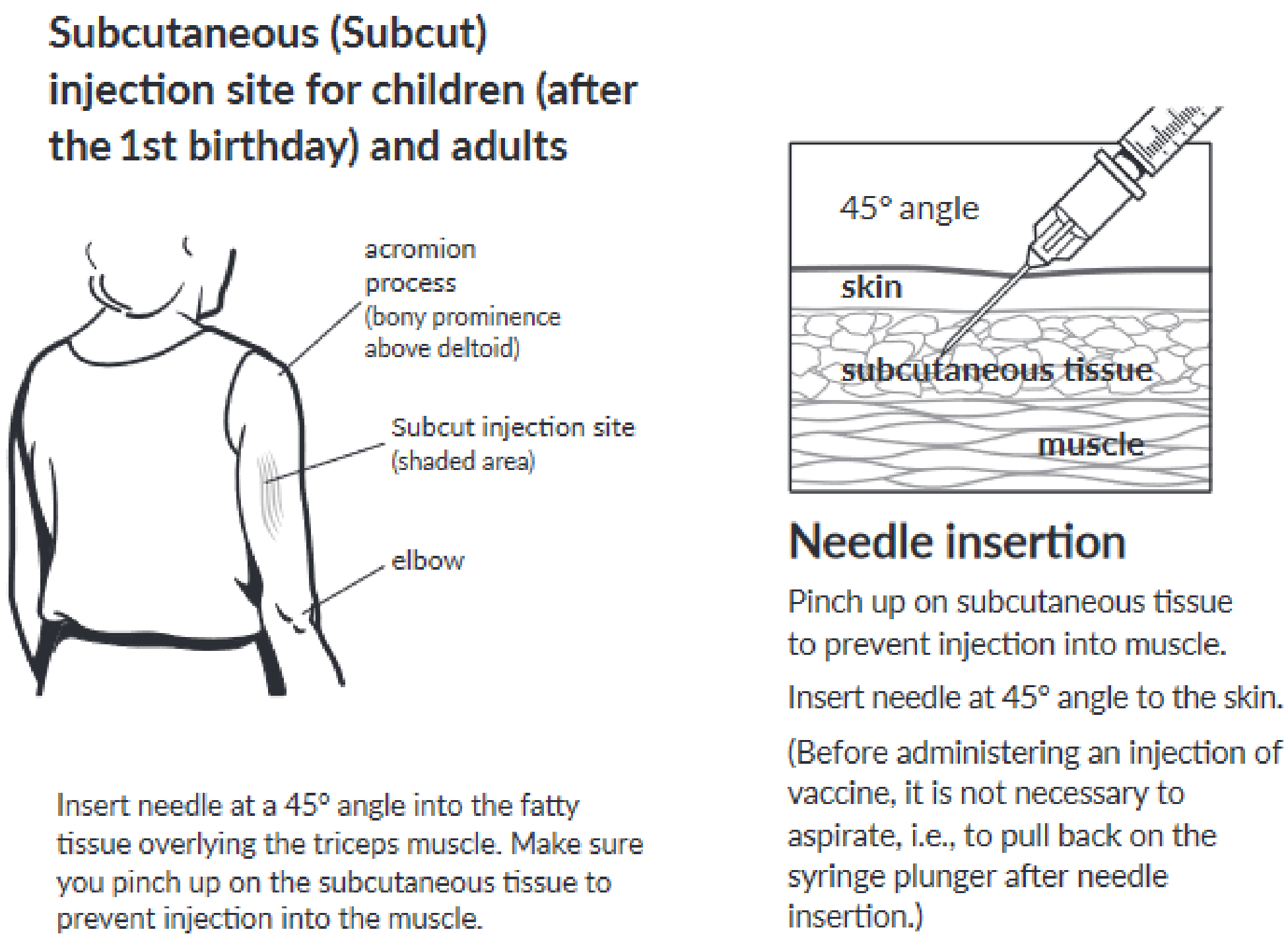


<u>Incorrect administration</u>

If the subcutaneous injection is not performed correctly, some vaccine could be delivered intramuscularly. This does not pose any risk to the participant as the STAMARIL vaccine is also

approved for intramuscular administration (though subcutaneous injection is the recommended route).

As the YF vaccine is supplied in single-dose vials, there is negligible risk of over-dosing. Under dosing may occur if the full volume of the vaccine is not drawn up into the syringe appropriately, or if the full volume is not injected. While this may potentially impact the degree of immune response to vaccination, this does not present any risk to the participant.

Any issues with the vaccination will be recorded in REDCap database (see attachment 15).

Complications

Any serious adverse event or adverse event of interest occurring during the administration of the vaccine or the 20 minutes post administration will be documented appropriately according to the safety monitoring and reporting section of this protocol.

## 6.8 Product accountability

Accountability of antibiotics including documentation of storage, dispensation and destruction (if required) will be maintained in the clinical trial pharmacy files at the RAH. A pharmacy summary/manual will outline the specific processes and regulations.

Accountability of BCG and YF vaccine including documentation of storage, dispensation and destruction (if required) will be maintained in the files of the SA TB Services Clinic (as per their usual clinical processes). A summary/manual will outline the specific processes and regulations.

Any reason for departure from the expected dispensing regimen will be recorded. At the end of the study, there will be final reconciliation of trial drug received, dispensed, used and returned. Any discrepancies will be investigated, resolved and documented by the study team.

## 6.9 Excluded medications and treatments

BCG and YF vaccinations may be given on the same day of any inactivated or live vaccines from a safety perspective but this isn't desirable from a study scientific perspective and participants should be asked to avoid receiving other vaccines where possible within 2 weeks of vaccination administration as part of this protocol.

Participants currently receiving long term (more than 1 month) treatment with isoniazid, rifampicin, streptomycin and ethambutol are excluded as these antibiotics have activity against *M. bovis*.

# 7 RANDOMISATION AND BLINDING

Once consent has been obtained, and during the enrolment visit, participants will be randomised electronically via REDCap. A computer-generated randomisation schedule using balanced variable blocks will be provided by an independent biostatistician from SAHMRI Biostatistics Unit. Randomisation will be to one of 4 groups (antibiotics + BCG vaccine, antibiotics + placebo, no antibiotics + BCG vaccine, no antibiotics + placebo) with an allocation ratio of 1:1:1:1. Participants will be unblinded to their antibiotic status but blinded to their allocated vaccine. Clinicians and/ or vaccinators and pharmacy staff will be unblinded to ensure the correct intervention is given. All other study staff will be blinded until the end of the study.

# 8 STUDY VISITS AND PROCEDURES

## 8.1 Study timeline

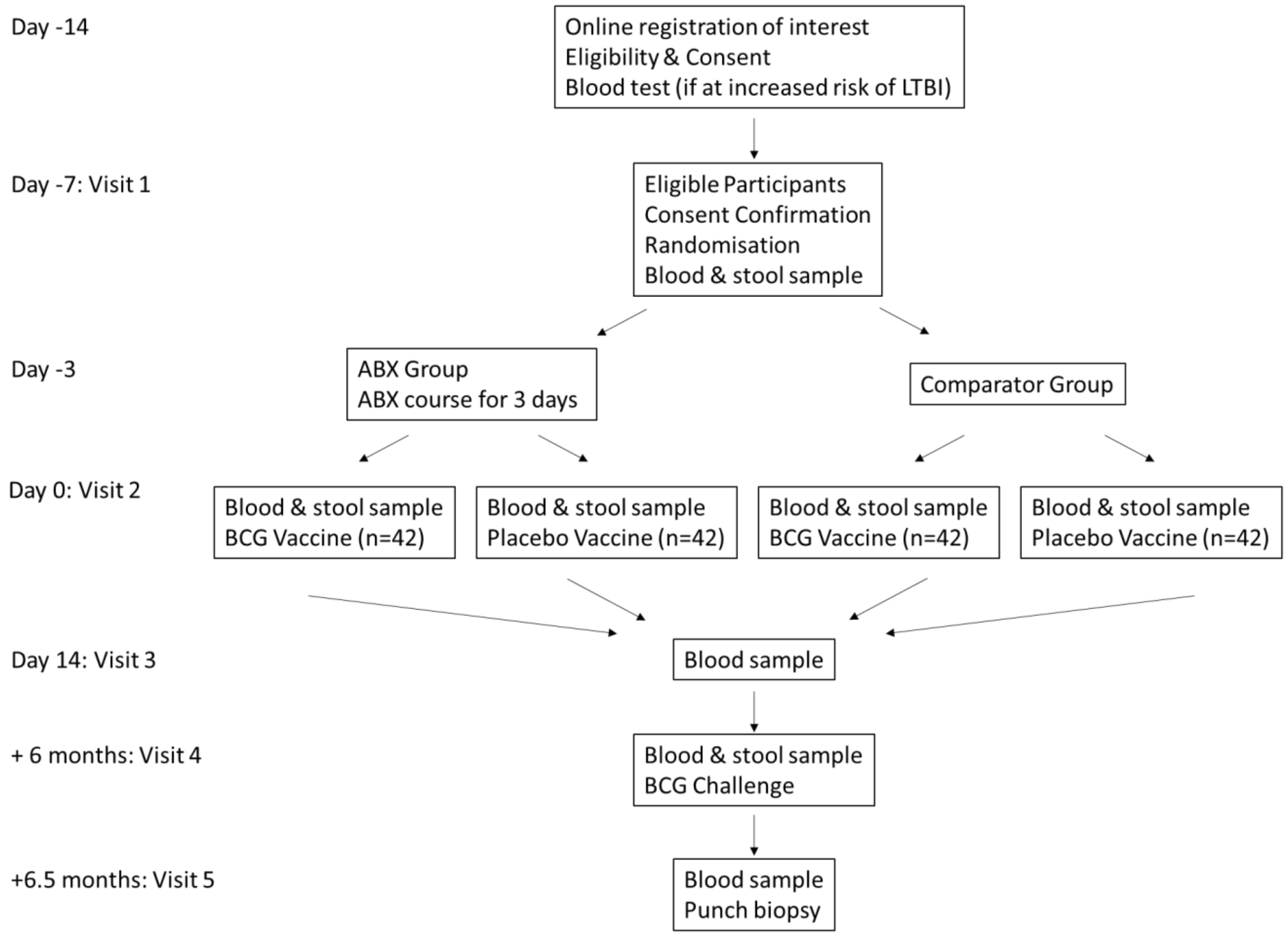


**Figure 5a. Study timeline for BCG rechallenge arm (Arm 1)**

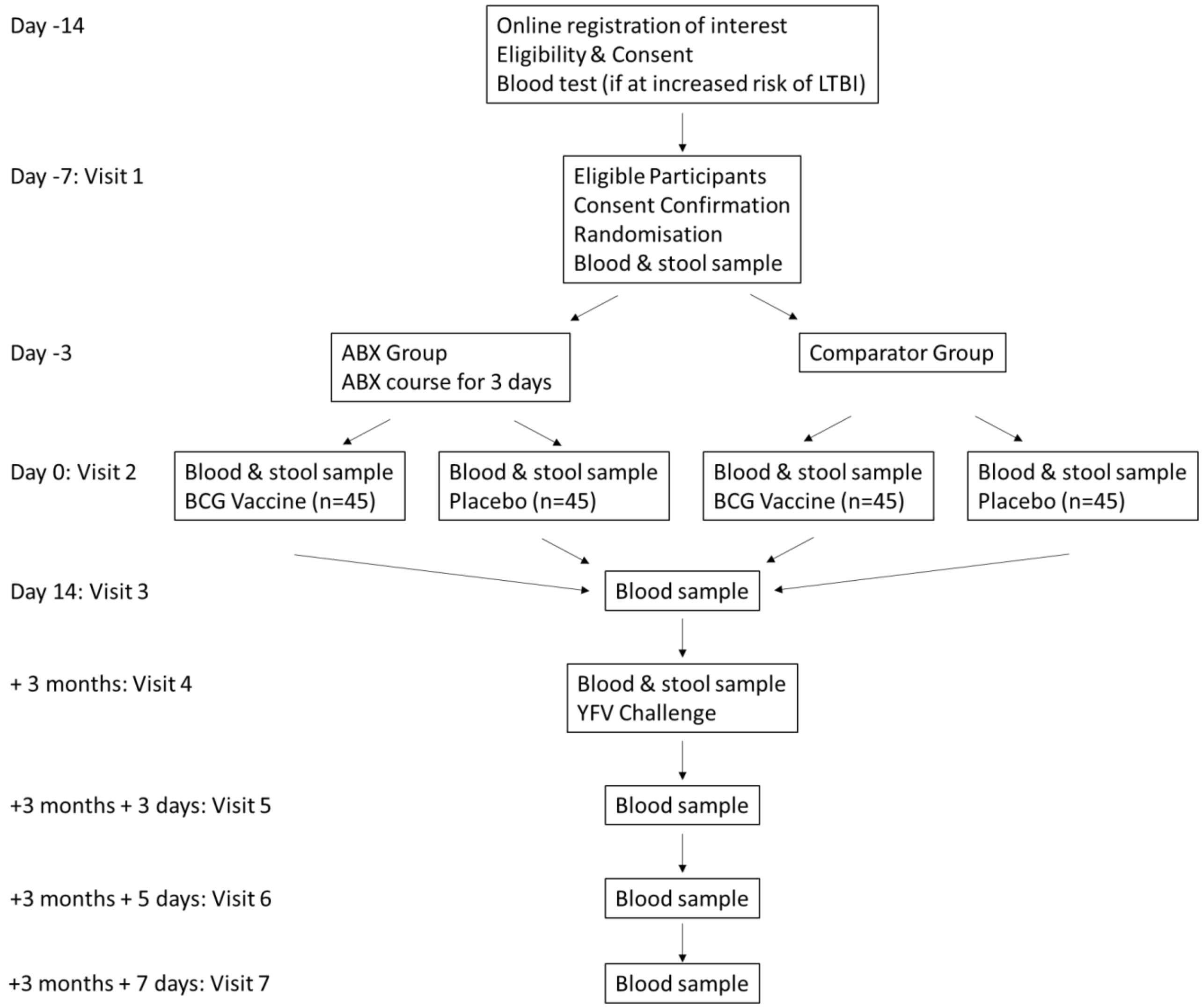


**Figure 5b. Study timeline for YF vaccine arm (Arm 2)**

## 8.2 Schedule of assessments

| Assessments for BCG rechallenge arm | Screening (Remote) Day -14 | Screen Visit (if required) | Visit 1 Day -7 to-3 | Day -3 | Visit 2 Day 0 | Visit 3 Day 14 | Visit 4 6 months | Visit 5 6.5 months |
|---|---|---|---|---|---|---|---|---|
| Eligibility screen: TB screen, demographics | X | | | | | | | |
| Informed consent | X | | X | | | | | |
| IGRA Screen (blood test) | | X | | | | | | |
| Optional pregnancy test | | | X | | | | X | |
| Randomisation (ABX/No ABX and BCG/Placebo) | | | X | | | | | |
| ABX administered | | | X | | | | | |
| Commence ABX | | | | X | | | | |
| BCG or placebo vaccination | | | | | X | | | |
| Photo of vaccination site | | | | | | X | | X |
| BCG challenge | | | | | | | X | |
| BCG punch biopsy | | | | | | | | X |
| Blood sample | | | X | | X | X | X | X |
| Stool sample* | | | X | | X | | X | |
| AE assessment | | | | | X | X | | X |

*Stool sample to be collected by the participant at home, within 24 hours prior to the visit

| Assessments for YF vaccine arm | Screening (Remote) Day -14 | Screen Visit (if required) | Visit 1 Day -7 to-3 | Day -3 | Visit 2 Day 0 | Visit 3 Day 14 | Visit 4 3 months | Visit 5 +3 days | Visit 6 +5 days | Visit 7 +7 days |
|---|---|---|---|---|---|---|---|---|---|---|
| Eligibility screen: TB screen, demographics | X | | | | | | | | | |
| Informed consent | X | | X | | | | | | | |
| IGRA Screen (blood test) | | X | | | | | | | | |
| Optional pregnancy test | | | X | | | | X | | | |
| Randomisation (ABX/No ABX and BCG/Placebo) | | | X | | | | | | | |
| ABX administered | | | X | | | | | | | |
| Commence ABX | | | | X | | | | | | |
| BCG or placebo vaccination | | | | | X | | | | | |
| Photo of vaccination site | | | | | | X | | | | |
| YF challenge | | | | | | | X | | | |
| Blood sample | | | X | | X | X | X | X | X | X |
| Stool sample* | | | X | | X | | X | | | |
| AE assessment | | | | | X | X | | | | X |

*Stool sample to be collected by the participant at home, within 24 hours prior to the visit

## 8.3 Description of procedures

The procedures related to recruitment, consent, eligibility, randomisation and intervention have been described in sections 5-7. SAHMRI will be responsible for the storage and analysing of the samples. The time, date and details of sample collections, including any issues, will be entered in the participants' study record in REDCap database (see attachment 16).

<u>Blood sample collection:</u>

The procedure for blood collection is described in the relevant SOP (SOP-001 see attachment 9). Blood collection will be done by a trained member of the study team at the clinic rooms at SAHMRI or the RAH. After collection, blood samples are processed and stored at SAHMRI.

Note: Extra blood (20ml) will be collected at Visit 1 (Day -7 to -3) and stored, as the many analyses performed on the subsequent timepoints are compared to the baseline sample.

Blood sample analysis:

Samples will be processed and stored at SAHMRI for subsequent analysis of the immune system. The immune system will be assessed by several methods, including:

- measurement of antibodies to BCG vaccination (to determine seroconversion and antibody titres)
- characterisation of immune cell subpopulations
- measurement of immune cell activation and differentiation
- measurement of gene expression responses to vaccination and following antibiotic treatment
- measurement of viremia (viral copies in the blood) after Yellow Fever vaccination
- measurement of immune cell function (e.g. cytokine production and cell division) following *in vitro* stimulation with *mTB* antigens or viral, bacterial and fungal antigens
- measurement of epigenetic and metabolic status of immune cell subsets

Stool sample collection:

Participants will be given two kits to take home at the enrolment visit prior to collection containing instructions of how to collect stool samples (see attachment 10). Participants will be instructed when to collect the samples and following collection can bring the sample to their next visit or mail it to SAHMRI. For the last stool collection, stool kits will be mailed to participants for them to collect a sample in the 48h prior to study visit 4 (BCG challenge or YF vaccination visit). After collection, stool samples are stored at SAHMRI.

Stool sample analysis:

Stool samples will be processed to extract microbial DNA for analysis of the gut microbiota. From the samples, extracted DNA will be used for:

- 16s rRNA gene qPCR to determine bacterial load
- Shotgun metagenomic sequencing or 16S rRNA gene sequencing to profile the composition and encoded functional capacity of the microbiota

From these data, we will determine the degree of microbiome depletion associated with antibiotics treatment at Day 0, and whether antibiotics treatment is associated with altered composition of the microbiota at 6 months.

Punch biopsy collection:

The procedure for the punch biopsy at the BCG vaccination site is described in SOP-003 (see attachment 17). This procedure will be performed in the pilot study and BCG rechallenge arm and will be performed by a clinician trained in the technique.

Punch biopsy analysis:

Following collection, punch biopsy specimens will be bisected and snap-frozen and stored at SAHMRI, in liquid nitrogen prior to analysis. These samples will be used for:

- Culture on solid Middlebrook 7H11 agar to quantify live BCG bacterial load (as colony-forming units)
- DNA extraction for assessment of BCG bacterial load via qPCR or qRT-PCR
- Sectioning and spatial transcriptomics to assess gene expression in a spatially-resolved manner

**Pilot study:** For optimisation of laboratory techniques, especially the spatial transcriptomics methods, which are very novel, a pilot study in up to 10 additional participants will be performed. These participants will give informed consent and have a skin punch biopsy only with no other interventions.

Participants will be recruited (institutional noticeboards/emails) and provided a consent form via email (attachment 18: Pilot Study Participant Information and Consent Form). Following this, they will attend one visit to SAHMRI clinic rooms to have the punch biopsy performed by medical staff. A follow up visit ~7 days later may be necessary if suture removal is required.

These participants will not undergo any other procedures as part of their involvement and no additional personal information will be collected or stored.

# 9 SAFETY MONITORING AND REPORTING

## 9.1 Definitions

Adverse Event (AE):

An AE is any untoward medical occurrence in a participant administered an investigational product and does not necessarily have a causal relationship with the study treatment. For this study, only certain adverse events are recorded, specifically serious adverse events as defined below, and non-serious adverse events of interest specified in section 9.2.

Serious Adverse Event (SAE):

Any serious adverse event (SAE) is an untoward medical occurrence that:

- Results in death; or
- Is life-threatening; or
- Requires hospitalisation or prolongation of existing hospitalisation;
    - Hospitalisation is to be considered an SAE only in the event of an overnight admission. Any elective hospitalisation does not constitute an SAE
- Results in persistent or significant disability/incapacity; or
- Is a congenital anomaly/birth defect

Note: Life-threatening refers to an event in which the participant was at risk of death at the time of the event. It does not refer to an event that hypothetically might have caused death if it were more severe.

Medical and scientific judgement should be exercised in deciding whether an adverse event should be classified as serious in other situations. Important medical events that are not immediately life-threatening or do not result in death or hospitalisation but may jeopardise the participant or may require intervention to prevent one of the other outcomes listed in this definition should also be considered serious.

For this study, all SAE will be collected for the duration of the study.

Suspected Unexpected Serious Adverse Reaction (SUSAR):

A SUSAR is an AE that meets all of the following criteria:

- The AE is serious (as defined above; an SAE); and
- The SAE is suspected adverse reaction to the investigational product, meaning it is judged by either the reporting investigator or the DSMB as having a reasonable possibility of a causal relationship to a study vaccine (possibly, probably or definitely related), and
- The SAE is also unexpected: An unexpected serious adverse reaction is one for which the nature or severity of the reaction is not consistent with reference safety information (Which is comprised of the BCG vaccine Product Information and the *WHO information sheet: Observed rate of vaccine reactions Bacille Calmette Guerin Vaccine April 2012* and the product information sheets for Vancomycin and Neomycin).
- Note: An event is instead considered 'expected' if it is listed in the Reference Safety Information and therefore cannot meet the definition of SUSAR.

Significant Safety Issue (SSI):

A significant safety issue is an issue that could adversely affect the safety of participants or materially impact on the continued ethical acceptability or conduct of the trial.

Urgent Safety Measure (USM):

A measure required to be taken in order to eliminate an immediate hazard to a participant's health or safety. Note: This is a type of significant safety issue that can be instigated by either the investigator or medical monitor and can be implemented before seeking approval from HRECs or institutions.

## 9.2 Capturing adverse event information

For the period of randomisation to completion of the study, the following are non-serious and serious adverse events of interest for this study:

- Gastrointestinal effects following antibiotic use (nausea, abdominal pain, diarrhoea or vomiting)
- At BCG/placebo injection site:
    - Reaction (pain, tenderness, redness, swelling) of grade 3 (severe) or 4 (potentially life threatening)
    - Abscess
    - Large ulcer (>1.5 cm diameter)
    - Keloid scar
    - Unusual local reaction
    - Lymphadenopathy (in region of injection site)
- BCG osteitis/osteomyelitis
- Disseminated BCG infection (BCG-osis)
- At Yellow Fever injection site:
    - Reaction (pain, tenderness, redness, swelling) of grade 3 (severe) or 4 (potentially life threatening)
    - Abscess

    - Lymphadenopathy (in region of injection site)
- Allergic reaction due to BCG, YF vaccine or antibiotic administration
- Fainting episode, seizures and convulsions following antibiotic, vaccination or punch biopsy administration
- At the punch biopsy site:
    - Reaction (prolonged bleeding, pain, swelling, bruising, numbness) of grade 3 (severe) or 4 (potentially life threatening)
    - Infection requiring treatment with antibiotics
    - Allergic reaction to local anaesthetic or dressings
    - Any scar at the biopsy site

Participants will be sent the modified Structured Assessment of Gastrointestinal Symptoms (SAGIS)[40] survey via REDCap to record any AE or SAE during and after antibiotic administration (see attachment 19). Any participants who note at least one severe or very severe AE on SAGIS, or two or more moderate AEs will be sent an additional survey 48 hours after to ask if the AE has resolved. If the participant confirms that the AE has not resolved, they will be referred to the clinical PI and medical monitor to be contacted directly via telephone to ensure an appropriate medical response is enacted.

Participants will be sent a survey via REDCap to record any AE or SAE 7 and 21 days after vaccination (see attachment 20 and 21) and 28 days after punch biopsy (see attachment 24). Participants will also be given a phone number and email address at their screening visit to be able to contact the study coordinator between visits if they need to report non-serious AE or SAE beforehand. The study coordinator will forward any (S)AE reports to the PIs and medical monitor.

## 9.3 Documentation of AEs

For the purposes of this study the medical monitor is responsible for recording the applicable Adverse Events, regardless of their relationship to study vaccines or other interventions.

The documentation of each applicable AE on the REDCap CRF (see attachment 22) will include:

- A description of the AE
- The onset date, duration, date of resolution
- Severity
- Seriousness (SAE or not)
- Any action taken (e.g. treatment, follow-up tests)
- The outcome (recovery, death, continuing)
- The likelihood of the relationship of the AE to the trial treatment

All AEs will be followed to resolution or stabilisation, where possible.

<u>Assessing the relatedness (causality) of a participant's AE:</u>

All non-serious AE and SAE must be assessed for their relationship to the study intervention by medical monitor. The degree of certainty about causality will be graded using the categories below.

| Code | Causal Relationship | Description |
|---|---|---|
| 1 | Unrelated | The AE is clearly NOT related to the intervention |
| 2 | Unlikely | The AE is doubtfully related to the invention |
| 3 | Possible | The AE may be related to the invention |
| 4 | Probable | The AE is likely related to the intervention |

| | | |
|---|---|---|
| 5 | Definite | The AE is clearly related to the intervention |

Assessing the severity of a participant's AE:

The medical monitor is responsible for assessing the severity of the AE. The determination of severity for all AE should be made by the medical monitor based upon medical judgment and the severity categories of Grade 1 to 5 as defined in the first table below, with the following exceptions: injection site pain, redness, tenderness and swelling/induration are assigned severity grades using the specific toxicity grade specified in the vaccine grading toxicity scale below.

| Grade | Severity | Description |
|---|---|---|
| Grade 1 | Mild | Asymptomatic or mild symptoms; clinical or diagnostic observations only; intervention not indicated |
| Grade 2 | Moderate | Moderate; minimal, local or non-invasive intervention indicated; limiting age appropriate instrumental activities of daily living (ADL) |
| Grade 3 | Severe | Severe or medically significant but not immediately life-threatening; hospitalisation or prolongation of hospitalisation indicated; disabling; limiting self-care ADL |
| Grade 4 | Life Threatening | Life-threatening consequences; urgent intervention indicated |
| Grade 5 | Fatal | Death related to AE |

Antibiotic Adverse Event Grading Scale

Reactions to antibiotic administration will be monitored by modified SAGIS for symptoms and severity via REDCap survey:

Have you experienced any new adverse effects relating to the stomach, intestine, bowels or digestion?

- Nausea or feel like you want to vomit Y/N
- Vomiting Y/N
- Indigestion/ Heartburn Y/N
- Bloating Y/N
- Abdominal pain or cramping Y/N
- Increase stomach rumbling Y/N
- Diarrhoea Y/N
- Constipation Y/N
- Increased flatulence Y/N
- Mouth irritation Y/N
- Rectal irritation Y/N
- Faecal Incontinence Y/N
- Other – please describe:

For each 'Yes' answer, please grade the severity of the symptom.

| Grade | Severity | Description |
|---|---|---|
| Grade 1 | Mild | Can be ignored when you do not think about it |
| Grade 2 | Moderate | Cannot be ignored, but does not influence daily activities |
| Grade 3 | Severe | Influencing your concentration on daily activities |
| Grade 4 | Very Severe | Markedly influences your daily activities and/ or requires rest |

Vaccine Toxicity grading scale:

Local reactions to vaccinations are monitored using a toxicity grading scale (Food and Drug Administration 2007)

| Local reaction | Grade 0<br>None | Grade 1<br>Mild | Grade 2<br>Moderate | Grade 3<br>Severe | Grade 4<br>Potentially life threatening |
|---|---|---|---|---|---|
| Pain | None | Does not interfere with activity | Repeated use of nonnarcotic pain reliever > 24 hours or interferes with activity | Any use of narcotic pain reliever or prevents daily activity | Emergency room visit or hospitalisation |
| Redness | None | 2.5 - 5 cm | 5.1 - 10 cm | >10 cm | Necrosis or exfoliative dermatitis |
| Tenderness | None | Mild discomfort to touch | Discomfort with movement | Significant discomfort at rest | Emergency room visit or hospitalisation |
| Swelling / induration | None | 2.5 - 5 cm and does not interfere with activity | 5.1 - 10 cm or interferes with activity | >10 cm or prevents daily activity | Necrosis |
| Itch | None | Itching localised to injection site that is relieved spontaneously or in <48 hours of treatment | Itching beyond the injection site that is not generalised OR<br>Itching localised to injection site requiring ≥48 hours of treatment | Generalised itching causing inability to perform usual social & functional activities | Not applicable |

Food and Drug Administration. (2007). "Guidance for Industry: toxicity grading scale for healthy adult and adolescent volunteers enrolled in preventive vaccine clinical"
Retrieved 08.04.2020, from
https://www.fda.gov/downloads/BiologicsBloodVaccines/GuidanceComplianceRegulatoryInformation/Guidances/Vaccines/ucm091977.pdf

## 9.4 Reporting of safety events

The PI (or delegate) is responsible reporting all SSIs to applicable regulatory authorities and HREC as required in a timely manner. The PI or delegate will use continuous vigilance to identify and report all SSIs to all approving HRECs and the relevant Research Governance Officers. SSIs that meet the definition of an urgent safety measure will be notified within 72 hours, and all other significant safety issues will be notified within 15 calendar days of the PI instigating or being made aware of the issue. The PI will report all SUSARs within 72 hours of identification of the event to the relevant Research Governance Officers.

A DSMB of 3 clinical experts, independent of the study, has also been formed to review the safety of the study (see attachment 23: AVIRS DSMB Charter). The DSMB will meet quarterly to review the progression of the study to maintain the integrity of the study. They will also ensure the safety of the participants by reviewing the AE.

# 10 DATA AND INFORMATION MANAGEMENT

## 10.1 Overview

The PI is responsible for maintaining adequate and accurate files of any relevant source documents that include observations or other data relating to participants. Source data will be attributable, legible (including any changes or corrections), contemporaneous, original, accurate, complete, consistent, enduring and available. Changes to source data (hardcopy and electronic) must be traceable, must not obscure the original entry, and must be explained where this is necessary.

The PI will also maintain accurate case report forms (CRFs) (i.e. the data collection forms) where applicable and be responsible for ensuring that the collected and reported data is accurate, legible, complete, entered in a timely manner and enduring. To maintain the integrity of the data, any changes to data (hardcopy and electronic) must be traceable, must not obscure the original entry, and must be explained where this is necessary.

Any person delegated to collect data or perform data entry will be recorded on the delegation log and will be trained to perform these study-related duties and functions.

## 10.2 Data management

Participant information will be deidentified and given a unique participant code. Data will be entered into a password protected REDCap database located on a SAHMRI server. The Study Coordinator will be responsible for identifiable data, maintaining an enrolment log with the participant information and unique participant code. Other study staff (Site PI, medical staff and medical monitor) who interact with participants will have access to identifying data via REDCap. SAHMRI laboratory staff will receive only deidentified data and samples (only identified by the participant code).

SAHMRI and CALHN have existing security systems, policies and procedures in place as well as software barriers to protect information and ensure confidentiality. Re-identification of data, using

the enrolment log, would only happen if there were participant safety concerns. This would only occur after seeking HREC approval.

Data will be stored securely for at least 15 years from the completion of the study. The CPI is responsible for ensuring that both hardcopy and electronic data are destroyed appropriately. Hardcopy data will be destroyed using confidential bins and electronic data will be deleted, including any backups.

### 10.3 Sample management

Blood, faecal and punch biopsy (skin) samples will be stored, labelled with participant code. No identifying information will be kept with the samples. Samples will be frozen and stored in SAHMRI Level 5 Precision Medicine laboratories (require swipe access) and SAHMRI Cryogenics Facility with access only to the research team and cryogenics staff. They will be stored for an indefinite period of time.

The deidentified samples/data may be sent to ethically approved locations in Australia or overseas for certain analyses or for future research related to infectious diseases, immunology, or vaccines. Participants will be asked for optional consent for their deidentified samples/data to be used for other research studies, and samples/data will only be used for such purposes if the participant has given consent.

If a participant does not consent to future use of their deidentified samples, their samples will be destroyed at the end of the study. Samples will be put into biohazard bins and destroyed by autoclaving or incineration.

## 11 STATISTICAL METHODS

### 11.1 Sample size estimation

<u>BCG Re-vaccination arm:</u>

In a previous study, prior BCG vaccination led to a 6-fold reduction in bacterial load at the challenge site following secondary BCG challenge compared to placebo[24]. Using these data, our power calculation estimates that n=38/group is sufficient to detect a 2-fold reduction in bacterial load in antibiotic treated individuals with 95% power (two-tailed Wilcoxon). Therefore, we will recruit n=42 per arm (total n=168 participants) to allow up to 10% lost to follow-up. An additional 10 participants will be recruited to this arm for the pilot skin punch biopsy study.

<u>Yellow Fever Vaccination arm:</u>

Arts *et al.*[17] previously demonstrated that YFV viremia at 5 days following administration was significantly reduced in BCG vaccinated individuals (mean=60.1, std. dev=80 units) compared to placebo vaccinated individuals (mean=241.1, std. dev=415 units). Given these data, we estimate that n=41/group is sufficient to detect a 2-fold difference in viremia between antibiotic-treated and untreated individuals with 80% power. We will recruit n=45/arm to allow 10% loss to follow-up.

### 11.2 Methods of analysis

The following primary and secondary endpoints will be analysed by a biostatistician from the SAHMRI Biostatistics Unit using linear regression models:

Arm 1:

- Bacterial load (CFU) at the biopsy challenge site
- T cell responses to mTB stimulation (Frequency AIM+ or IFNγ-producing $CD4^+$ T cells)
- Antibody responses to BCG assessed via ELISA

Arm 2:

- Viraemia (viral copies/ml blood) at day 3-7 following YF vaccine challenge
- Cytokine responses following *in vitro* stimulation

Models will include effects for antibiotics (yes or no), vaccine (BCG or placebo) and their interaction, with adjustment for the stratification variable (sex). Differences in means and 95% confidence intervals will be reported for the 3 pairwise comparisons that are of interest a priori (vaccinated ABX vs vaccinated no ABX; vaccinated no ABX vs placebo no ABX; vaccinated ABX vs placebo ABX). Interaction p-values will also be reported. All analyses of primary and secondary endpoints will follow a pre-specified statistical analysis plan.

Shotgun metagenomics data will be analysed using an established pipeline. Briefly, data will be input into the HUMAnN 3.0 tool[41] for taxonomic, strain-level, functional, and phylogenetic profiling of metagenomes and to determine relative abundance of bacterial species within samples. Alpha and beta diversity will be assessed between samples using PhyloSeq v1.28[42]. Spearman correlation will be used to correlate relative abundance of specific species with other datasets (skin bacterial load, T cell responses, RNAseq).

RNAseq analysis of peripheral blood will be performed using gene set enrichment analysis, following established methodology[43].

Spatial transcriptomics for punch biopsy samples will be performed using a 10X Xenium system (or more advanced system if available), a hybridisation-based system that assesses expression of hundreds of defined targets in a spatially-resolved manner across a tissue sample. This will enable visualisation of what cells are present in the sample and their gene expression profile. Analysis techniques for this new platform are still evolving, however similar techniques have been applied by the Lynn Systems Immunology group and data analysis will be conducted by a dedicated specialist in spatial transcriptomics together with the South Australian Genomics Centre. Given the expense of this technique, analysis will be performed on tissue from participants defined as high and low responders (6 participants per group) via bacterial load and blood transcriptomic analysis.

# 12 ETHICS AND DISSEMINATION

## 12.1 Research ethics approval and local governance authorisation

The study will be conducted in full conformance with principles of the “Declaration of Helsinki”, Good Clinical Practice (GCP), the National Statement on Ethical Conduct in Human Research (NHMRC, 2007), Australian Code for the Responsible Conduct of Research (2018) and within the laws and regulations Australia.

The protocol, informed consent form and supporting documents will be reviewed and approved by CALHN HREC, prior to implementation. Approval from SAHMRI and CAHLN governance will be sought before implementation.

## 12.2 Benefits and risks

There are no direct benefits and limited risk to participants. However, understanding the effects that poor gut health has on immune responses to vaccination could be of benefit to vulnerable populations by improving any poor response to vaccinations. This benefit of added protection from infectious diseases for vulnerable populations, outweighs the limited risk to participants.

## 12.3 Amendments to the protocol

Any changes or additions to the protocol or informed consent form will be submitted to HREC for approval, prior to implementation.

## 12.4 Protocol deviations

A protocol deviation is any noncompliance with the study protocol, GCP, or HREC requirements. The noncompliance may be either on the part of the participant, the PI, or the study staff. As a result of deviations, corrective actions are to be implemented promptly.

The PI will be notified within 72 hours once a protocol deviation is identified and reported. All deviations are recorded and reported to the relevant regulatory authorities and HREC.

## 12.5 Declaration of interests

There are no conflicts of interest to declare.

## 12.6 Dissemination plan

Results will be published in a peer-reviewed journal. De-identified metagenomics, transcriptomics, epigenomics and other related immunological data accompanying the publication will be deposited in an appropriate international repository, in line with the editorial policies of the publisher.

# 13 PARTICIPANT REIMBURSEMENT

Arm 1: A pro-rated reimbursement of $750 is given to the participants in the BCG rechallenge arm on completion of the study (a lesser amount is given if the participant withdraws from the study early). Payments are given after Visit 3 and 5.

| Visit | Procedures included | AU$ |
|---|---|---|
| Screening visit 1 | Consent, eligibility confirmed, ABX and blood sample | |
| Visit 2 | Vaccination, blood and stool sample | |
| Visit 3 | Blood and stool sample | $200 |
| Visit 4 | Vaccination, blood sample | |
| Visit 5 | Biopsy, blood and stool sample | $550 |

Arm 2: A pro-rated reimbursement of $500 is given to the participants in the YF vaccine arm on completion of the study (a lesser amount is given if the participant withdraws from the study early). Payments are given after Visits 3 and 7.

| Visit | Procedures included | AU$ |
|---|---|---|
| Screening visit 1 | Consent, eligibility confirmed, ABX and blood sample | |
| Visit 2 | Vaccination, blood and stool sample | |
| Visit 3 | Blood and stool sample | $200 |
| Visit 4 | Vaccination, blood sample | |
| Visit 5 | Blood and stool sample | |

| | | |
|---|---|---|
| Visit 6 | Blood sample | |
| Visit 7 | Blood sample | $300 |

# 14 REFERENCES


**1** Lynn, D.J., Benson, S.C., Lynn, M.A. & Pulendran, B. Modulation of immune responses to vaccination by the microbiota: implications and potential mechanisms. *Nature Reviews Immunology* 22, 33-46 (2022).

**2** Dockrell, H.M. and E. Butkeviciute, *Can what have we learnt about BCG vaccination in the last 20 years help us to design a better tuberculosis vaccine? Vaccine*, 2022. 40(11): p. 1525-1533.

**3** Lynn, M.*, et al.* Lynn D.J. Early-Life Antibiotic-Driven Dysbiosis Leads to Dysregulated Vaccine Immune Responses in Mice. *Cell host & microbe* 23, 653-660 (2018).

**4** Aaby, P.*, et al.* Lynn D.J., Shann F. The non-specific and sex-differential effects of vaccines. *Nature Reviews Immunology* 20, 464-470 (2020).

**5** Andre, F.*, et al.* Vaccination greatly reduces disease, disability, death and inequity worldwide. *Bulletin of the World Health Organization* 86, 140-146 (2008).

**6** Lewnard, J., et al. Childhood vaccines and antibiotic use in low- and middle-income countries. *Nature* 581, 94-99 (2020).

**7** Clark, A.*, et al.* Efficacy of live oral rotavirus vaccines by duration of follow-up: a meta-regression of randomised controlled trials. *Lancet Infect. Dis.* 19, 717-727 (2019).

**8** DeJong, E., Surette, M., Bowdish, D. The Gut Microbiota and Unhealthy Aging: Disentangling Cause from Consequence. *Cell Host & Microbe* 28, 180-189 (2020).

**9** Backhed, F.*, et al.* Dynamics and Stabilization of the Human Gut Microbiome during the First Year of Life. *Cell Host & Microbe* 17, 690-703 (2015).

**10** Pasolli, E.*, et al.* Extensive Unexplored Human Microbiome Diversity Revealed by Over 150,000 Genomes from Metagenomes Spanning Age, Geography, and Lifestyle. *Cell* 176, 649-662 e620 (2019).

**11** Harris, V.*, et al.* Effect of Antibiotic-Mediated Microbiome Modulation on Rotavirus Vaccine Immunogenicity: A Human, Randomized-Control Proof-of-Concept Trial. *Cell Host & Microbe* 24, 197-207 (2018).

**12** Hagan, T.*, et al.* Antibiotics-Driven Gut Microbiome Perturbation Alters Immunity to Vaccines in Humans. *Cell* 178, 1313-1328 (2019).

**13** Ng, S.*, et al.* Gut microbiota composition is associated with SARS-CoV-2 vaccine immunogenicity and adverse events. *Gut* (2022).

**14** Higgins, J.*, et al.* Association of BCG, DTP, and measles containing vaccines with childhood mortality: systematic review. *BMJ* **355**, i5170 (2016).

**15** Aaby, P.*, et al.* Randomized trial of BCG vaccination at birth to low-birth-weight children: beneficial nonspecific effects in the neonatal period? *J Infect Dis* **204**, 245-252 (2011).

**16** Giamarellos-Bourboulis, E.*, et al.* Activate: Randomized Clinical Trial of BCG Vaccination against Infection in the Elderly. *Cell* **183**, 315-323 e319 (2020).

**17** Arts, R.*, et al.* BCG Vaccination Protects against Experimental Viral Infection in Humans through the Induction of Cytokines Associated with Trained Immunity. *Cell Host & Microbe* **23**, 89-100 e105 (2018).

**18** Kaufmann, E., et al. BCG Educates Hematopoietic Stem Cells to Generate Protective Innate Immunity against Tuberculosis. *Cell* **172**, 176-190 e119 (2018).

**19** Mitroulis, I., et al. Modulation of Myelopoiesis Progenitors Is an Integral Component of Trained Immunity. *Cell* **172**, 147-161 e112 (2018).

**20** Kleinnijenhuis, J., et al. Long-lasting effects of BCG vaccination on both heterologous Th1/Th17 responses and innate trained immunity. *Journal of Innate Immunity* **6**, 152-158 (2014).

**21** Strazar, M., et al. The influence of the gut microbiome on BCG-induced trained immunity. *Genome biology* **22**, 275 (2021).

**22** World Health Organisation. Global tuberculosis report 2021. Geneva; 2021.

**23** Pittet, L*, et al.* BCG vaccination to reduce the impact of COVID-19 in healthcare workers: Protocol for a randomised controlled trial (BRACE trial). *BMJ Open* 11, e052101 (2021).

**24** Minhinnick, A., *et al.* Optimization of a Human Bacille Calmette-Guerin Challenge Model: A Tool to Evaluate Antimycobacterial Immunity. *J Infect Dis* 213, 824-830 (2016).

**25** Brook, B., *et al.* BCG vaccination-induced emergency granulopoiesis provides rapid protection from neonatal sepsis. *Sci Transl Med* **12**(2020).

**26** Baruch, E., *et al.* Fecal microbiota transplant promotes response in immunotherapy-refractory melanoma patients. *Science* 371, 602-609 (2021).

**27** Australian Technical Advisory Group on Immunisation (ATAGI). Australian Immunisation Handbook. Canberra: Australian Government Department of Health,; 2018.

**28** Lotte A, Wasz-Hockert O, Poisson N, Dumitrescu N, Verron M, Couvet E. BCG complications. Estimates of the risks among vaccinated subjects and statistical analysis of their main characteristics. Adv Tuberc Res. 1984;21:107-93.

**29** Hendry AJ, Dey A, Beard FH, Khandaker G, Hill R, Macartney KK. Adverse events following immunisation with bacille Calmette-Guerin vaccination: baseline data to inform monitoring in Australia following introduction of new unregistered BCG vaccine. Communicable diseases intelligence quarterly report. 2016;40:E470-e4.

**30** Australian Government Department of Health. The BCG vaccine: information and recommendations for use in Australia March 2013.

**31** BCG Vaccine SSI data sheet 14 March 2018. Ltd SN, [https://www.medsafe.govt.nz/profs/datasheet/b/BCGCSLinj.pdf, accessed 24 Dec 2019]

**32** Pilgrim S. Administration of the Bacillus Calmette Guerin (BCG) Vaccination in neonates. 2019.

**33** Coulter C. Tuberculosis testing. Aust Fam Physician. 2012;41:489-92.

**34** Villanueva P, Wadia U, Crawford N, Messina NL, Kollmann TR, Lucas M, *et al*. Revaccination with Bacille Calmette-Guerin (BCG) is associated with an increased risk of abscess and lymphadenopathy. NPJ Vaccines. 2022;7(1):6.

**35** Lindsey, N.P., Rabe, I.B., Miller, E.R., Fischer, M. & Staples, J.E. Adverse event reports following yellow fever vaccination, 2007-13. J Travel Med 23(2016).

**36** Staples, J.E., Gershman, M., Fischer, M., CDC. Yellow fever vaccine: recommendations of the Advisory Committee on Immunization Practices (ACIP). MMWR Recomm Rep 59, 1-27 (2010).

**37** Seligman, S.J. Risk groups for yellow fever vaccine-associated viscerotropic disease (YEL-AVD). Vaccine 32, 5769-5775 (2014).

**38** Tanes C, Bittinger K, Gao Y, Friedman ES, Nessel L, Paladhi UR, *et al*. Role of dietary fiber in the recovery of the human gut microbiome and its metabolome. Cell Host Microbe. 2021;29(3):394-407 e5.

**39** National Health and Medical Research Council. Payment of participants in research: information for researchers, HRECs and other ethics review bodies. Canberra (AU): R41F; 2014.

**40** Koloski, N.A., *et al*. The validity of a new structured assessment of gastrointestinal symptoms scale (SAGIS) for evaluationg symptoms in the clinical setting. Dig Dis Sci.2017; 62:1913-1922.

**41** Franzosa, E.A., *et al., Species-level functional profiling of metagenomes and metatranscriptomes.* Nat Methods, 2018. 15(11): p. 962-968.

**42** McMurdie, P.J. and S. Holmes, phyloseq: an R package for reproducible interactive analysis and graphics of microbiome census data. PLoS One, 2013. 8(4): p. e61217.

**43** Ryan F.J., Lynn DJ., *et al.* Long-term perturbation of the peripheral immune system months after SARS-CoV-2 infection. BMC Med. 2022 Jan 14;20(1):26.

# 15 ATTACHMENTS

1. AVIRS Flyer V2.0
2. AVIRS Participant Communication V3.0
3. AVIRS PICF V3.0
4. AVIRS PICF YF V2.0
5. AVIRS Screening and Consent CRF V4.0
6. AVIRS Screening Blood Test Consent Form V2.0
7. AVIRS IGRA Testing CRF V1.0
8. AVIRS Eligibility and Consent Confirmation CRF V4.0
9. SOP-001 Phlebotomy V2.0
10. AVIRS Stool Instructions to Participants
11. SOP-002 BCG/Placebo Vaccination V2.0
12. SOP-004 BCG Challenge Vaccination for BCG V2.0
13. SOP-005 YF Vaccination V2.0
14. AVIRS What to Expect V2.0
15. AVIRS Vaccination CRF V2.0
16. AVIRS Sample Collection CRF V2.0
17. SOP-003 Punch biopsy sampling
18. AVIRS Pilot PICF V1.0
19. AVIRS ABX Survey CRF V5.0
20. AVIRS BCG Survey CRF V5.0
21. AVIRS YF Survey CRF V2.0
22. AVIRS Medical Monitor Reporting V2.0
23. AVIRS DSMB Charter V1.0
24. AVIRS Punch Biopsy Survey CRF V1.0
25. SOP-006 Management of a Vaso Vagal Response V1.0
