# Supplementary material for: A randomised, placebo-controlled study to assess whether antibiotic-mediated disruption of the gut microbiota impairs BCG-induced specific and non-specific immunity in young, healthy adults: protocol for the Antibiotics and Vaccine Immune Responses Study (AVIRS)
Source: BMJ Open. 2026 Oct 14;16(10):e126924. doi: 10.1136/bmjopen-2026-126924 (PMC13691821; doi:10.1136/bmjopen-2026-126924)
Supplement: Supplementary file 2 [file bmjopen-16-10-s004.pdf]

Form 1

Screening and Consent CRF – PARTICIPANT TO COMPLETE

Eligibility

Date:

Thank you for your interest in AVIRS. This study aims to determine whether depleting the gut bacteria (by taking antibiotics) changes the immune response to the BCG vaccine (used to protect against tuberculosis).

To determine whether you are eligible to participate in this study we would like to ask you a few personal questions including questions about your age, previous vaccinations and medical history. The answers you give will be kept confidential.

If you have any queries about the questions, please contact the AVIRS team 0499 385 609 or by email avirs@sahmri.com

Do you agree to the collection of your personal information for screening purposes?

- **Yes**
- **No**

If answer 'No', 'Thank you for your interest, the screening process will not continue'

First Name: _____________

Last Name: _____________

Email: _____________

Mobile Number: _______________

Please answer these questions check whether you are eligible to participate.

1. Are you between 18 and 35 years old? Date of Birth: ________________
   - No
   - Yes
2. Have you ever had the Yellow Fever vaccine?
   In Australia, the Yellow Fever vaccine is not routinely given as part of the National Immunisation Program. It is however recommended for people aged ≥9 months who are travelling to an area with a risk of yellow fever virus transmission such as tropical and subtropical regions of Africa and South America. If you have travelled to these parts of the world it is likely that you would have been recommended to have this vaccine. See https://immunisationhandbook.health.gov.au/contents/vaccine-preventable-diseases/yellow-fever' for further information.
   - No
   - Yes
   - Unsure
   - *If answer 'unsure' for question 2: 'Have you ever had any vaccinations for the purpose of travelling to Central/ South America or Africa?'*

3. Have you ever had Yellow Fever in the past?
   - No
   - Yes
4. Have you ever had the BCG vaccine for tuberculosis (TB)?
   In Australia, the BCG vaccine has not been routinely given as part of the National Immunisation Program since 1985, however it is widely administered at birth or in early-life in other countries worldwide and in selected circumstances in Australia for individuals with an increased risk of TB exposure. See https://immunisationhandbook.health.gov.au/contents/vaccine-preventable-diseases/tuberculosis' for further information.
   - No
   - Yes
   - Unsure
5. What is your country of birth? ___________________
6. Have you ever knowingly been in close contact with someone who has TB?
   - No
   - Yes
7. Have you ever had TB in the past?
   - No
   - Yes
8. Have you been screened in Australia for TB?
   - No
   - Yes

   If yes, please provide some details about the screening: why you needed to be screened, what screening was done (questionnaire, x-ray, blood test), where the screening was done and what was the result?

9. Have you worked or lived in any of the following countries for periods of more than 3 months? {countries with WHO-defined high TB burden listed}
   - No
   - Yes
10. Have you ever been involved in the care of patients with TB?
    - No
    - Yes
11. Have you had any vaccinations in the last 4 weeks?
    - No
    - Yes
12. Do you have any of the following medical conditions?
    - Chronic renal disease
    - Thymus disease (e.g. myasthenia gravis, thymoma, thymectomy, DiGeorge syndrome)
    - HIV infection
    - Cancer
    - Autoimmune disease
13. Do you have any moderate or severe skin diseases (e.g. eczema, dermatitis, psoriasis etc.)?
    - No
    - Yes

14. Do you have tattoos that **mostly or completely** cover one or both of your upper arms?
    - Yes
    - No
15. Are you currently on antibiotics or have taken any in the last month?
    - No
    - Yes
16. Are you currently taking any of the following?
    - Steroids, by tablet or injection
    - Other medications that suppress the immune system
    - Chemotherapy
    - Other prescription medication
17. Have you ever had Guillian-Barre syndrome?
    - No
    - Yes
18. Have you ever had a serious allergic reaction to a vaccine (e.g. anaphylaxis) or antibiotics?
    - No
    - Yes, please list the reaction
19. Are you currently breastfeeding?
    - No
    - Yes

BCG and Yellow Fever vaccines are live vaccines and NOT recommended during pregnancy

20. Are you pregnant or could you be pregnant or planning to be pregnant in the next 12 months?

(A pregnancy test can be provided as discretely as possible at the screening visit)

- No
- Yes
- Unsure

21. How did you hear about AVIRS? _________________

Is the participant eligible for this study? [Response sent to participant]

- Yes – **You are eligible to participate in AVIRS, a consent form will be sent to you shortly**
- No – **Thank you for your interest, you are ineligible and unable to proceed with the study.**
- No – possible LTBI: **Thank you for your interest in AVIRS. A member of the study team will be in contact with you to arrange further screening for the study.**
- **Uncertain – need further clarification from medical staff: Thank you for your interest in AVIRS. A member of the study team will be in contact with you to arrange further screening for the study.**
