# Supplementary material for: A randomised, placebo-controlled study to assess whether antibiotic-mediated disruption of the gut microbiota impairs BCG-induced specific and non-specific immunity in young, healthy adults: protocol for the Antibiotics and Vaccine Immune Responses Study (AVIRS)
Source: BMJ Open. 2026 Oct 14;16(10):e126924. doi: 10.1136/bmjopen-2026-126924 (PMC13691821; doi:10.1136/bmjopen-2026-126924)
Supplement: Supplementary file 3 [file bmjopen-2026-126924.reviewer_comments.pdf]

PEER REVIEW HISTORY

BMJ Open publishes all reviews undertaken for accepted manuscripts. Reviewers are asked to complete a checklist review form and are provided with free text boxes to elaborate on their assessment. These free text comments are reproduced below.

ARTICLE DETAILS

Title (Provisional)

Protocol for the Antibiotics and Vaccine Immune Responses Study (AVIRS): A randomised, placebo-controlled study to assess whether antibiotic-mediated disruption of the gut microbiota impairs BCG-induced specific and non-specific immunity in young, healthy adults

Authors

Stevens, Natalie; Botten, Rochelle; geake, james; Eden, Georgina L; Lo, Wing Ki; Inauen, Joseph; Solujic, Jelena; Sawka, Alice; Yelland, Lisa N; Reid, Elisabeth W; Shaw, Michelle; Zhu, Xiaodan; Tam, Tsz Kin; Jeon, Hyejin; Amenyogbe, Nelly; Lynn, Miriam Anne; Ryan, Feargal J.; Feng, Carl G; Britton, Warwick J; Kollmann , Tobias; Barry, Simone; Lynn, David J

This article was not externally reviewed at BMJ Open. Protocols that have been independently assessed prior to submission to BMJ Open are usually fast-tracked to publication on the grounds that further substantial changes will not be possible. This independent assessment will usually be external, independent review for both a substantial grant award from a non-commercial or government funder and ethics approval.
