# Supplementary material for: A randomised, placebo-controlled study to assess whether antibiotic-mediated disruption of the gut microbiota impairs BCG-induced specific and non-specific immunity in young, healthy adults: protocol for the Antibiotics and Vaccine Immune Responses Study (AVIRS)
Source: BMJ Open. 2026 Oct 14;16(10):e126924. doi: 10.1136/bmjopen-2026-126924 (PMC13691821; doi:10.1136/bmjopen-2026-126924)
Supplement: Supplementary file 4 [file bmjopen-16-10-s001.docx]

**Participant Information Sheet/Consent Form**

*Adult providing own consent*

| **Title** | A human experimental medicine study to assess whether the gut microbiota regulates specific immune responses to vaccination |
| --- | --- |
| **Short Title** | Antibiotics and Vaccine Immune Responses Study (AVIRS) – BCG Vaccine Arm |
| **Protocol Number** | 2023/HREC00066 |
| **Project Sponsor** | SAHMRI |
| **Co-ordinating Principal Investigator** | Prof David Lynn |
| **RAH Principal Investigator** | Dr Simone Barry |
| **Location** | Adelaide, SA |

**Part 1	What does my participation involve?**

1 **Introduction**

You are invited to participate in Antibiotics and Vaccine Immune Responses Study (AVIRS). This study involves two different arms. In this arm, we would like to assess whether or not depletion of the gut bacteria with antibiotics changes the immune response to the BCG vaccine (used to protect against Tuberculosis).

This Participant Information Sheet/Consent Form tells you about the research study. It explains the tests involved. Knowing what is involved will help you decide if you want to take part in the research.

Please read this information carefully. Ask questions about anything that you don’t understand or want to know more about. Before deciding whether or not to take part, you might want to talk about it with a relative, friend or your local doctor.

Participation in this research is voluntary. If you don’t wish to take part, you don’t have to.

If you decide you want to take part in the research study, you will be asked to sign the consent section. By signing it you are telling us that you:

• Understand what you have read

• Consent to take part in the research study

• Consent to have the tests that are described

• Consent to the use of your personal and health information as described.

You will be given a copy of this Participant Information and Consent Form to keep.

2 **What is the purpose of this research?**

Studies have shown an association between the amount and type of bacteria in the intestines (the gut microbiota) and the effectiveness of some vaccines, indicating that people with healthy gut bacteria may have better immune responses to vaccines than people with altered (poor) gut bacteria. When the bacteria in the gut are reduced, the immune response to vaccination may be muted, resulting in less protection against diseases.

We would like to find out if depletion of the gut bacteria (by using antibiotics) impairs the protection given by the BCG vaccine. We will assess this by comparing people that have taken a short course of antibiotics to people that have not taken antibiotics prior to BCG vaccination.

We also hope to identify which bacteria in the gut are helpful for immune responses to vaccination. We will do this by comparing the types of gut bacteria in people with good and poor responses to vaccination. This information could lead to development of ways to improve immune responses to vaccination, such as probiotics or dietary changes. This may be particularly useful for vulnerable populations that often have poor responses to vaccination, such as babies and the elderly, and give them more protection from infectious diseases.

3 **Who is undertaking this research?**

This research has been initiated by Professor David Lynn, Precision Medicine Theme, The South Australian Health and Medical Research Institute (SAHMRI) and Dr Simone Barry, Head TB Clinic, Royal Adelaide Hospital (RAH).

This study is funded by the National Health and Medical Research Council (NHMRC).

4 **Do I have to take part in this research project?**

This is a research study and you do not have to be involved. Also, you may withdraw from the project at any time after you have commenced. Your participation in this study shall not affect any other right to compensation you may have under common law.

5 **What does participation in this research involve?**

This is a randomised controlled study. We put people into groups and give each group a different treatment. The results are compared to see if one is better. To try to make sure the groups are the same, each participant is put into a group by chance (random). The chance of being in each group is 1 in 2, or 50%.

In this study, we will have 2 randomisations. You will be randomised into one of two groups:

**Antibiotics group** – You will be given antibiotic tablets (neomycin and vancomycin) to take for 3 days

**Comparator group** – You will not have antibiotics

You will then be randomised again into one of two groups:

**Intervention group 1** – You will be given a placebo vaccine via injection into the left arm. A placebo looks like the real thing but contains no active ingredients.

**Intervention group 2** – You will be given the BCG vaccine via injection into the left arm.

We will assess immune responses to vaccination by collecting and analysing blood samples. We will also ask you to provide stool samples during the study (these will be collected at home), so we can assess how well the antibiotics have reduced the gut bacteria.

Six months after vaccination/placebo vaccination, everyone in the study will receive the BCG vaccine (challenge vaccine) in the right arm. As the BCG vaccine contains live, weakened bacteria, we can measure how quickly the bacteria from the BCG vaccine are killed at the injection site, and someone who has immunity to this vaccine from prior vaccination will kill the bacteria faster than someone who has never been vaccinated before. We will measure this by taking a sample (biopsy) of the skin at the challenge vaccination site and counting the live bacteria in it.

If you agree to take part in this study, you will need to attend the clinic 5 times over 7 months at the South Australian Health and Medical Research Institute (SAHMRI) or Royal Adelaide Hospital (RAH) on North Terrace in Adelaide.

The following samples will be collected each time point:

| **Visit 1** | - A blood sample of about 60 ml to be collected - You will be given a stool sample kit to collect two stool samples at home   If you are randomised to receive antibiotics, you will also be given:   - a prescription to take to RAH pharmacy and receive the antibiotic tablets - a diary to record when you took the tablets and any gastrointestinal effects of these |
| --- | --- |
| After 3-14 days  **Visit 2** | - A blood sample of about 40 ml to be collected - You will be given the BCG vaccine OR placebo vaccine in the left arm - You will return the stool samples (collected at home before the visit)   An online survey will be sent via email to record any side effects after vaccination |
| After 2 weeks  **Visit 3** | - A blood sample of about 40 ml to be collected |
| After 6 months  **Visit 4** | - A blood sample of about 40 ml to be collected - You will be given the BCG vaccine in the right arm - You will return the stool sample (collected at home before the visit)   An online survey will be sent via email to record any side effects after vaccination |
| After 2 weeks  **Visit 5** | - A blood sample of about 40 ml to be collected - A small (4mm) punch skin biopsy will be taken in the right arm at the site where the BCG vaccine was administered 2 weeks earlier |

A 40ml blood sample is approximately 2 tablespoons.

6 **What do I have to do?**

If you want to participate in this study, you will need to:

- Be willing to take a 3 day course of antibiotics
- Receive 1-2 BCG vaccinations and complete a survey after each one
- Attend the clinic 5 times and have a blood sample taken each time
- Provide 3 stool samples (to be collected at home)
- Have a small (4mm) skin punch biopsy taken

7 **Will I be reimbursed?**

You will be reimbursed for your time and efforts. You will receive $750 in total for participating in this study. You will receive a small payment after Visit 3 ($200), with a larger payment ($550) at the end.

8 **What are the possible benefits of taking part?**

We cannot guarantee or promise that you will receive any benefits from this trial. You will receive 1-2 doses of the BCG vaccine, which may protect you from getting sick with tuberculosis (this may not be of benefit as it is not likely you will contract tuberculosis in Australia). Results from this study may help people in the future.

9 **What are the possible risks and disadvantages of taking part?**

BCG Vaccine:

BCG is one of the most widely used vaccines in the world with an established safety record. It has been given to people since the 1920s. BCG is used to protect against a bacterial infection of the lungs called tuberculosis. BCG is a ‘live’ vaccine made of attenuated (weakened) bacteria – the type of bacteria in BCG cause tuberculosis in cattle (bovine tuberculosis) but don’t cause disease in humans. BCG is not given routinely in Australia, because Australia has very few cases of tuberculosis. However, it is still used across the world, especially Asia, Africa and Europe.

Most vaccines are injected into muscle, BCG is a little different as it is given just under the skin (into the ‘intradermal’ layer) of the left upper arm. BCG immunisation hurts a little, but this is minimised when given by experienced immunisation staff such as those who will be performing the procedure in this study.

The usual expected reaction to BCG vaccination is redness and/or a small ‘papule’ (a pimple or lump) at the injection site that appears weeks to months after vaccination. A few weeks later, the papule usually softens and breaks down to a small sore/ulcer (usually less than 15 mm in diameter). The sore is painless and may last from weeks to months. Once the sore has healed, this usually (but not always) leaves a small flat scar. Most people in Australia over the age of 50 and any that lived or travelled to a country with high levels of TB as a child, will have this scar.

Having a small sore will not impact your ability to go to work. You can cover it with a bandage during the day if needed.

BCG vaccination can occasionally cause adverse effects, these usually get better by themselves, without requiring any specific treatment. The risk of these reactions is minimised by use of correct immunisation technique by trained staff. You may have none, some, or all of the effects listed below, and they may be mild, moderate or severe. If you have any of these side effects, or are worried about them, contact us.

*Common adverse reactions:*

These reactions are seen in less than 1 in 100 people immunised with BCG and usually resolve without any specific treatment:

- Abscess at the injection site or a larger ulcer
- Keloid scar at injection site (it means ‘a scar thicker than usual’)
- Swelling of local gland (lymph node) near the injection site (usually under the arm or near the neck)

*Rare adverse reaction (less than 1 in 1000):*

- Infection of the armpit lymph node, with swelling, abscess or ulcer.

*Very rare adverse reaction (less than 1 in 1 million)*

These conditions are usually associated with underlying inherited issues with the patient’s immune system.

- Disseminated BCG infection, where the vaccine bacteria spread throughout the body or to the bone occurs in 1-4 in 1 million doses.
- Anaphylaxis (a severe allergic reaction) to the BCG vaccine has been reported only 2-3 times in the 100 years the BCG vaccine has been used.

An excessive response to the BCG vaccine may result in an ulcer with some discharge. If this happens, you should encourage the ulcer to dry and avoid abrasion (by tight clothes, for example).

With the second BCG vaccination (if you were randomised to receive the BCG vaccine first), there is an increased risk that you may have an earlier, “accelerated” reaction which may begin within 24-48 hours of vaccination with toughening of the tissue followed by pustule formation in 5-7 days and healing within 10-15 days. Local skin lesions (ulceration and discharge) are more frequent in adults who have had a previous BCG vaccine than those who have never had BCG vaccine before. However, the risk of severe armpit lymph gland infection and disseminated BCG or reactivated tuberculosis disease has not been found to be more common in adults who have had previous BCG vaccine or positive tuberculosis screening tests.

Revaccination with the BCG as a part of this study does not align with current vaccination guidelines as most people only get vaccinated once as children, however it has been carefully considered upon systematic review of the literature to date. Adverse events will be actively monitored during the trial and medical review available for any participants who have concerns about their BCG vaccination site or scar.

If you have any of these side effects, or are worried about them, contact us.

Placebo injection

Having an injection can sometimes cause very minor pain from the needle or be uncomfortable.

Antibiotics

There are no major risks associated with the short course of antibiotics. As with other antibiotics, you may have some gastrointestinal symptoms (nausea, vomiting, stomach pain or diarrhoea) which typically clear up within a few days. Other (less likely) side effects may include a sore or irritated mouth, or gastrointestinal symptoms which persist longer than this. If you experience any of these side effects, or are worried about them, contact us.

While there’s no known impact of these antibiotics on the effectiveness of oral contraception, vomiting and diarrhoea can affect the effectiveness of oral contraceptives. If you are using oral contraceptive pills to prevent pregnancy, you should take extra (barrier) precautions while taking the antibiotics and for up to until the time of your next menstrual cycle.

Blood test

We will take 5 blood samples through the study. You may feel some discomfort during the blood collection. You may get some bruising, swelling or bleeding where the needle enters the skin. Some people can feel a little light-headed or faint when blood is taken.

Punch Biopsy

This will be performed by a trained doctor. A local anaesthetic is first given with a needle and can cause discomfort. The punch biopsy uses a small round cutting tool to take out a circle of skin for testing (about 3-4mm across, like a hole punch). The biopsy site may bleed at first but should stop quickly. On occasion, a stitch is needed, which would require you to come back after 7-10 days to have it removed.

10 **What will happen to my test samples?**

Your blood, stool and skin biopsy samples will be stored at the South Australia Health and Medical Research Institute (SAHMRI) for processing. Some deidentified data/samples may need to be subsequently analysed at ethically approved locations in Australia or overseas for certain analyses or for future research related to infectious diseases, immunology, or vaccines.

Deidentified means that a participant code (number) will be attached to the samples so that your name or other identifying information is not used to identify your samples. Only the research team will have access to the code.

Only SAHMRI members of the research team will be able to access your samples and will update reports on their location and processing. The freezers are located in secure areas at SAHMRI that are only accessible via key card access by authorised personnel.

Blood samples will be processed for analysis of the immune system. The immune system will be assessed by several methods, including:

- measurement of antibodies to BCG vaccination
- measurement of gene expression responses (which genes turned on and off) to vaccination
- identification and counting of different immune cells in the blood
- testing the function of immune cells in the blood by assessing activation markers and immune responses after *in vitro* stimulation
- assessing the structure of DNA in immune cells (epigenetic profiling)
- measurement of proteins in the blood
- measurement of bacterial metabolites (molecules normally produced by the gut bacteria) in the blood

Skin biopsy samples will be analysed by several methods:

- measurement of live BCG bacteria by culture and PCR
- measurement of gene expression in the sample
- visualisation of the sample under a microscope, to see which immune cells are in the immunisation site

Stool samples will be assessed using genomic techniques (microbial DNA sequencing) to identify how many bacteria are in the sample and what type of bacteria they are.

11 **What will happen to information about me? Confidentiality and data security**

Any information obtained for the purpose of this research project that can identify you will be treated as confidential and securely stored. It will be disclosed only with your permission, or as required by law.

Data generated by the analysis in this study will be stored in coded/re-identifiable form which will be password protected.

The principal investigators, co-investigators, study team, the CAHLN Human Research Ethics Committee and biostatistician will have access to your information as identified by your allocated study number.

The collected information will be stored secure at SAHMRI in locked filing cabinets or in restricted access folders on the Institute’s network drive and will only be accessible to the research team.

We are required to keep information collected for at least 15 years. The research information may be destroyed or kept indefinitely in secure storage after this time. Your information will be stored for future ethically approved research.

Any information we collect that can identify you will be treated as confidential and used only in this project unless otherwise specified. The information will be re-identifiable. This means that we will remove your name and give the information a special code number. Only the research team can match your name to the code number, if it is necessary to do so.

Research data collected in this study will be presented at scientific conferences and published in scientific journals. These will not identify any individuals. In any publication and/or presentation, data will be provided in such a way that you cannot be identified, except with your permission.

To advance science, medicine and public health, we will also need to share your de-identified data with other ethically approved research projects, data repositories, or medical journals. When we need to do this, we will remove identifying details such as your name, date of birth and address and give the data a special code number. Only the research team on this project will be able to match your name to their code number.

We will put security measures in place to protect your data if and when we give it to other people.

Despite our best efforts, there is a small chance that you could be re-identified by someone outside of this research project. In the unlikely event that this happens, someone from the research team will contact you. If, at any point, you think that your data may have been re-identified, please let us know.

In accordance with relevant Australian and/or South Australian privacy and other relevant laws, you have the right to request access to your personal information collected and stored by the research team. You also have the right to request that any information with which you disagree be corrected. Please contact the study team member named at the end of this document if you would like to access your information, however, individual test results cannot be given.

12 **What if I withdraw from this research project?**

If you decide to withdraw from the project, please notify a member of the research team before you withdraw. This notice will allow that person or the research supervisor to discuss any special requirements linked to withdrawing.

If you do withdraw your consent during the research project, the relevant study staff will not collect additional personal information from you, although personal information already collected will be retained to ensure that the results of the research project can be measured properly and to comply with law. You should be aware that data collected up to the time you withdraw will form part of the research project results. If you do not want them to do this, you must tell them before you join the research project. You will only receive payment for the visits you have attended.

13 **What happens if I am injured from taking part in the study?**

If you suffer any complications or injuries as a result of this research project, you should contact the study team as soon as possible and you will be assisted with arranging appropriate medical treatment. If you are eligible for Medicare, you can receive any medical treatment required to treat the injury or complication, free of charge, as a public patient in any Australian public hospital.

14 **Optional Consent - Use of Data and Sample for future ethically approved research**

We would like you to consider letting us store any samples and corresponding deidentified data that are left over from this project for future research. This is known as biobanking. A “bank” is the place where the health information and/or blood or tissue is stored.

We will use these samples and deidentified data in future ethically approved research.

If you give your permission, we may store your samples and deidentified data at SAHMRI. We may store them for an indefinite period.

We would like to use these samples and deidentified data in future ethically approved research.

We may need to move your samples and deidentified data to other locations. This includes overseas. We will make sure that we only send your data and samples to secure and ethically approved locations. However, any deidentified data and samples that we send overseas are not protected by Australian laws and regulations.

We will not contact you to use the samples in future research if you agree to this optional consent.

Please tick the appropriate box on the consent form to let us know if you consent to this.

15 **Complaints and contacts (Investigators and Ethics Committee)**

This project will be carried out according to the National Statement on Ethical Conduct in Human Research (2007) incorporating all updates. This statement has been developed to protect the interests of people who agree to participate in human research studies.

The study has been approved by the Central Adelaide Local Health Network Human Research Ethics Committee. If you wish to speak to someone not directly involved in the study about your rights as a volunteer, or about the conduct of the study, you may contact the CALHN Human Research Ethics Committee directly.

**Reviewing HREC approving this research, HREC Executive Officer details and Complaints Contact**

| **HREC Name** | **Central Adelaide Local Health Network Human Research Ethics Committee (CALHN HREC)** |
| --- | --- |
| **Contact** | **HREC Support Officer** |
| **Telephone** | **XXXXXXXXX** |
| **Email** | **XXXXXXXXX** |

If you want any further information concerning this project, you can contact the following people:

**Coordinating Principal Investigator**

| **Name** | **Prof David Lynn** |
| --- | --- |
| **Position** | **Principal Investigator** |
| **Telephone** | **XXXXXXXXX** |
| **Email** | **XXXXXXXXX** |

**Clinical Research Coordinator**

| **Name** | **Rochelle Botten** |
| --- | --- |
| **Position** | **Clinical Research Coordinator** |
| **Telephone** | **XXXXXXXXX** |
| **Email** | **avirs@sahmri.com** |

**Participant Information Sheet/Consent Form**

*Adult providing own consent*

| **Title** | A human experimental medicine study to assess whether the gut microbiota regulates specific immune responses to vaccination |
| --- | --- |
| **Short Title** | Antibiotics and Vaccine Immune Responses Study (AVIRS) – BCG Vaccine Arm |
| **Protocol Number** | 2023/HREC00066 |
| **Project Sponsor** | SAHMRI |
| **Co-ordinating Principal Investigator** | Prof David Lynn |
| **RAH Principal Investigator** | Dr Simone Barry |
| **Location** | Adelaide, SA |

**Consent Agreement**

• I have read the Participant Information Sheet or someone has read it to me in a language that I understand.

• I have had an opportunity to ask questions and I am satisfied with the answers I have received.

• The nature, purpose and risks of the research project have been explained to me. I understand them and agree to take part.

• I freely agree to participate in this research project as described and understand that I am free to withdraw at any time during the project without affecting my future health care.

• I understand that I will be given a signed copy of this document to keep.

• Your participation in this study shall not affect any other right you may have to compensation under common law.

**Optional Consent - Use of Sample and Data for future ethically approved research**

| □ I do | □I do not | consent to have my sample and corresponding deidentified data used for future ethically approved research |
| --- | --- | --- |

**Declaration by Participant – for participants who have read the information**

Name of Participant (please print) _________________________________________________

Signature _______________________________ Date _______________________________

**Declaration by Researcher^†^**

I have given an explanation of the research project, its procedures and risks and I believe that the participant has understood that explanation.

|  | Name of Researcher^†^ (please print) | |  | | |  |
| --- | --- | --- | --- | --- | --- | --- |
|  | | | | | |  |
|  | Signature |  | | Date |  |  |
|  | | | | | | |

^†^ An appropriately qualified member of the research team must provide the explanation of, and information concerning, the research project.

Note: All parties signing the consent section must date their own signature.
