# Supplementary material for: A randomised, placebo-controlled study to assess whether antibiotic-mediated disruption of the gut microbiota impairs BCG-induced specific and non-specific immunity in young, healthy adults: protocol for the Antibiotics and Vaccine Immune Responses Study (AVIRS)
Source: BMJ Open. 2026 Oct 14;16(10):e126924. doi: 10.1136/bmjopen-2026-126924 (PMC13691821; doi:10.1136/bmjopen-2026-126924)
Supplement: Supplementary file 5 [file bmjopen-16-10-s002.docx]

AVIRS Data and Safety Monitoring Board Charter

TITLE: A human experimental medicine study to assess whether the gut microbiota regulates specific and non-specific immune responses to vaccination

SHORT TITLE: Antibiotics and Vaccine Immune Responses Study (AVIRS)

PROTOCOL NUMBER: 037865

PRINCIPAL INVESTIGATORS: Prof David Lynn and Dr Simone Barry

VERSION: 1.0

SUPPORTED BY NHMRC

# REVISION HISTORY

| Version No. | Date | Summary of Changes |
| --- | --- | --- |
| 1.0 | 14 March 2023 | Initial version |

AGREEMENT

*The members of the DSMB must sign the charter to indicate their approval of the content and agreement to adhere to the terms of this charter.*

| Name, Title and Affiliations | DSMB Role  (*e.g. chair, clinical expert, biostatistician, independent*  *statistician, ex officio DSMB member)* | Signature | Date |
| --- | --- | --- | --- |
| TBC | Chair |  |  |
| TBC | Clinical expert |  |  |
| TBC | Clinical expert |  |  |

Contents

[REVISION HISTORY	1](#_Toc129270646)

[1.	INTRODUCTION	4](#_Toc129270647)

[2.	DSMB MEMBERSHIP	4](#_Toc129270648)

[2.1	Exclusion of Conflicts of Interest	4](#_Toc129270649)

[2.2	Resignation/Termination of DSMB Member and Replacement	5](#_Toc129270650)

[3.	RESPONSIBILITIES OF THE DSMB	5](#_Toc129270651)

[3.1	Stewardship of the study	5](#_Toc129270652)

[3.2	Safety Monitoring	6](#_Toc129270653)

[4.	FREQUENCY AND FORMAT OF MEETINGS	6](#_Toc129270654)

[4.1	Initial Meeting	6](#_Toc129270655)

[4.2	First Review Meeting	6](#_Toc129270656)

[4.3	Subsequent Review Meetings	6](#_Toc129270657)

[4.4	Ad Hoc Meetings	7](#_Toc129270658)

[5.	CONDUCT OF MEETINGS	7](#_Toc129270659)

[5.1	Open Session	7](#_Toc129270660)

[5.2	Closed Session	7](#_Toc129270661)

[5.3	Meeting Attendance and Quorum	7](#_Toc129270662)

[5.4	Meeting Deliberations	7](#_Toc129270663)

[5.5	DSMB Recommendations	7](#_Toc129270664)

[5.6	Meeting Minutes	8](#_Toc129270665)

[5.7	Study Publications	8](#_Toc129270666)

[6.	DATA MONITORING AND REPORTS	8](#_Toc129270667)

[6.1	Data Analysis and DSMB Reporting	8](#_Toc129270668)

[7.	CONFIDENTIALITY	10](#_Toc129270669)

[8.	COMMUNICATIONS	10](#_Toc129270670)

[9.	LIABILITY STATUS OF THE DSMB	10](#_Toc129270671)

[10.	KEY TERMS	10](#_Toc129270672)

[11.	DSMB Recommendation Template	11](#_Toc129270673)

# 1. INTRODUCTION

This charter is for the Data and Safety Monitoring Board (DSMB) for the experimental medicine study entitled: A human experimental medicine study to assess whether the gut microbiota regulates specific and non-specific immune responses to vaccination.

This charter defines the responsibilities of the DSMB, its membership, and the purpose and timing of its meetings. The Charter also provides the procedures for ensuring confidentiality and proper communication, the statistical monitoring procedures to be implemented by the DSMB, and an outline of the content of the reports that will be provided to the DSMB.

See Section 10 for a list of definitions of the terms and abbreviations used in this charter.

# 2. DSMB MEMBERSHIP

The DSMB consists of the following independent members who collectively have experience in the clinical area of interest, biostatistics and randomised clinical studies. A quorum will require all 3 members.

The DSMB will consist of:

Voting members

| **DSMB Role** | **Name and Title** | **Affiliation / Institution** | **Email** | **Summary of expertise** |
| --- | --- | --- | --- | --- |
|  |  |  |  |  |
|  |  |  |  |  |
|  |  |  |  |  |

Non-voting members:

The Medical monitor will prepare confidential reports for the Closed Session. Study investigators will not be members of the DSMB.

## 2.1 Exclusion of Conflicts of Interest

The DSMB membership will be restricted to individuals free of any conflicts of interest. Even the appearance of conflict of interest among DSMB members must be avoided. Any DSMB member who has, or develops, a significant conflict of interest should resign from the DSMB.

The DSMB members must disclose conflicts of interest to fellow members. Declaration of a conflict of interest is an ongoing process; it will be completed at the time of joining the DSMB and prior to each DSMB meeting and will be recorded in the meeting minutes.

Conflicts of interest can include:

- Stock ownership in any commercial companies involved
- Stock transaction in any commercial company involved (if previously holding stock)
- Frequent speaking engagements on behalf of the intervention
- Career tied up in a product or technique assessed by the study
- Hands-on participation in the study
- Involvement in the running of the study
- Emotional involvement in the study
- Intellectual conflict (e.g. strong prior belief in the study’s experimental arm)
- Involvement in regulatory issues relevant to the study procedures
- Investment (financial or intellectual) in competing products
- Involvement in the publication

The DSMB will function independently of all other individuals and bodies associated with the conduct of the study.

## 2.2 Resignation/Termination of DSMB Member and Replacement

DSMB membership is for the duration of the clinical study. If any members leave the DSMB during the course of the study, the Principal Investigator will promptly appoint their replacement with agreement from the remaining members of the DSMB. Further appointments may be made to the DSMB if members believe additional expertise is required. DSMB members can decide to terminate the membership of a DSMB member based on a simple vote in case of non-performance or other significant reasons as determined by a majority of the DSMB.

# 3. RESPONSIBILITIES OF THE DSMB

## 3.1 Stewardship of the study

The DSMB is responsible for the stewardship of the study over all participating sites or institutions. The stewardship includes review of participant recruitment, accrual, retention, and withdrawal. It further involves oversight of participant management, adherence to protocol-specified regimens, and procedures for data management and quality control.

The DSMB will be responsible for safeguarding the interests of participants by assessing the safety of the interventions during the study, and the general progress of the study.

Specifically, the role of the DSMB will be to:

- Monitor and review participant safety in the study (including evidence for treatment harm, e.g. toxicity data, safety events)
- Review participant recruitment, accrual, retention, study withdrawal, serious breaches, and protocol deviations

This responsibility will be exercised by providing recommendations about continuing, modifying or stopping the study, including recommendation to publish safety data. To contribute to enhancing the integrity of the study, the DSMB may also formulate recommendations relating to the selection/recruitment/retention of participants, participant management, and the procedures for data management and quality control.

The DSMB will be advisory to the Principal Investigators. The Principal Investigators hold the ultimate responsibility for decisions regarding the study.

## 3.2 Safety Monitoring

The DSMB is responsible for safeguarding the interests of participants by assessing the safety of the interventions during the study.

At least one DSMB member will be an expert in the potential safety outcomes of the study. If important safety items are not considered in the reporting of safety data, the DSMB may request to change or add items to be included.

The study has two mechanisms for monitoring safety:

a) During recruitment of the study, the medical monitor (who is an Associate Investigator) will review all adverse events on a weekly basis in a semi blinded fashion (group 1, 2, 3 and 4) and report any concerns to the Principal Investigator and/or to the DSMB members to act promptly if there are concerns. The medical monitor has complete discretion with what to do with these safety data. The medical monitor may choose to be unblinded if it helps any decision. This role will cease once recruitment is complete.
b) A formal DSMB meeting will be convened quarterly to review safety data.

# 4. FREQUENCY AND FORMAT OF MEETINGS

DSMB meetings are planned to occur every quarter.

## 4.1 Initial Meeting

The initial meeting of the DSMB will review the role and functioning of the DSMB, discuss the format and content of the DSMB reports and review scientific and ethical issues relating to the design and conduct of the study.

## 4.2 First Review Meeting

The first DSMB meeting will occur 3 months after the first participant is enrolled. At this meeting, the following will be reviewed:

- Data related to common reactions to the antibiotics schedule
- Data related to uncommon and rare side effects of the antibiotics schedule
- Data related to common reactions to BCG vaccination within the first 2 weeks post vaccination
- Data related to uncommon and rare side effects of BCG vaccination within the first 2 weeks post vaccination
- Data relating to study conduct, including participants disposition and protocol deviations
- Data completeness

## 4.3 Subsequent Review Meetings

Subsequent fixed term DSMB meetings will occur quarterly. At these meetings, the following will be reviewed:

- Data related to common reactions to the antibiotics schedule
- Data related to uncommon and rare side effects of the antibiotics schedule
- Data related to common reactions to BCG vaccination within the first 2 weeks post vaccination
- Data related to uncommon and rare side effects of BCG vaccination within the first 2 weeks post vaccination
- Data related to common reactions to BCG challenge within the first 2 weeks post vaccination
- Data related to uncommon and rare side effects of BCG challenge within the first 2 weeks post vaccination
- Data relating to study conduct, including recruitment rate, participants disposition and protocol deviations
- Data completeness

## 4.4 Ad Hoc Meetings

Additional *ad hoc* meetings of the DSMB may be scheduled if requested by either the Principal Investigators or the DSMB.

# 5. CONDUCT OF MEETINGS

Meetings will consist of an open session and a closed session.

## 5.1 Open Session

Members of the AVIRS Research team, which includes the Principal Investigators, will meet with the DSMB and the medical monitor who prepared the DSMB report at the commencement of each meeting. This “*Open Session”* provides the DSMB an opportunity to query the Research team members about issues that have arisen during the review of the data. Once the DSMB members are satisfied that all their queries have been addressed, the Research team members will then leave the meeting to enable the confidential *Closed Session* of the DSMB to commence. The Principal Investigators will remain available to return, if required, to assist with any questions.

## 5.2 Closed Session

The medical monitor who prepared the DSMB report will remain for the first part of the closed session in order to take the DSMB through the report and answer questions if required. The medical monitor will then leave the closed session. The remainder of the closed session will involve only DSMB members to allow discussion of confidential data from the clinical study.

## 5.3 Meeting Attendance and Quorum

The minimum number of members in attendance for the DSMB to be quorate for decision-making is 3.

If the report is circulated before the meeting, DSMB members who will not be able to attend the meeting may pass comments to the DSMB Chair for consideration during the discussions.

If a member does not attend a meeting, it should be ensured that the member is available for the next meeting. If a member does not attend a second meeting, they should be asked if they wish to remain part of the DSMB. If a member des not attend a third meeting, they should be replaced.

## 5.4 Meeting Deliberations

The Chair will facilitate and summarise discussions and will encourage consensus. Following its review of the data, the DSMB will reach consensus on its list of recommendations. Consensus will be determined through formal voting.

## 5.5 DSMB Recommendations

The recommendations provided by the DSMB may include:

1. Continuing the study unchanged
2. Continuing the study with modifications; or
3. Terminating the study.
4. Publishing safety data

The DSMB may also make recommendations about other aspects of the study such as the recruitment of participants and the conduct of the study. All recommendations will be sent to the Principal Investigator promptly, within 2 weeks.

In the event that the DSMB recommends the early termination of the study, the final decision to stop the study early or modify the study protocol will be made by the Principal Investigator. If this situation arises at any time, the decision of the Principal Investigator will be discussed with the DSMB immediately.

The DSMB will be advisory to the Principal Investigator. The Principal Investigator holds ultimate responsibility for decisions regarding the study.

Response to the DSMB’s recommendations:

- If the Principal Investigator does not agree with the DSMB recommendations, a memo justifying the reasons for not complying with the recommendations of the DSMB will be promptly forwarded to the DSMB within 2 weeks,
- If the DSMB is not satisfied with the Principal Investigator response to their recommendations, the DSMB will promptly notify HREC or regulatory authorities, within 2 weeks.

Note that in the event that the Principal Investigator wishes to remove one or more DSMB members*,* a memo justifying the reasons for this will be promptly forwarded to the DSMB and to the HREC, i.e. within 2 weeks*.*

## 5.6 Meeting Minutes

The DSMB will have minutes taken for both the Open and Closed meetings. Meeting minutes should be signed by the DSMB Chair and distributed as soon as possible after the DSMB meeting.

## 5.7 Study Publications

The DSMB may be sent copies of accepted papers for their information.

DSMB members should be named and their affiliations listed in the main report/publication. A brief summary of the timings and conclusions of the DSMB meetings should be included in the body of the main study paper.

# 6. DATA MONITORING AND REPORTS

## 6.1 Data Analysis and DSMB Reporting

6.1.1 Open and Closed Reports

The medical monitor will undertake the data analysis and the creation of the DSMB reports. The medical monitor preparing the information for the DSMB will prepare two reports, an “open” and a “closed” report (see sections 6.1.1 and 6.1.2 below). Both the open report and the confidential closed report will be sent to the DSMB members for review 7 days prior to the scheduled meeting.

The open report will also be circulated to the AVIRS Research team which will meet shortly after the DSMB to discuss any recommendations made by the DSMB along with any other study related issues.

6.1.1.1 Open Reports

Open reports will contain the following information:

- Study number and title.
- Brief summary of the study design and progress.
- Details of any protocol amendments since the previous report
- Status of accrual (actual vs target recruitment)

If accrual is slower than expected include a plan for increasing enrollment.

- Summary of patients disposition (including data completeness)
- Summary of baseline characteristics
- Summary of common reactions to antibiotic dosage within 1 week (if recruitment is still ongoing at this time point)
- Summary of uncommon and rare side effects of antibiotic dosage within 1 week (if recruitment is still ongoing at this time point)
- Summary of common reactions to vaccination within the first 2 weeks post vaccination (if recruitment is still ongoing at this time point)
- Summary of uncommon and rare side effects of vaccination within the first 2 weeks post vaccination (if recruitment is still ongoing at this time point)
- Summary of common reactions to vaccination challenge within the first 2 weeks post vaccination (if recruitment is still ongoing at this time point)
- Summary of uncommon and rare side effects of vaccination challenge within the first 2 weeks post vaccination (if recruitment is still ongoing at this time point)
- Summary of protocol deviations
- Details of serious breaches

Data in this report will be presented across all participants with NO reference to treatment group.

6.1.1.2 Closed Reports

Closed reports will contain the following information:

- Information as above for the open report – including treatment groups

The format of these reports will be determined by the DSMB in consultation with the medical monitor preparing the report. Information will be presented by pseudo-labelled treatment group (e.g. “A” and “B”). In some circumstances, unintended unblinding may occur if certain reported parameter values are expected to be associated with the interventions, such as common reactions to vaccination. In such circumstances, the need for presenting data by treatment group should be carefully considered among members of the DSMB.

Of note, un-blinding can occur in the following scenarios:

- If requested by the DSMB
- If requested by the medical monitor during the weekly reporting period in the recruitment phase

Note the medical monitor or the DSMB may request unblinding to inform a final decision as to whether or not to "stop" the study and disseminate results. The decisions as to whether or not to request unblinding are inevitably subjective and up to the medical monitor or DSMB. This decision would be driven by the magnitude of any increased harm between groups and the current external evidence for likely harm.

The key to identify the treatment regimens may be supplied by the statistician if requested by the DSMB. Additional information may be presented in subsequent reports if specifically requested by the DSMB.

# 7. CONFIDENTIALITY

All DSMB reports will remain confidential until the end of the study. Details of DSMB discussions and draft reports will remain confidential until formally delivered to the Principal Investigator.

After each meeting, the DSMB members should store the papers safely after each meeting so that they may check the next report against them. After the study is reported the DSMB members should destroy all interim reports.

# 8. COMMUNICATIONS

At any time during the study, regulatory authorities, the Human Research Ethics Committee, the AVIRS Research Team or any other body or individual involved with the conduct of the study may seek the advice of the DSMB about any concern that they may have about the conduct, outcome or continuation of the study. Any such requests should be forwarded in writing to the DSMB Chairperson at the address provided above.

If any suspected unexpected serious adverse events occur, which are thought to relate to the experimental treatment, the Chair of the DSMB will be notified within 72 hours. The Chair will then decide whether an additional meeting of the DSMB should be held.

# 9. LIABILITY STATUS OF THE DSMB

As the DSMB is an advisory body alone, its members are not liable for damage, harm, morbidity or mortality to recruited patients.

# 10. KEY TERMS

DSMB: A Data Safety Monitoring Board is an independent data-monitoring group that may be established by those responsible for study conduct to monitor the progress of a clinical study with focus on potentially arising safety issues.

SERIOUS BREACH: A breach of Good Clinical Practice or the protocol that is likely to affect to a significant degree: the safety or rights of a study participant, or the reliability and robustness of the data generated in the clinical study. Note: this guidance's definition of serious breach differs from the definition in the Australian Code for the Responsible Conduct of Research and is about deviations from the requirements of Good Clinical Practice or the clinical studies protocol.

# 11. DSMB Recommendation Template

AVIRS DSMB Recommendation

PROTOCOL TITLE: A human experimental medicine study to assess whether the gut microbiota regulates specific and non-specific immune responses to vaccination

SHORT TITLE: Antibiotics and Vaccine Immune Responses Study (AVIRS)

PROTOCOL NUMBER: 037865

VERSION: XX

DSMB Meeting Date:

To: [PI names] and [HREC]

From: [name] AVIRS DSMB Chairperson

Re: DSMB Recommendation

I would like to inform you of the recommendation made by the Data and Safety Monitoring Board for AVIRS. The DSMB convened on [date]. After reviewing the data, the DSMB recommends:

☐ Continue the study unmodified (see Comments, if applicable)

☐ Continue the study and amend the protocol as described: (see Comments)

☐ Schedule a meeting with the AVIRS Research team (see Comments)

☐ Discontinue the study (see Comments)

☐ Other (see Comments)

Comments: ________________________________________________________________________

__________________________________________________________________________________

_____________________________________________________________________­­­_____________

Signature: ____________________

DSMB Chairperson

Date: ____________________
